# Modulation of activation-associated host cell gene expression by the apicomplexan parasite *Theileria annulata*

**DOI:** 10.1111/j.1462-5822.2012.01809.x

**Published:** 2012-05-23

**Authors:** Zeeshan Durrani, William Weir, Sreerekha Pillai, Jane Kinnaird, Brian Shiels

**Affiliations:** Institute of Infection, Immunity & Inflammation College of Medical, Veterinary and Life Sciences University of Glasgow Bearsden Road Glasgow G61 1QH Scotland UK

## Abstract

Infection of bovine leucocytes by *Theileria annulata* results in establishment of transformed, infected cells. Infection of the host cell is known to promote constitutive activation of pro-inflammatory transcription factors that have the potential to be beneficial or detrimental. In this study we have compared the effect of LPS activation on uninfected bovine leucocytes (BL20 cells) and their *Theileria*-infected counterpart (TBL20). Gene expression profiles representing activated uninfected BL20 relative to TBL20 cells were also compared. The results show that while prolonged stimulation with LPS induces cell death and activation of NF-κB in BL20 cells, the viability of *Theileria*-infected cells was unaffected. Analysis of gene expression networks provided evidence that the parasite establishes tight control over pathways associated with cellular activation by modulating reception of extrinsic stimuli and by significantly altering the expression outcome of genes targeted by infection-activated transcription factors. Pathway analysis of the data set identified novel candidate genes involved in manipulation of cellular functions associated with the infected transformed cell. The data indicate that the *T. annulata* parasite can irreversibly reconfigure host cell gene expression networks associated with development of inflammatory disease and cancer to generate an outcome thatis beneficial to survival and propagation of the infected leucocyte.

## Introduction

The protozoan parasites *Theileria annulata* and *T. parva* cause economically important lymphoproliferative disorders of cattle in developing countries of the Old World (Irvin and Morrison, 1987). In the laboratory they provide a unique model to investigate how a simple eukaryotic cell can direct gene expression and induce proliferation of another, more complex, eukaryotic host cell. Following invasion of bovine leucocytes by tick-inoculated sporozoites, rhoptries and microspheres discharge and the enveloping host cell membrane is disrupted. The intermediate trophozoite stage is rapidly surrounded by an array of host microtubules and within 48 h differentiates into the macroschizont stage (Shaw and Tilney, 1992). This differentiation event is accompanied by parasite-dependent transformation of the host cell (reviewed in Dobbelaere and Heussler, 1999), which *in vivo* manifests in exponential proliferation of infected leucocytes over 4–5 days, followed by dissemination to a wide range of tissues. In acute cases of disease, disorganization and destruction of the lymphoid system occurs and death is often caused by massive pulmonary oedema associated with migration of infected cells to the lungs (Irvin and Morrison, 1987).

Transformation of *T. annulata-* and *T. parva*-infected leucocytes is known to be dependent on constitutive activation of bovine transcription factors and associated signal transduction pathways (reviewed in Dobbelaere and Heussler, 1999; Shiels *et al*., 2006). Activation of AP-1, ATF-2, cMyc, NF-κB and STAT3 have all been documented and are likely to be responsible for many of the phenotypic alterations displayed by the infected cell. However, in addition to being beneficial, constitutive transcription factor activation has the potential to be detrimental to survival of the infected cell or host. For example, constitutive activation of c-Jun (a dominant component of AP-1 transcription factor), is thought to play an important role in metastasis of *Theileria*-infected cells (Adamson *et al*., 2000), while activation of NF-κB confers protection against apoptosis (Heussler *et al*., 1999). However, both these factors can be pro-apoptotic (Dunn *et al*., 2002; Dutta *et al*., 2006) by upregulating genes encoding death-inducing signals, including pro-inflammatory cytokines. It has been predicted that NF-κB-dependent regulation of inflammatory genes contributes to the pathogenesis of theileriosis (Heussler *et al*., 2002).

A recent study demonstrated that only a proportion of leucocytes infected with *T. parva* sporozoites successfully progress to proliferation, while the rest undergo apoptosis (Rocchi *et al*., 2006). Thus, events that are detrimental to the infected cell can occur following sporozoite invasion and transformation is not guaranteed. In addition, although basal expression of inflammatory/cellular defence genes such as *TNFα*, *iNOS* and *ISG15* can be detected (Sager *et al*., 1997; Oura *et al*., 2006), *Theileria*-infected cells are refractory to LPS-mediated stimulation of high-level expression, despite constitutive activation of transcription factors (e.g. AP-1 and NF-κB) associated with this event. Furthermore, parasite-mediated repression of highly abundant mRNA levels has been indicated for the ISGylation pathway genes, *UBP43* and *ISG15* (Oura *et al*., 2006) whose expression, like *TNFα* and *iNOS,* can be induced by activated NF-κB (Li *et al*., 2001; Liu *et al*., 2011). Taken together, these studies predict that *Theileria* parasites must direct gene expression networks associated with activation of host cell transcription factors to promote an outcome that favours establishment of the transformed cell.

In this study we have conducted a comprehensive analysis of expression profiles associated with LPS stimulation of the uninfected bovine lymphosarcoma cell line BL20 (Olobo and Black, 1989) and the *T. annulata*-infected counterpart, TBL20 (Shiels *et al*., 1986). These lines were chosen as they provide uniform cellular populations to allow robust detection of differences in gene expression associated with altered cellular phenotypes. Moreover, TBL cells have been used to investigate infection-associated phenotypes (Shiels *et al*., 1986; Baylis *et al*., 1995) and are known to be dependent on a viable parasite for survival, as they undergo apoptosis when the macroschizont is killed by BW720c (buparvaquone) (Dobbelaere and Heussler, 1999). The results of our analysis indicate that while the modulated gene expression profiles of LPS-stimulated, uninfected BL20 and infected TBL20 cells significantly overlap, a major reorganization of activated gene expression is orchestrated by the parasite-infected cell to promote its establishment and alter receptiveness to extrinsic activation signals.

## Results and discussion

### Differential growth and activation response of BL20 and TBL20 cells to stimulation by LPS

**Figure 1:**
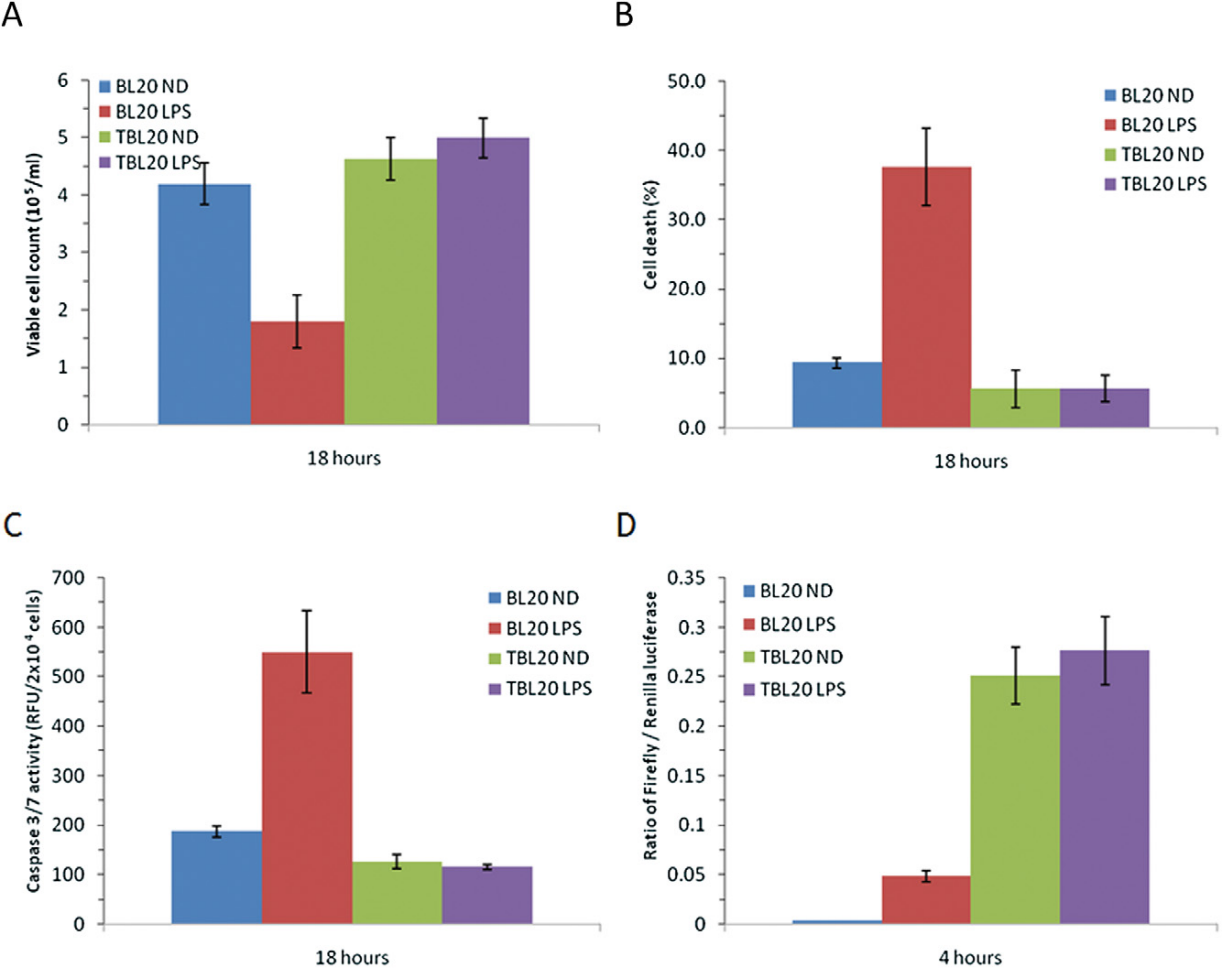
A. Viability of BL20 and TBL20 cells following LPS stimulation. Cell viability of BL20 and TBL20 cell cultures was assessed using Trypan Blue exclusion in the presence of either LPS (1 μg ml^−1^) or no drug DMSO (1 μl ml^−1^) vehicle control (ND) at 18 h post treatment. Counts are expressed as group mean ± SD, *n* = 3. The number of viable cells in BL20 LPS-treated cultures was significantly lower than BL20 no drug (ND) and TBL20 cultures (*t*-test, *P* < 0.01); TBL20 compared with TBL20-LPS cultures showed no significant difference. B. Percentage dead cells in BL20 and TBL20 cultures following LPS stimulation. Cell viability of BL20 and TBL20 cell cultures was assessed using Trypan Blue exclusion in the presence of either LPS (1 μg ml^−1^) or no drug (ND) at 18 h post treatment. The percentage dead cells in each culture condition are expressed as group mean ± SD, *n* = 3. The percentage dead cells in BL20 LPS-treated cultures was significantly greater than BL20 no drug (ND) and TBL20 cultures (*t*-test, *P* < 0.01); TBL20 compared with TBL20-LPS cultures showed no significant difference. C. Caspase 3/7 activity in BL20 and TBL20 cells following LPS stimulation. Caspase 3/7 activity was determined after incubation of cells in the presence of either LPS (1 μg ml^−1^) or the no drug DMSO (1 μl ml^−1^) vehicle control (ND) for 18 h. Results are expressed as relative fluorescence units (mean ± SD, *n* = 3). Caspase activity was significantly greater in BL20-LPS compared with BL20 no drug (*t*-test, *P* < 0.01); TBL20 compared with TBL20-LPS cultures showed no significant difference. D. NF-κB-dependent reporter activity in BL20 and TBL20 cells following LPS stimulation. Levels of NF-κB activation in *Theileria annulata*-infected TBL20 cells and uninfected BL20 cells following stimulation with LPS are shown relative to untreated controls. Cells were transiently co-transfected with an NF-κB promoter/Firefly luciferase reporter construct and a control Renilla luciferase construct. Twenty-four hours after transfection, cells were stimulated either with LPS (1 μg ml^−1^) or no drug DMSO (1 μl ml^−1^) vehicle control (ND) for 4 h before extraction and assay for luciferase activity. NF-κB promoter activity is expressed as the ratio of NF-κB Firefly luciferase/Renilla activity (mean ± SEM, *n* = 4). NF-κB activity was significantly higher in BL20-LPS compared with BL20 no drug (*t*-test, *P* < 0.005); TBL20 compared TBL20-LPS cultures showed no significant difference.

From the results of previous studies we postulated that uninfected cells could display a detrimental response to prolonged stimulation with an inflammatory mediator, while *Theileria*-infected cells would be refractory to such stimulation. To test this postulation, BL20 and TBL20 cultures were treated with LPS and the number of viable and dead cells estimated after 18 h. As shown by Fig 1A, the number of viable BL20 cells following stimulation with LPS was significantly lower (2.3-fold) relative to the un-stimulated control. This loss of growth potential was accompanied by a 4-fold increase in the percentage of non-viable cells (Fig 1B) and a 2.9-fold increase in caspase 3/7 activity (Fig 1C). Infected TBL20 cells, in contrast, showed no significant difference between LPS-stimulated and control cultures. These results suggest that TBL20 cells are resistant to a detrimental effect of stimulation with LPS (18 h).

In addition to growth analysis, infected and uninfected cells were assayed for NF-κB reporter activity. NF-κB reporter activity was significantly elevated in uninfected BL20 cells following LPS stimulation and, while basal reporter activity was higher in TBL20, no significant elevation in constitutive NF-κB activity following LPS treatment was detected (Fig 1C). Confirmation of NF-κB activation by LPS in BL20 was obtained by detection of the p65 subunit of NF-κB in the nucleus and IKKγ signalosome complexes in the cytoplasm (Fig. S1). The finding that NF-κB activation occurs in LPS-stimulated BL20 and is constitutively activated in infected TBL20 suggests that for this transcription factor, at least, TBL20 cells may avoid a potentially detrimental response by modulating the outcome of the activation event.

### Activation of BL20 cells by LPS gives rise to gene expression changes that show significant similarity to changes associated with infected TBL20 cells

To compare bovine gene expression changes associated with activation of BL20 cells with LPS relative to changes associated with infected TBL20 cells, microarray analysis was performed. The oligonucleotide microarray platform represented all confirmed and putative bovine mRNA features available (31 481) and provided an increase in gene number relative to the BoMP array utilized by Jensen *et al*. (2008). A subset of 19 777 genes was used for further analysis, as this represents sequences on the array that were found to be present in the current bovine genome assembly or RefSeq database. The array was hybridized with cDNA representing RNA from cultures of BL20 and TBL20 cells before and at 4 and 18 h post LPS stimulation. Differential gene expression on the normalized data set was assessed by pair-wise comparison using Rank Product Analysis (Breitling *et al*., 2004) and differences were considered to be significant at a false discovery rate (FDR) of less than 5% and a fold change greater than 2.

The results showed that TBL20 cells were refractory to major LPS-induced global expression changes, as only a small subset of genes (214 genes) showed significantly altered expression following stimulation of TBL20 with LPS, whereas a total of 2180 genes were identified as altered by LPS stimulation of BL20 (BL20-LPS data set). Thus, as implied from previous studies, *T. annulata-*infected leucocytes are associated with major suppression of lipopolysaccharide (LPS) inducible gene expression (Sager *et al*., 1997; Oura *et al*., 2006; Jensen *et al*., 2008).

**Figure 2:**
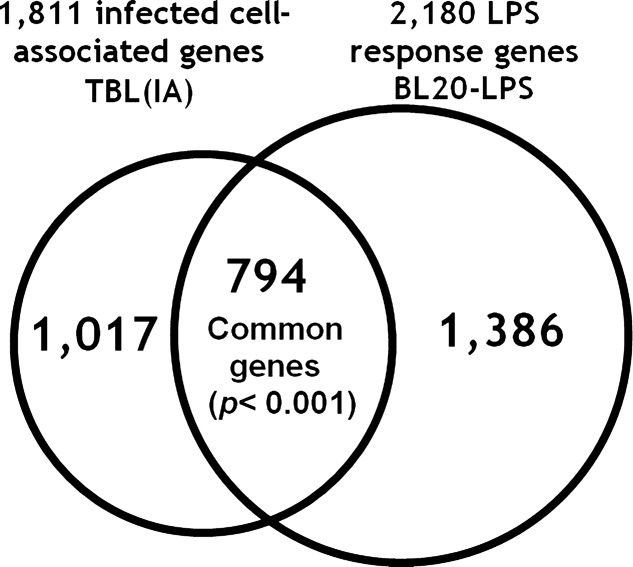
Identification of a significant overlap between TBL20(IA) and BL20-LPS data sets. Analysis of the lists of differentially expressed genes (FDR < 5%, FC > 2) generated from each experimental condition led to the identification of a set of 794 genes common to data sets representing parasite-infected cells and LPS treatment of uninfected cells. The overlap between two data sets was found to be highly significant (*P* < 0.001).

A large number of genes (1811) showed changes in expression that were associated with infection of BL20 cells by *T. annulata* [the infection-associated data set, TBL20(IA)]. Comparison of the BL20-LPS data set and the TBL20(IA) data set showed the common overlapping gene list (794 genes) was significantly larger than would be expected to occur by chance (Fig 2). This result demonstrates that even though it is largely unresponsive to LPS, the gene expression profile of the parasite-infected cell is related to an activated state induced by a classical inflammatory mediator. Pathway analysis of the BL20-LPS data set confirmed that enrichment for inflammatory response genes was of greatest significance (data not shown). As infection of the host leucocyte by *T. annulata* results in constitutive activation of transcription factors such as NF-κB (Oura *et al*., 2006) and AP1 (Baylis *et al*., 1995) that are important regulators of the inflammatory response, it was of interest to investigate whether the outcome of their activation is altered in the parasite-infected cell and results in a prediction of phenotype that is favourable to the establishment of infection.

### Gene expression changes in response to activation of BL20 cells by LPS are modulated in *T. annulata*-infected TBL20 cells

To investigate whether gene expression changes associated with cellular activation of BL20 cells are modulated in parasite-infected cells, gene expression in TBL20 cells was contrasted with that of uninfected, activated cells. To achieve this, the set of differentially expressed genes in the BL20/BL20-LPS data set was compared against the expression values from the entire TBL20 data set. For an LPS stimulated gene to be considered as a candidate for modulation by infected cells, the expression level in the infected cell had to show a difference of > 2-fold compared with that of the LPS stimulated BL20 cell (BL20-LPS: TBL20 ratio > 2), or show a contrasting Rank Products result. This resulted in the generation of an LPS response, infected cell-modulated gene list of 1214 genes (hereafter known as the infected cell-modulated gene list). Of these, 198 were more highly expressed in TBL20 cells than in BL20 LPS-treated cells, while a considerably larger number, 1016 genes, were repressed in TBL20 cells. As expected (see above), the vast majority (> 95%) of the genes in the list of 1214 showed no significant change in their expression level in TBL20 cells stimulated with LPS (FDR < 5%).

**Figure 3:**
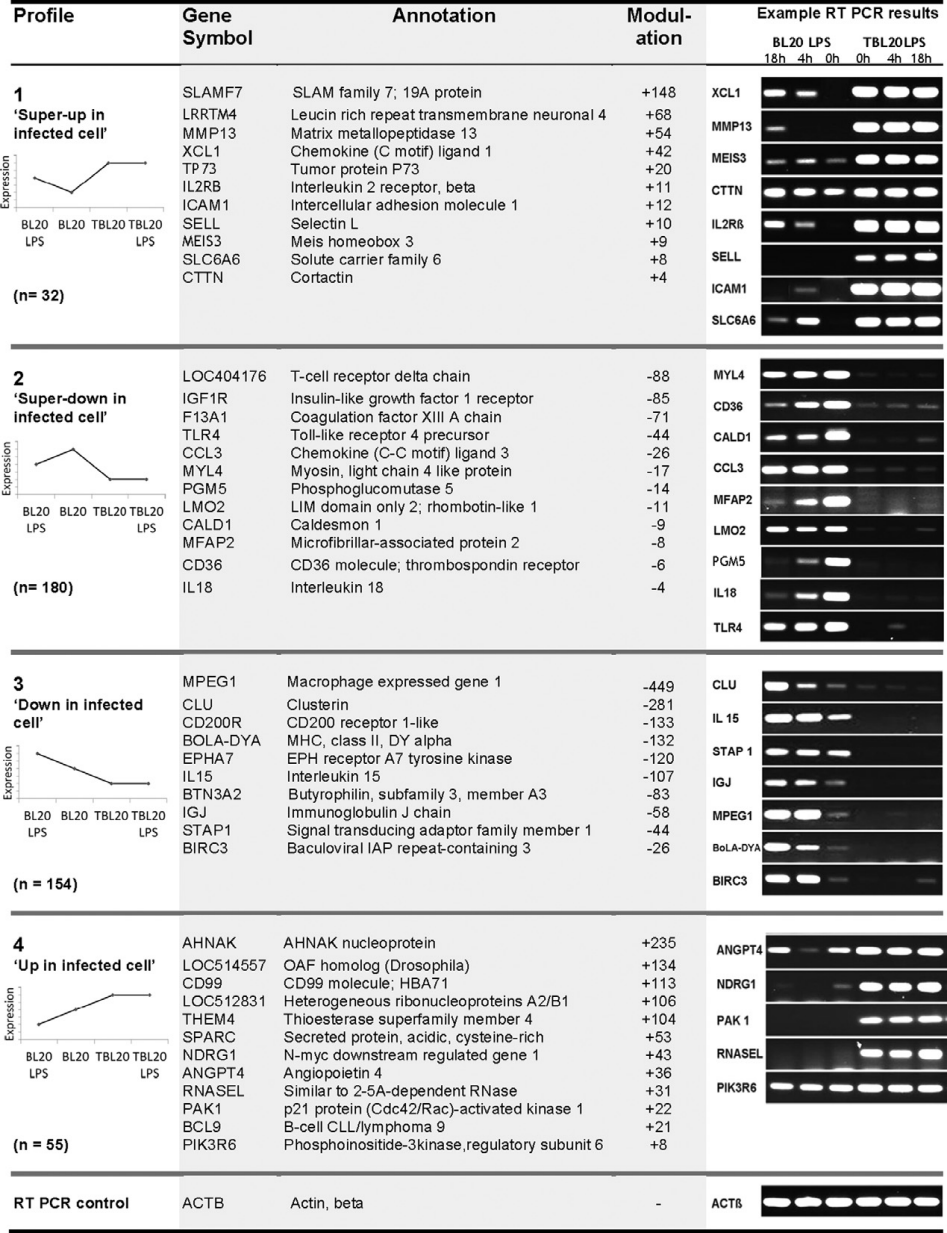
Expression profiles of BL20-LPS-response genes showing a modulated response in *Theileria*-infected cells. The different gene expression response profiles for TBL20 and BL20 cells obtained before and following LPS treatment are shown diagrammatically to the left, together with the total number of genes in that profile. Denoted genes (middle) represent top-ranking/selected (based on predicted function) differentially expressed genes within the profile. Semi-quantitative RT-PCR validation for a panel of the selected genes is shown on the right.

Further analysis of the data set was conducted by incorporating data obtained from an independent study on the response of host cell gene expression following treatment of TBL20 with BW720c for 48 h (J. Kinnaird *et al*., in preparation). This enabled identification of expression changes that are more likely to be under direct control of a viable parasite, i.e. if an infection-associated modulation was reversed upon BW720c treatment (see Tables S1–S8). In addition, analysis of expression values for the set of 1214 genes across all six cellular conditions partitioned the gene list into four pairs of reciprocal profiles with evidence of modulated expression in TBL20 relative to BL20-LPS (Figs 3 and for selected genes and Tables S1–S8 for full gene lists of each profile). To provide insight into potential biological relevance of the infected cell-associated modulation, the data set for each profile was subjected to pathway analysis to investigate enrichment for predicted molecular and cellular functions. Functional annotation derived from the bovine RefSeq database was also utilized. For validation of the microarray results, between three and nine genes were chosen from each profile for semi-quantitative reverse transcription PCR (RT-PCR). These were selected based, primarily, on annotation that could provide possible insight into the infected cell modulation event. Two validated genes of interest were then selected for further validation by qRT-PCR.

**Figure 4:**
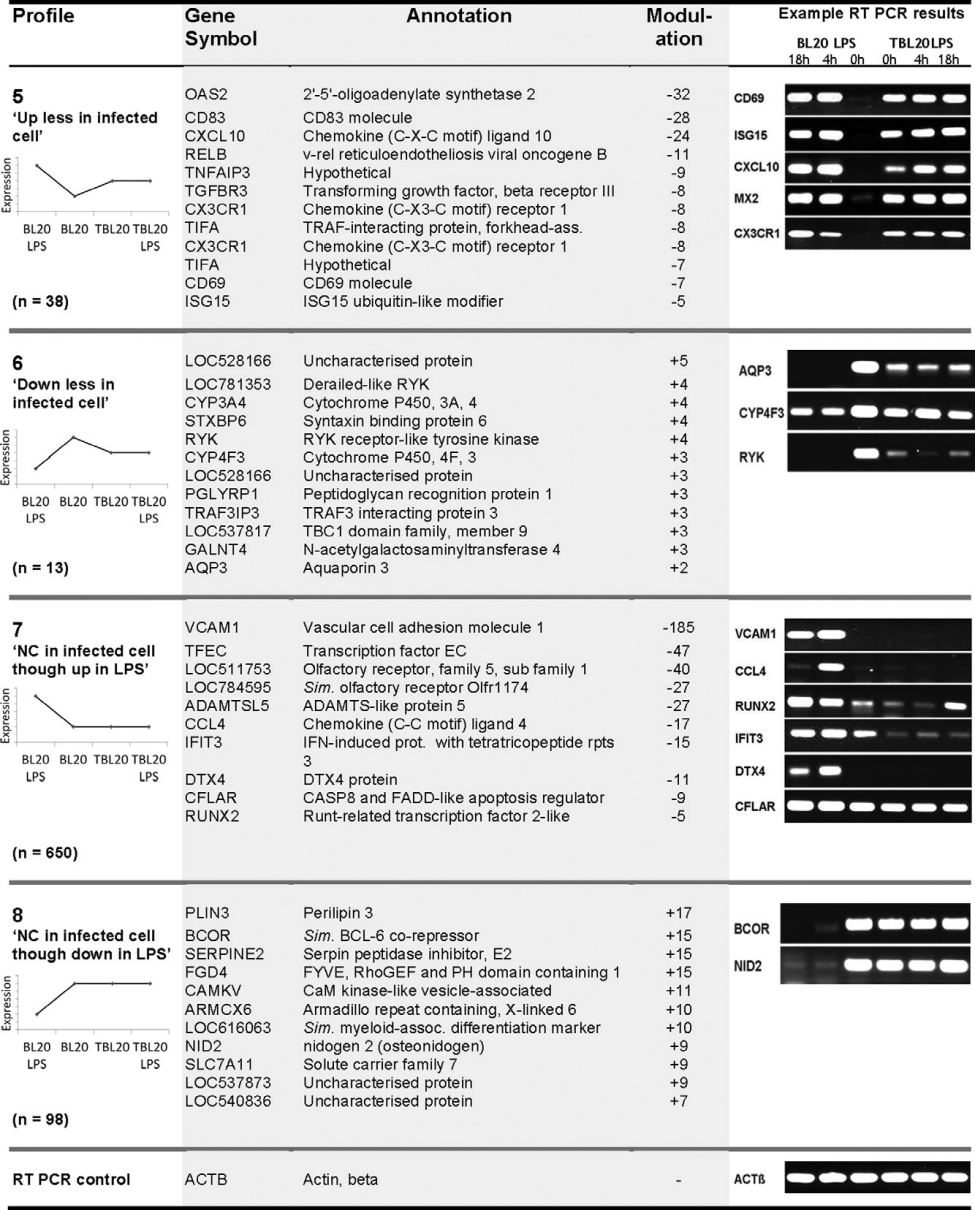
Expression profiles of BL20-LPS-response genes showing a modulated response in *Theileria*-infected cells. The different gene expression response profiles for TBL20 and BL20 cells obtained before and following LPS treatment are shown diagrammatically to the left, together with the total number of genes in that profile. Denoted genes (middle) represent top-ranking/selected (based on predicted function) differentially expressed genes within the profile. Semi-quantitative RT-PCR validation for a panel of the selected genes is shown on the right.

Profiles 1 and 2 represent an enhancement of gene expression in infected cells compared with the modulation detected for LPS stimulated BL20 (elevation of an LPS upregulated gene, profile 1; stronger repression of an LPS downregulated gene, profile 2). These patterns of modulation could reflect a quantitative difference in activation status in the infected cells. The level of NF-κB reporter activity, for example, was found to be significantly higher in TBL20 compared with BL20-LPS.

Profile 1 comprised 32 genes and for many of these genes the amplitude of enhanced, expression associated with infected cell was considerable (Fig 3 and Table S1). Thus, the genes encoding chemokine (C motif) ligand 1, MMP13 and an IL2RB-like protein were elevated 42-, 54- and 11-fold respectively in TBL20 relative to the maximal response obtained for LPS stimulated BL20 cells. Of the 14 BW720c-sensitive genes identified (Table S1), 10 (71.4%) responded in an expected manner, i.e. expression was significantly reduced by treatment over 48 h. Semi-quantitative RT-PCR was performed for eight genes, each of which validated the microarray profile (Fig 3), the IL2 receptor B-like gene (Herrmann *et al*., 3); the gene encoding the MEIS3 transcription factor, for example, showed a clear elevation in PCR product in LPS-stimulated uninfected BL20 cells but a considerably higher level in infected TBL20, with no obvious detectable alteration following LPS stimulation of TBL20. Further validation of the profile was obtained by qRT-PCR for *cortactin* and *MEIS3* (see Fig. S2, panels 1A and 1B) where the results agreed with the microarray data. qRT-PCR for *MEIS3* showed a 5.98-fold difference (log_2_) in TBL20 relative to BL20-LPS, compared with a difference of 3.1-fold (log_2_) using the array data, while qRT-PCR for *cortactin* calculated the difference to be 1.31-fold (log_2_) compared with 1.18-fold (log_2_) from the array data. The two functional categories that showed the most significant enrichment for profile 1 genes (Fig. S3) were ‘control of cellular movement’ and ‘cell-to-cell signalling’, and there was also an enrichment of genes associated with inflammation in this profile. In addition, a number of genes in profile 1 have predicted functions that can be linked to the phenotype of *Theileria*-transformed leucocytes. Thus, elevated expression was detected for a matrix metallopeptidase gene (Baylis *et al*.), genes encoding cellular adhesion molecules, *ICAM1* (Jensen *et al*., 2008) and *selectin L* (SELL), and genes that regulate the cytoskeleton, *cortactin* (CTTN) (Baumgartner, 2011). It can be postulated that genes captured in profile 1 are expressed at an elevated level in TBL20 because they are advantageous for establishment and or survival of the infected cell.

Profile 2 is the reciprocal of profile 1 and represents 180 genes whose expression is repressed in BL20-LPS but super-repressed in TBL20. Twenty-four of these genes showed major repression of expression associated with the infected cell (> 10-fold). Of the 83 genes sensitive to BW720c, 99.8% responded in the expected manner, indicating repression requires a viable parasite. Nine genes of this profile were validated by RT-PCR and agreed with the array profile (Fig 3). qRT-PCR validation was performed for *TLR4* and *MYL4* and showed good correlation with the microarray results (Fig. S2, panels 5A and 5B). A number of genes encoding transcription factors were present in the data set including LEF-1 (down 15-fold) and LMO2 (down 11-fold), both of which are strongly associated with leukaemia (Curtis and McCormack, 2010; Gutierrez *et al*., 2010). Moreover, genes encoding membrane proteins/receptors were particularly prominent in the data set. Among 13 receptor genes that showed stronger downregulation in TBL20 compared with BL20-LPS were those encoding the IGFR1 receptor (down 85-fold in TBL20 relative to BL20-LPS), the Toll-like receptor 4 (TLR4: down 44-fold), the G-protein-coupled receptor (CCR6: down 23-fold), S1PR1 (down 6-fold) and the thrombospondin receptor (down 6-fold). Thus, data from this profile strongly suggests that genes encoding molecules located to the surface of the infected cell are repressed and could influence interaction of the infected cell with a range of ligands, inflammatory mediators and cells of the bovine immune system. It can be predicted from the super-repression profile that high-level expression of the identified genes is most likely to be detrimental to the infected cell.

TLR4 is the major cellular receptor that transduces the LPS activation signal (Chow *et al*., 1999) and significant reduction in expression (61.4-fold between BL20 versus TBL20) could play a role in the unresponsiveness of *Theileria*-infected cells to LPS identified in this and previous studies. The gene encoding the LPS ligand CD14 was also expressed at a very low level in both uninfected BL20 and TBL20 cells. It has been demonstrated, however, that a lack of CD14 expression in bovine cells does not preclude recognition of LPS stimulation, possibly via the presence of soluble CD14 present in fetal calf serum (Sauter *et al*., 2007).

**Figure 5:**
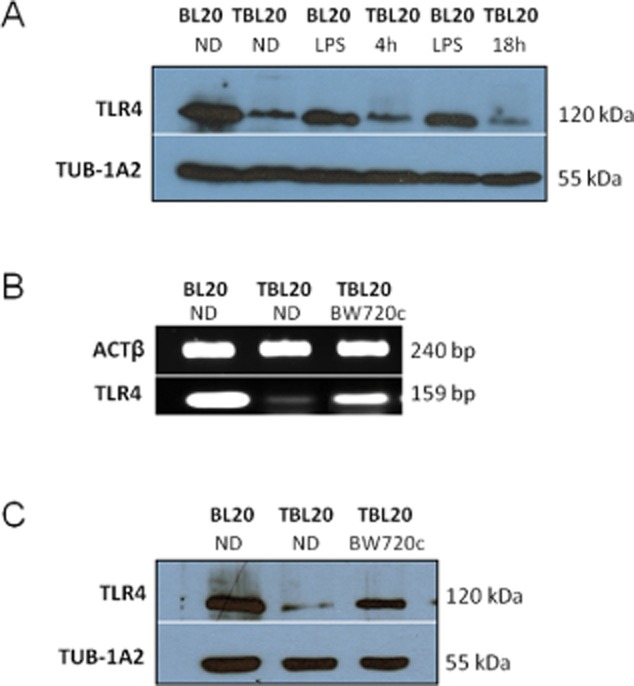
A. Immunoblot analysis of TLR4 protein expression following stimulation of BL20 and TBL20 cells with LPS. Expression of TLR4 protein was detected by immunoblot analysis of total protein extracts derived from uninfected and *Theileria*-infected cell cultures stimulated with either LPS for 4 and 18 h or no drug DMSO (1 μl ml^−1^) vehicle control (ND). Detection of β-tubulin was used as a constitutive control to ensure equal loading of extract. B. RT-PCR analysis of *TLR4* expression following treatment of TBL20 cells with BW720c. Expression of TLR4 at the mRNA level was assessed by semi-quantitative RT-PCR using RNA isolated from BL20 and TBL20 cells cultured with no drug (ND) or TBL20 cells cultured with BW720c at 50 ng ml^−1^ for 48 h. Primers specific for the β-actin gene (ACT β) were used as a control for constitutive expression. C. Immunoblot analysis of TLR4 protein expression following treatment of TBL20 cells with BW720c. Expression of TLR4 protein was assessed by immunoblot analysis of total protein extracts of BL20 and TBL20 cells cultured with no drug (ND) or TBL20 cells cultured with BW720c at 50 ng ml^−1^ for 48 h. Detection of β-tubulin was used as a constitutive control to ensure equal loading of extract.

Super-repression of TLR4 was validated at the protein level: immunoblotting with a specific anti-TLR4 antibody clearly demonstrated lower levels of TLR4 protein in TBL20 extracts relative to uninfected BL20 extracts from control or LPS stimulated cells (see Fig 5A). In addition, both RT-PCR and immunoblotting showed that killing the parasite with BW720c considerably elevated the level of TLR4 mRNA and protein (Fig 5B and C). Thus, repression of TLR4 by the infected cell is dependent on a viable parasite. The TLR4 signal transduction pathway plays a major role in activation of NF-κB following recognition of pathogen-associated molecular patterns (PAMPs). Simplistically, repression of this pathway may not be in the interest of the *Theileria*-infected cell, since it has been demonstrated that constitutive activation of NF-κB is required for its survival (Heussler *et al*., 1999). Parasite-dependent constitutive activation of NF-κB, however, is considered to be independent of extrinsic signalling, as the parasite promotes aggregation of active IKK complexes at the macroschizont surface (Heussler *et al*., 2002). Whether the repression of TLR4 gene expression provides an advantage to the infected cell is not clear but it could reduce the level of activation of NF-κB that occurs via extrinsic inflammatory stimuli. This would allow the level of NF-κB activation to be primarily set by the parasite-dependent IKK signalosome pathway. A block in TLR signalling has been reported for *Toxoplasma gondii*. It has been proposed that this allows prevention of pro-inflammatory responses triggered by *T. gondii*-derived TLR ligands, or gut flora, or reflects a general non-responsiveness to pro-inflammatory stimulation (reviewed by Leng *et al*., 2009). In addition, mammalian p38 MAPKs which are activated by LPS and play a major role in apoptosis (Guha and Mackman, 2001; Zhou *et al*., 2007) are known to have low or undetectable activity in *Theileria*-infected cells (Botteron and Dobbelaere, 1998).

While modulation of expression associated with profiles 1 and 2 may relate to the level of transcription factor activation, this is unlikely to be the case for profiles 3 and 4. In these profiles TBL20 was associated with a gene expression change opposite to that observed for BL20-LPS, and it can be postulated that it is of benefit to the infected cell to strongly repress (profile 3) or elevate (profile 4) expression of these genes. Profile 3 represents 154 genes elevated by LPS stimulation of uninfected cells that are significantly repressed in infected TBL20 (Fig 3). Almost all of the 63 genes that were sensitive to BW720c treatment in this profile responded in a reversible manner (i.e. 98.4% showed elevated expression with BW720c treatment), indicating active repression by a viable parasite. Many genes (*n* = 84) provided evidence of a repression corresponding to a fold change of greater than ten. Seven genes from profile 3 were validated by semi-quantitative RT-PCR (Fig 3). The gene encoding MPEG1, for example, showed an elevation in PCR product in LPS-stimulated uninfected BL20 cells while the level was clearly lower in infected TBL20 relative to BL20 cells. qRT-PCR performed for the *BOLA-DYA* and *BIRC3* genes (Fig. S2, panels 2A and 2B) validated the microarray profile. Profile 3 showed enrichment for genes predicted to encode proteins involved in cell-to-cell signalling, cell morphology, cellular development and cellular growth and proliferation. A significant number of genes were associated with cancer and apoptosis. Genes in profile 3 included those encoding macrophage expressed MPEG1, the inflammatory cytokine IL15, and the major histocompatibility complex class II DY alpha. Repression of these genes may be advantageous to the parasite-infected cell by altering the differentiation status of the host cell or manipulating the immune response.

Profile 4 is the reciprocal of profile 3 and represents 55 genes that were repressed by LPS treatment of uninfected BL20 cells but were elevated in infected TBL20 cells. The level of modulation associated with this profile was considerable. For example, the expression of genes encoding AHNAK, CMRF-35 and SLA were amplified 235-fold, 99-fold and 45-fold respectively. A further 33 genes were elevated more than 10-fold. Interestingly, a large proportion (61.5%) of the 13 BW720c-sensitive genes responded in an unexpected manner, i.e. they were upregulated by BW720c. Microarray expression data for profile 4 was validated by RT-PCR using five genes. qRT-PCR validation was performed for *ANGPT4* and *NDRG1* and showed good correlation with the microarray (see Fig. S2, panels 6A and 6B). Of the 51 genes that could be mapped onto the pathway analysis framework, 21 showed an association with cancer. The profile 4 data set showed enrichment for genes encoding proteins that function in ‘cell movement’, ‘cell death’ and ‘cell growth and proliferation’. Gene ontology indicated that a number of genes (*ANGPT4*, *NDRG1*, *PAK1*, *PI3KR6*, *SLA*, *SPARC*) operate in signal transduction events. Overall, this profile suggests that the infected cell is strongly associated with upregulation of genes linked to neoplasia.

Profiles 5 and 6 both provide evidence that infected TBL20 cells attenuate the response of uninfected BL20 cells to LPS. Thus, profile 5 represents 38 genes with expression levels elevated in *Theileria*-infected cells, but to a lesser extent than in LPS stimulated BL20 cells. An example of this profile is the gene for interferon-induced protein 15, *ISG15*, elevated 121-fold in BL20-LPS but only 26-fold by infection TBL20 (relative to BL20) and no change in TBL20-LPS. These kinetics mirror reduced expression associated with *Theileria*-infected cells previously observed by Northern blotting (Oura *et al*., 2006). Five genes of the profile were validated by semi-quantitative RT-PCR (Fig 4). qRT-PCR performed for the *CD69* and *ISG15* genes confirmed the microarray data (Fig. S2, panels 3A and 3B). Functional analysis showed enrichment for genes encoding proteins involved in protein modification, the antimicrobial response, the inflammatory response, cellular defence and the innate immune response (e.g. *ISG15*). Genes encoding transcription factors activated by *Theileria* infection such as *NF-κB2*, *NF-κBIZ* and *Jun* were also identified. Altered expression of genes in profile 5 could indicate a limited upregulation of a beneficial gene that does not occur to the same degree as in BL20-LPS (e.g. the Jun proto-oncogene). However, it may be more likely that high-level expression of cellular defence genes, such as *ISG15* (Oura *et al*., 2006) that occur via activation of the cell, are repressed by infection as they are potentially detrimental. The enrichment in genes associated with the interferon/innate immune/cellular defence response, e.g. *OAS2*, *ISG15*, *MX2*, *IRF5* and *IL7* (Moller *et al*., 1996; Jin *et al*., 1999; Urosevic, 2003; Pitha-Rowe and Pitha, 2007; Bin *et al*., 2011), supports this possibility. Furthermore, 72% of the 18 BW720c-sensitive genes in profile 5 showed elevated expression upon drug treatment, indicating active repression by the viable parasite.

Profile 6 was the reciprocal of profile 5 and represented genes where expression was repressed with LPS treatment of BL20 but repressed to a lesser extent in parasite infected TBL20 cells. This profile represented the smallest number of genes (*n* = 13), and four out of the five BW720c-sensitive genes were elevated upon treatment. The array profile was validated for three genes by RT-PCR and by qRT-PCR for *CYP4F3* and *RYK* (Fig. S2, panels 7A and 7B). Pathway analysis of the data set for this profile showed enrichment for genes predicted to function in metabolism or small molecule biochemistry.

Profiles 7 and 8 represent no change in infected TBL20 cells compared with an altered response identified for BL20-LPS (up or down). Profile 7 represents 650 genes elevated by LPS stimulation of BL20 that showed no change on comparison of BL20 with TBL20 and was the largest obtained by the study. Only a small number of these genes (*n* = 6) were sensitive to BW720c, supporting a mechanism of non-responsiveness, rather than repression of an activation event associated with the infected cell. In some cases, however, postulation of a lack of response mechanism may be over-simplistic. *VCAM1*, for example, is a classical NF-κB-dependent gene (Luo *et al*., 2010) and it is conceivable that infection of the host cell is associated with a more direct repression of *VCAM1* expression. The microarray profile was successfully validated by RT-PCR on six candidate genes (see Fig 4) with the exception of *Runx2* where an increase in *Runx2* PCR product was detected at the TBL20 LPS 18 h time point. qRT-PCR validation indicated a 13.9-fold (log_2_) lower expression level for *VCAM1* in TBL20 versus BL20-LPS (Fig. S2, panels 4A), compared with a difference of 7.4-fold (log_2_) generated by the array data; while for *Runx2,* qRT-PCR showed a 5.1-fold (log_2_) reduction in expression values between TBL20 and BL20-LPS (Fig. S2, panels 4B), compared with a difference of 2.5-fold (log_2_) from the array data. Pathway analysis of this profile identified enrichment for genes within the categories ‘inflammatory response’ and ‘inflammatory disease’, and it was of interest that ‘activation of IRF by cytosolic pattern recognition receptors’ and ‘interferon signalling’ were identified as the most significantly enriched canonical pathways.

Profile 8 is the reciprocal of profile 7 and represents 98 genes where expression was downregulated by LPS treatment of BL20 but showed no change associated with infection of BL20 (BL20 versus TBL20). Like profile 7, genes sensitive to BW720c treatment were under-represented in this profile with only a single one present. The microarray data were validated by RT-PCR for three genes and by qRT-PCR for *BCOR* and *RGS1* (Fig 4 and Fig. S2, panels 8A and 8B). Pathway analysis showed this profile is enriched for genes predicted to function in cell signalling, small molecule biochemistry and metabolism. This suggests that the infected cell maintains metabolic processes required for cellular proliferation (as also indicated for profile 6).

Together, the eight profiles identified above provide evidence that the parasite-infected cell is associated with significant modulation of the response of uninfected BL20 cells to activation with LPS. A sizeable proportion of these alterations to host cell gene expression are under control of a viable parasite, as they are altered by treatment with BW720c. However, the majority of the identified infected cell modulations to gene expression did not respond to drug treatment. While this may be influenced by the shorter time periods used for drug treatment relative to previous studies (Oura *et al*., 2006; von Schubert *et al*., 2010), it seems likely that a large number of the alterations to host cell gene expression are genuinely non-responsive. Such alterations could be derived from epigenetic events that occurred during establishment or passage of the BL20 and TBL20 cell lines. These could be disparate events that are independent of infection, although it is also possible that epigenetic events are influenced by the parasite to generate infected cell-associated alterations that cannot be altered by drug treatment over 48 h.

### Pathway analysis of the infected cell-modulated data set can predict phenotypes displayed by *T. annulata-*infected leucocytes

**Figure 6:**
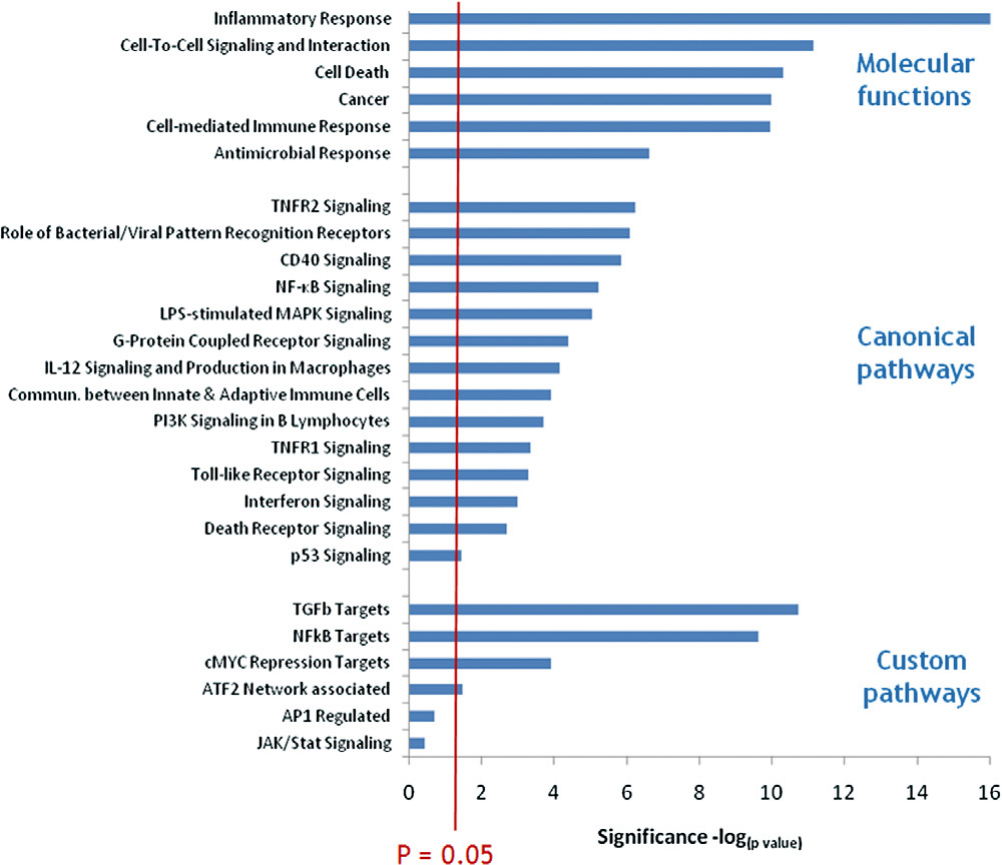
Enrichment analysis of the infected cell-modulated gene list. The infected cell-modulated gene list (*n* = 1214) was analysed for evidence of enrichment with respect to two areas within the pathway analysis database, i.e. molecular function and canonical pathways. The gene list was also analysed for enrichment with respect to a number of denoted custom pathways. Top-ranking categories are presented together with the significance of the enrichment [−log(*P*-value)].

Derivation of the eight profiles of genes displaying altered expression levels in infected TBL20 cells relative to LPS-stimulated uninfected BL20, provides evidence that *T. annulata*-infected cells alter the gene expression outcome associated with an activation event, i.e. the response to LPS. To gain information on the potential impact of these alterations, the entire list of 1214 genes was analysed for enrichment in specific molecular functions and canonical pathways. A large number of categories were identified for the 976 genes mapped onto the pathway analysis framework but it was clear that the data set was very significantly enriched for genes predicted to function in the inflammatory response, cell death, cancer and the antimicrobial response (Fig 6).

To make the output of the pathway analysis more predictive, further enrichment analysis was undertaken, noting whether each molecule promoted (pro) or inhibited (anti) a particular molecular function. The proportion of genes designated as ‘pro’ or ‘anti’ for each functional category was then examined in relation to whether their expression is repressed or enhanced by infected TBL20, in order to infer how the infection-associated modulation could influence a functional outcome. Interestingly, functional categories highlighted by this analysis (Table 1) predominantly show an association with a phenotype that is known or could be reasonably predicted for the infected cell. For example, in the ‘metastasis of cells’ subcategory, the observed trend indicated repression of anti-metastatic genes and elevated expression of pro-metastatic genes, implying that the parasite-infected leucocyte promotes metastasis. Genes classified within ‘cancer’ and ‘tumorigenesis’ were associated with a bias towards pro function in genes showing enhanced expression associated with the infected cell, whereas a bias towards anti-function was associated with repression by infected TBL20 within the categories ‘disease of tumour’ and ‘growth of tumour’. Additional categories include the inflammatory response and cell movement. In the inflammatory response category, the data indicated that genes promoting an ‘anti-microbial/anti-viral’ response or ‘inflammation’ are associated with repression by parasite-infected cells, while for ‘activation of leucocytes’ and ‘activation of lymphocytes’ a trend towards elevated expression of positive effector genes was obtained. Similar results were observed for cell movement, one subcategory ‘invasion of eukaryotic cells’ indicating elevated expression of genes pro this function, whereas the next three subcategories all indicated a trend towards repression of genes pro movement, including those categorized in ‘influx of leucocytes’ and ‘chemoattraction of leucocytes’. One possible explanation for these results is that they reflect a requirement for activation and dissemination of the infected leucocyte together with dysregulation/evasion of the immune response.

**Table 1:** Functional classification of the infected cell-modulated gene set

	Functional classification	Repressed in parasite-infected cell				Enhanced in parasite-infected cell					
No. genes (pro/anti)	*P-*value	No. genes (pro/anti)	*P-*value
1	Inflammatory response		
Activation of leucocytes	46 (27/9)	2.7 × 10^−6^	13 (9/1)	3.8 × 10^−3^
Antimicrobial response	19 (3/1)	4.9 × 10^−5^	–	–
Activation of lymphocytes	31 (19/7)	9.5 × 10^−5^	9 (5/1)	1.2 × 10^−2^
Antiviral response	13 (3/1)	9.6 × 10^−5^	2 (0/0)	1.2 × 10^−2^
Inflammation	30 (12/3)	7.2 × 10^−4^	12 (3/2)	6.5 × 10^−4^
2	Cell death		
Apoptosis of tumour cell line	72 (34/22)	2.3 × 10^−6^	20 (12/7)	3.1 × 10^−3^
Apoptosis	134 (70/53)	2.1 × 10^−5^	42 (24/19)	1.1 × 10^−4^
Cell death of tumour cell line	78 (38/28)	2.1 × 10^−5^	24 (13/9)	9.4 × 10^−4^
Cell death	151 (75/62)	6.6 × 10^−5^	49 (26/22)	3.2 × 10^−5^
3	Cellular movement		
Infiltration of cells	29 (10/6)	4.2 × 10^−5^	–	–
Invasion of eukaryotic cells	–	–	14 (10/3)	5.9 × 10^−4^
Migration of antigen presenting cells	14 (7/1)	1.6 × 10^−4^	–	–
Influx of leucocytes	7 (4/1)	2.0 × 10^−3^	–	–
Chemoattraction of leucocytes	6 (6/0)	4.2 × 10^−3^	–	–
4	Cancer		
Metastasis of cells	10 (2/6)	3.2 × 10^−3^	13 (3/1)	2.3 × 10^−3^
Tumorigenesis	179 (26/25)	1.7 × 10^−3^	60 (6/2)	1.7 × 10^−5^
Cancer	173 (16/13)	7.6 × 10^−5^	56 (3/1)	1.6 × 10^−5^
Disease of tumour	18 (2/4)	7.3 × 10^−4^	–	–
Growth of tumour	20 (2/9)	1.2 × 10^−3^	–	–
Tumour	–	–	43 (4/2)	1.1 × 10^−4^
5	Cell assembly and organization		
Formation of actin filaments	–	–	8 (3/1)	1.3 × 10^−3^
Formation of actin cytoskeleton	4 (0/0)	1.3 × 10^−3^	4 (2/0)	1.1 × 10^−2^
Formation of filaments	–	–	9 (5/1)	4.1 × 10^−3^
Formation of cellular protrusions	–	–	10 (4/0)	2.7 × 10^−3^

The ability of the pathway analysis to predict phenotypes displayed by the *Theileria*-infected cell suggests that the infected cell-modulated gene list could be mined to highlight candidate novel pathways and genes that operate in its establishment and maintenance. Examples of several candidate genes and categories of interest are given below.

Sixty genes (24%) were placed in the cancer category (*P*-value, 1.56 × 10^−5^), including pro-cancer genes *MMP13*, *SPARC*, *NDRG1*, *PAK1*, *ICAM1*, *ANGPT4*, *IL2RB*-like, *RGS1*, *SELL*, *SERPINE2*, *CCR7*, *CTTN*, *LTB4R*, *SLC2A1* and *TP73* which are expressed at significantly higher levels in parasite-infected TBL20 relative to BL20-LPS. Intercellular adhesion molecule-1 (ICAM1) is elevated sixfold in TBL20 cells relative to BL20-LPS (profile 1), and elevated 23-fold relative to uninfected BL20. ICAM1 is a trans-membrane glycoprotein and is encoded by a classical NF-κB target gene (Huang *et al*., 2004; Lin *et al*., 2006). It plays an important role in the immune response, but has also been implicated in cancer metastasis and linked to tumour prognosis and progression (Huang *et al*., 2004; Lin *et al*., 2006). Interestingly, the level of ICAM1 mRNA was found to be significantly higher in infected monocytes derived from disease-susceptible Holstein cattle relative to resistant Sahiwal (Jensen *et al*., 2008). Such a difference could be generated by variability in the level of activated NF-κB in infected cells from different breeds, or a difference in a parasite-dependent mechanism that controls NF-κB target gene expression. Further investigations are required to characterize the role of ICAM1 expression in determining the virulence of the *T. annulata*-infected cell.

Secreted protein acidic and rich in cysteine (SPARC) is a matricellular protein that influences cell–matrix interactions and has been reported to have multiple roles in macrophage function, inflammatory processes, cell morphology, inhibition of cell cycle progression and synthesis of the extracellular matrix (Rempel *et al*., 2001). Many cancer types exhibit increased SPARC levels upon invasion or metastasis (Rempel *et al*., 2001; Podhajcer *et al*., 2008) and upregulation of SPARC expression (53-fold) in infected in TBL20 cells (profile 4) may play an important role in establishing the infected cell phenotype. For example, recent studies have described a connection between SPARC and the p53 tumour suppressor (Fenouille *et al*., 2011). Essentially, SPARC acts as an anti-stress factor by mediating degradation of p53 through AKT-mediated MDM2 phosphorylation to promote melanoma cell survival. Array data indicate that the level of p53 mRNA is high in *Theileria*-infected leucocytes, but protein levels are virtually undetectable (B. Shiels and S. McKellar, unpublished). The PI3K/AKT pathway is known to be constitutively activated in the *Theileria*-infected leucocyte (Baumgartner *et al*., 2000; Heussler *et al*., 2001). Investigation of a mechanism that may operate to promote constitutive degradation of p53 is required.

The category ‘cellular assembly and organization’ was predicted to show an association with the infected cell phenotype, as a bias towards enhanced expression of pro genes within a number of subcategories was identified. Subcategories included ‘formation of actin filaments’ and ‘formation of actin cytoskeleton’ and predict that infection of the host cell is associated with manipulation of cell shape and the actin cytoskeleton. Upregulated genes included *CTTN, SDC4, ICAM1, PAK1, ELN, SPARC, SORBS1, TNS1* and *F2R*. *Theileria* infection is known to alter the morphology of the host cell (Schmuckli-Maurer *et al*., 2010; Baumgartner, 2011) and it has been postulated that organization of the actin cytoskeleton has an impact on parasite-dependent activation of NF-κB via the IKK signalosome (Schmuckli-Maurer *et al*., 2010). Recent studies have shown that parasite-dependent polarization of actin-based structures such as lamellipodia and podosomes are critical for both attachment to substrates and motility (Baumgartner, 2011). Molecules thought to function in regulation of these structures include the Src kinase, Hck, the Rho kinase, ROCK and cortactin. Elevated expression of the cortactin (CTTN) and Hck (haemopoietic cell kinase) genes in infected TBL20 cells support a role in regulation of infected cell shape.

The gene, *PAK1*, encoding the p21 (Cdc42/Rac)-activated kinase 1 (member of a family of serine/threonine protein kinases) is significantly upregulated (> 22-fold) in *Theileria-*infected TBL20 compared with BL20-LPS (profile 4). PAK1 has been reported to have a number of functions (e.g. cell survival, motility, proliferation) and is implicated in transformation and tumour progression (Huynh *et al*., 2010). PAK1 is a key molecule in the regulation of the actin and microtubule cytoskeleton (reviewed by Bokoch, 2003), plays a crucial role in formation of podosomes and membrane ruffles, can phosphorylate cortactin to regulate the dynamics of branched actin filaments (Webb *et al*., 2006) and enhances cell migration and proliferation via AKT (Huynh *et al*., 2010). Intriguingly, PAK1 has been reported to operate in pathogen-dependent activation of NF-κB via NIK (Neumann *et al*., 2006) and inhibition of NIK reduces activation of NF-κB in *Theileria*-infected cells by 25% (Heussler *et al*., 2002). Further studies investigating the role PAK1 may play in establishing the transformed phenotype of the *Theileria*-infected cell are indicated.

In contrast to the categories and genes highlighted above, an expected bias towards upregulation of anti-apoptotic genes together with downregulation of pro-apoptotic genes could not be identified, although the present analysis framework may not have had the power to resolve such a phenomenon. This may be due to unequal numbers of ‘pro’ and ‘anti’ apoptosis genes denoted in the database together with the fact that genes functioning in apoptosis commonly switch from a ‘pro’ to ‘anti’ function depending on cellular context (Fannjiang *et al*., 2003; Hess *et al*., 2004) and such subtleties will not be reflected in the database. Moreover, the probability of progressing towards apoptosis may be associated with qualitative differences, such as phosphorylation status, or the stoichiometry of a number of key factors and this would not have been revealed by the analysis.

### *Theileria*-infected TBL20 cells modulate expression outcomes associated with activation of NF-κB and other infection-activated transcription factors

**Figure 7:**
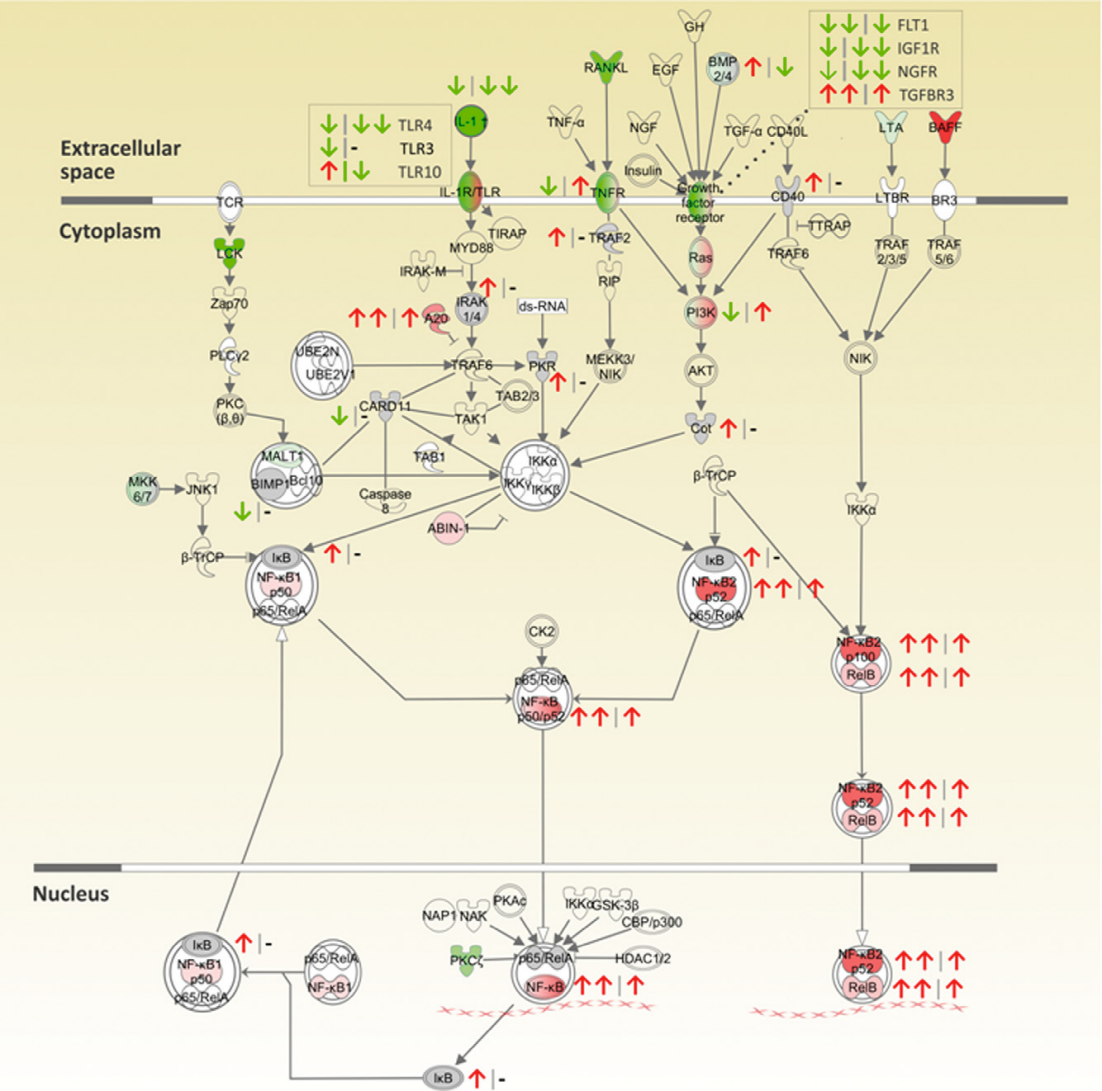
NF-κB signalling pathway. The genes which are coloured are differentially expressed in TBL20 compared with BL20; red = up in TBL, green = down in TBL. The arrows are beside genes present in the infected cell-modulated list (1214). For example, 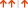 means super-up in BL20-LPS|up in TBL20 compared with BL20. The data for IL-1R/TLR and growth factor receptor are exploded in two boxes.

Stimulation of cells with inflammatory mediators such as LPS, and infection by *T. annulata*, is known to result in activation of transcription factors via a number of signalling pathways. Therefore, the infected cell-modulated data set of 1214 genes was analysed with respect to canonical pathways, including those involved in signalling. Unsurprisingly, the results highlighted modulation of signalling events associated with the inflammatory response. Significant pathways include TNFR2, pattern recognition receptors, CD40, NF-κB, PI3K, LPS-stimulated MAPK and interferon signalling (Fig 6). An event common among many of these pathways is the activation of NF-κB. As predicted from the findings of Heussler *et al*. (2002), the microarray data generated little evidence for major repression of the NF-κB signalling pathway below the level of the activated signalosome (Fig 7); repressed expression of the genes encoding three IκBs (relative to LPS) would promote NF-κB activation but there is no evidence of a difference in expression of these IκB genes between non-stimulated BL20 and TBL20 cells. Upstream of the signalosome, repression of genes encoding receptors appears to be in operation and it can be concluded that, in general, infected TBL20 downregulate the ability to recognize extrinsic stimuli that induce activation of NF-κB. Exceptions to this observation are upregulation of the genes encoding the TNFR2 and TGFBR3 receptors. Both TNF and TGFβ signalling pathways have been associated with the *Theileria*-infected leucocyte (Guergnon *et al*., 2003; Chaussepied *et al*., 2010) and could potentially activate NF-κB via TRAF2 or PI3K. However, evidence for a role of the PIK3 pathway in activating NF-κB is conflicting (Baumgartner *et al*., 2000; Heussler *et al*., 2001), and the importance of a TNF autocrine loop is unclear (Guergnon *et al*., 2003). Furthermore, microarray data indicate that the gene encoding tumour necrosis factor alpha induced protein 3 (TNFAIP3/A20) that acts as an inhibitor of TNFR2-dependent NF-κB signalling (Charan *et al*., 2011) is upregulated in TBL20 relative to BL20. The importance of the PI3K pathway for proliferation of the infected leucocyte (Baumgartner *et al*., 2000; Heussler *et al*., 2001) is supported in the present study by evidence for upregulation of the genes encoding PI3K regulatory subunits 5 and 6 relative to repression by the LPS response.

Using a series of custom pathways, the infection-modulated data set was also screened for enrichment of target genes of transcription factors activated by the parasite-infected cell and associated with the inflammatory response. As shown in Fig 6, significant enrichment of NF-κB targets and cMyc repression targets was observed, together with a particular enrichment of genes regulated in response to stimulation by TGFβ. This provides evidence that the *Theileria*-infected leucocyte significantly alters the outcome of cellular activation by modulating the expression of target genes regulated by constitutively activated transcription factors.

**Figure 8:**
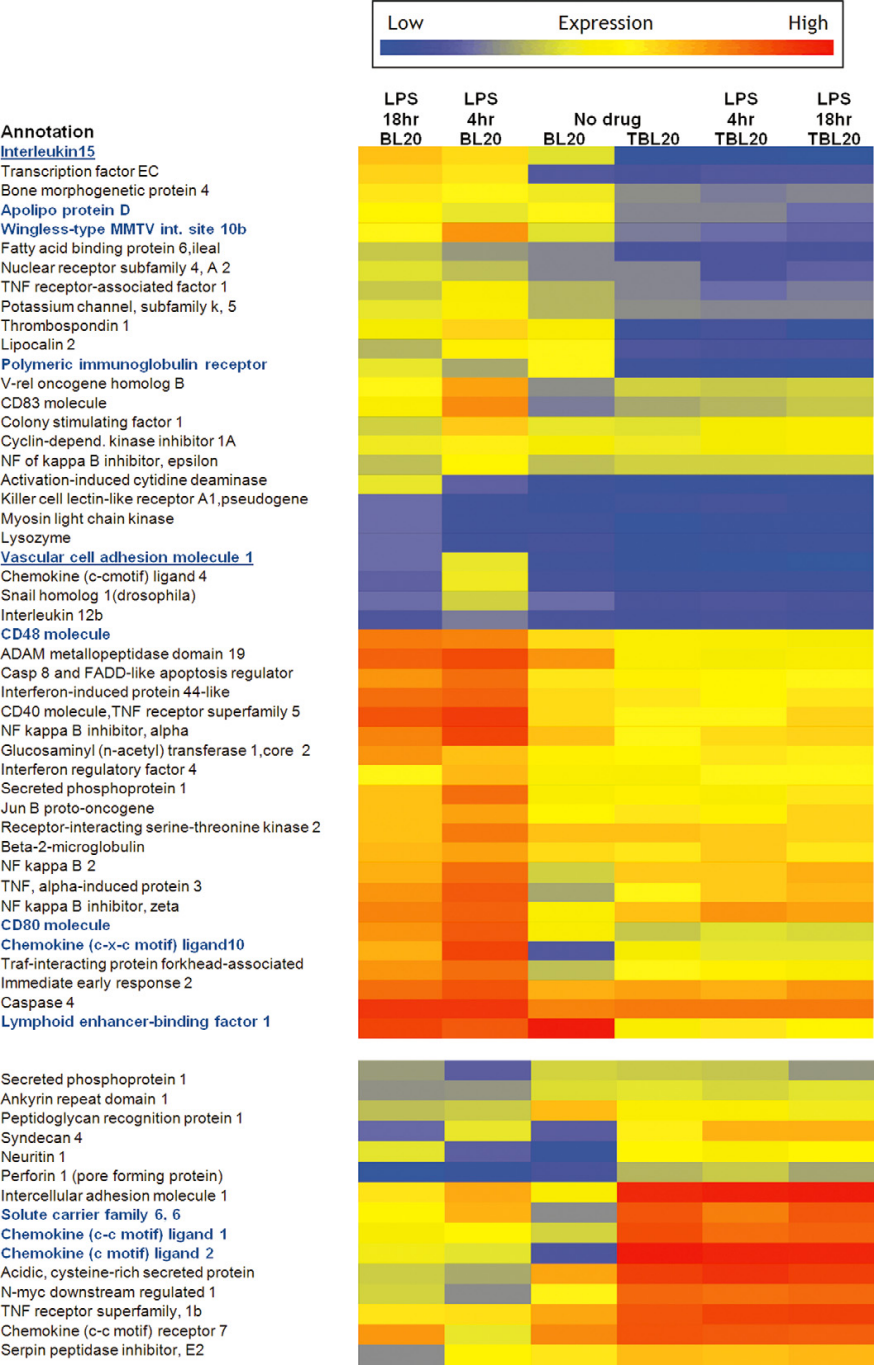
Hierarchical clustering of NF-κB response genes. The NF-κB targets genes identified in the infected cell-modulated gene list were selected for hierarchical clustering analysis and the results are presented as a heat map. Genes highlighted in bold show altered array expression following BW720c treatment for 48 h to kill the parasite. Underlined genes show altered RT-PCR expression following BW720c treatment for 72 h.

Investigation of NF-κB target genes revealed that the majority (46) were repressed in infected TBL20 cells relative to LPS stimulated uninfected cells (BL20-LPS), while 15 showed evidence of enhanced expression (Fig 8). For repressed NF-κB gene targets, enrichment was observed for pro-inflammatory response (e.g. CXCL10, *CCL4* and *IL15*), immunoreceptors and pro-differentiation of leucocytes. CXCL10 is an angiostatic chemokine that represses the ability of Burkitt's lymphoma cells to produce tumours in nude mice (Sgadari *et al*., 1996). Overexpression of CXCL10 has been implicated in inhibition of the proliferation process in a variety of cell types. IL15 is a major pro-inflammatory cytokine involved in enhancing cytotoxic CD8 T cell and NK cell activity and has therapeutic indications against cancer (reviewed by Steel *et al*., 2011). Based on the proposed role of CD8 T cells and NK cells in the immune response against *T. annulata* (reviewed in Preston *et al*., 1999), major downregulation of IL15 by the parasite-infected cell (i.e. 100-fold less in TBL20 compared with BL20 LPS) could be predicted to be beneficial to its establishment. Repressed NF-κB target genes also included regulators of apoptosis and genes involved in regulating the NF-κB mediated inflammatory response, i.e. TRAF1 (Henkler *et al*., 2003), *CFLAR*/FLIP (Zaitseva *et al*., 2011) and *CASP4* (Lakshmanan and Porter, 2007; Mao *et al*., 2010).

The number of infected cell-associated upregulated NF-κB target genes (Fig 8) was too small for enrichment analysis, but did include some interesting members, such as genes encoding the pro-metastatic chemokine receptor *CCR7* (Muller *et al*., 2001), *ICAM1*, *SPARC* (see above) and the N-myc downstream regulated 1 (*NDRG1*).

Investigation of genes classified as repression targets of cMyc indicated that the majority, 7 out of 10, displayed an expression profile consistent with maintenance of repression in the infected cell (data not shown). This included two genes encoding inhibitors of cell cycle progression, *CDKN1A* and *CDKN2B* (Gartel and Shchors, 2003). In marked contrast, *NDRG1* (classified as regulated by NF-κB and cMYC repression) showed a significant elevation associated with the parasite-infected cell relative to repression in BL20-LPS (Fig 8). NDRG1 has been proposed to be a suppressor of tumour growth and an attenuator of NF-κB activation (Hosoi *et al*., 2009) and, therefore, elevation of expression by infection could be considered counter-intuitive. However, a role for NDRG1 at the spindle checkpoint of mitosis has been established, and NDRG1 is thought to be important for maintaining correct microtubule functioning (Kim *et al*., 2004) and spindle formation. Recent work has shown an intricate relationship between the *Theileria* schizont and host cell mitotic and central spindles, dependent on recruitment of host cell Polo-like kinase (von Schubert *et al*., 2010).

Evidence for modulation of TGFβ target genes was also obtained, although a uniform alteration of the predicted response to TGFβ stimulation could not be identified for TBL20 cell relative to BL20-LPS (data not shown). Thus, roughly equivalent numbers of target genes expected to be upregulated or downregulated by TGFβ were identified within the ‘infected cell-associated repressed’ gene list, with a similar finding for the ‘enhanced’ gene list. The data set of TGFβ target genes modulated by infected TBL20 was found to be enriched for genes placed in the categories of ‘cancer’, ‘cell death’, ‘cellular growth’ and ‘cellular development’. Of particular note was that TGFβ targets categorized as inflammatory response genes were generally associated with repression of expression by parasite-infected TBL20 relative to the BL20-LPS response. The lack of any association between a predicted TGFβ response and alteration of expression by the infected cell may be explained, in part, by the finding that expression of genes encoding TGFβ2 and TGFβ3 and the TGFβR3 receptor is slightly elevated in parasite-infected TBL20 cells, while TGFβ1 is slightly reduced. The TGFβ signalling pathway has been implicated as a determinant of pathogenicity and metastatic activity in tropical theileriosis: reduced TGFβ2 expression was measured for infected cells derived from an attenuated vaccine line and disease-resistant Sahiwal (*Bos indicus*) cattle (Chaussepied *et al*., 2010). Differential expression of TGFβ target genes was also identified, with elevated as well as repressed expression reported for the attenuated vaccine cell line. Thus, modulation of TGFβ signalling and the respective target genes appears to be a common event associated with *T. annulata*-infected leucocytes.

The results of this study provide evidence that the *T. annulata*-infected cell tightly controls the outcome of NF-κB activation. This modulation, in general, can be predicted to be beneficial to establishment of the infected cell. The data also indicates that the outcome of activating additional pathways/transcription factors known to be associated with *Theileria*-transformed leucocytes are modulated, e.g. TNF, TGFβ and cMyc. Thus, a major re-organization of networks that regulate gene expression changes associated with the inflammatory response and cancer is likely to be concomitant with transformation of the infected leucocyte.

Recent studies on the related apicomplexan parasite, *T. gondii* have identified parasite proteins that regulate inflammatory gene networks by activating transcription factors, including NF-κB (Saeij *et al*., 2007; Rosowski *et al*., 2011). Evidence has also been generated that parasite-dependent modification of histone H3 can prevent transcription factors binding to host-cell inflammatory gene promoters (Leng and Denkers, 2009). It has been proposed that such a mechanism could account for the large-scale downregulation of pro-inflammatory genes by *T. gondii* (Leng *et al*., 2009). While it is possible that *Theileria* parasites deploy similar mechanisms to manipulate gene expression on a wide scale, our results suggest additional alterations are involved. This is indicated by the four pairs of reciprocal modulated expression profiles (Figs and 4), the ability of infected cells to reverse gene expression changes induced by LPS and the non-responsive and non-reversible nature of a large number of the host cell gene expression changes when the parasite is killed by drug. Such changes could be generated by an irreversible reconfiguration of transcription factor/expression networks associated with differentiation events, as increasingly proposed for stem cell systems (Chickarmane *et al*., 2009; Wang *et al*., 2010). In addition, deployment by *T. annulata* of DNA-binding proteins to the host cell nucleus that show similarity to mammalian HMGA proteins could be involved (Swan *et al*., 1999). HMGAs act as hubs of nuclear function and play a major role in organization of gene expression networks associated with inflammation and cancer (reviewed in Cleynen and Van de Ven, 2008). Given the propensity of *Theileria* parasites to control gene expression networks associated with inflammatory disease and cancer, further study of the mechanisms deployed and the outcome they promote is warranted.

## Experimental procedures

### Cell culture and LPS stimulation

Low-passage BL20, an uninfected bovine lymphosarcoma cell line (Olobo and Black, 1989), and TBL20, a *T. annulata* (Ankara strain) infected BL20 cell line (Shiels *et al*., 1986), were cultured in standard complete medium (Swan *et al*., 1999) except that heat-inactivated fetal bovine serum (FBS; Sigma) was used. Cultures were seeded with 2 × 10^5^ cells ml^−1^ and maintained by feeding with fresh medium every 2–3 days (Schmuckli-Maurer *et al*., 2010). Stimulation of cells was carried out for up to 48 h in six-well plates with 5 × 10^6^ cells well^−1^ in a total of 5 ml of medium, set up immediately prior to addition of 1 μg ml^−1^ LPS (Sigma: L2630). Optimum LPS concentration was determined by preliminary titration experiments which measured the minimum concentration of drug required to stimulate cells, based on NF-κB reporter activity, without causing rapid cytotoxicity (data not shown). LPS was dissolved in dimethyl-sulfoxide (DMSO) and control cells were incubated with DMSO alone. Treatment of TBL20 with BW720c was performed as described previously (Schmuckli-Maurer *et al*., 2010).

### Cell viability counts and apoptosis assay

Assessment of viable and non-viable cell numbers was performed using Trypan Blue exclusion counted using a haemocytometer; viable cells exclude the dye. Three replicate cultures of BL20 and TBL20 were seeded with 2.5 × 10^5^ cells ml^−1^ and incubated at 37°C for 18 h in the presence of LPS (1 μg ml^−1^) or DMSO (1 μl ml^−1^) as no drug control. Control experiments comparing cells culture in the presence or absence of DMSO showed no evidence of an effect on cell viability or activation of NF-κB (data not shown). Caspase-3/7 activities were measured using a Caspase-3/7 Assay kit (Promega) according to the manufacturer's protocol. Each assay was performed using 2.0 × 10^4^ cells from three replicate cultures per experimental condition at 18 h. Luminescence was measured using a TD −20/20 luminometer (Turner Designs). A no-cell control was carried out to account for baseline signal generated from fresh complete medium.

### NF-κB-dependent reporter assay (dual luciferase assay)

Estimation of NF-κB activity was performed by transient co-transfection of a reporter plasmid containing a promoter with an NF-κB3 recognition sequence linked to Firefly luciferase (NF-κB-LUC) and a Renilla luciferase plasmid (phRG-TK renilla), followed by sequential measurement of Firefly and Renilla luciferase activity, as previously described (Schmuckli-Maurer *et al*., 2010). Two micrograms of NF-κB3-Luc and 0.5 μg of phRG-TK Renilla plasmids were mixed with 4 × 10^6^ BL20 or TBL20 cells in 300 μl of RPMI-1640-2% FBS without antibiotics and electroporation performed using a Gene Pulser (Bio-Rad) set at 220V, 950 μF. After electroporation, cells were immediately added to 5 ml of pre-warmed complete medium in six-well plates. After 24 h recovery, LPS (1 μg ml^−1^) or DMSO were added for 4 h. Samples were harvested and the cell pellet washed with PBS (phosphate buffer saline).

A Dual Luciferase® Reporter Assay System (Promega) was used to assay activity, using the manufacturer's protocol. NF-κB promoter activity was calculated by normalizing firefly luciferase activity relative to the activity of constitutive Renilla luciferase. Each electroporation and luciferase assay was performed in quadruplicate, 20 μl of cell extract was used per assay.

### Statistical analysis

Statistical analysis and generation of graphs were performed using GraphPad Prism Software version 5.0 (La Jolla, CA). Data were expressed as mean ± standard error of the mean (SEM). Differences and significance between mean values were calculated using a two-tailed Student's *t*-test with *P*-values ≤ 0.05 considered to be statistically significant.

### Indirect immunofluorescence analysis

Indirect immunofluorescence analysis (IFA) was performed on uninfected BL20 and infected TBL20 cells cultured with LPS or in DMSO (1 μl ml^−1^), as no drug control, for 4 h. Cytospin slides were generated using poly l-lysine glass slides and IFA performed, as described previously (Schmuckli-Maurer *et al*., 2010). Primary antibodies, anti-IKKγ/NEMO (BD Biosciences; 611306), anti-NF-κB p65 (Santa Cruz; sc-372) and anti-NF-κB p65 (Santa Cruz; sc-8008), were used at 1:50 dilution; anti-rabbit IgG and anti-mouse IgG secondary antibodies, conjugated to Alexa 488 (Invitrogen; A-11034; A-11029), were used at 1:200 dilution. Images were acquired using an Olympus BX60 microscope, SPOT camera and SPOT^TM^ Advanced image software Version Mac: 4.6.1.26 (Diagnostic Instruments).

### Western blotting

Total cell extracts were prepared from approximately 5 × 10^6^ cells grown to a density of 1 × 10^6^ cells ml^−1^ in a six-well plate, resuspended in 100 μl of PBS and lysed with an equal volume of SDS sample buffer (2× concentrate, Sigma; S3401). Extracts, adjusted for equal protein loading, were resolved by SDS-PAGE (Laemmli, 1970) with subsequent immunoblotting and detection as described previously (Schmuckli-Maurer *et al*., 2010). The anti-TLR4 antibody (Santa Cruz-Biotechnology, sc-10741) was used at 1:1000 dilution and anti-tubulin antibody (Sigma, T9028) at 1:5000.

### RNA isolation and processing

Total RNA for analysis by microarray was isolated in triplicate from 30 ml of cultures of infected and uninfected cells and cells treated with LPS for 4 and 18 h. Cell pellets of 1.5–2.0 × 10^7^ cells were resuspended immediately in TRI Reagent (Sigma; T9424), and extracted according to the manufacturer's instructions. To eliminate possible contamination with genomic DNA, RNA samples used for RT-PCR and QRT-PCR were treated with DNase I (Qiagen; 79254) either before purification on a silica-gel membrane-based RNeasy® column (Qiagen; 74104) or by an on-column DNase I treatment. *A*_260_/*A*_280_ ratios obtained for all samples fell in the acceptable range of 1.78–2.1 and quality and integrity of the total RNA preparations was confirmed by gel electrophoresis.

### Custom bovine microarray and analysis

The oligonucleotide microarray used in this study included all bovine RNA RefSeq sequences available at NCBI (26 751) corresponding to the Btau 4.0 assembly of the bovine genome (http://www.ncbi.nlm.nih.gov/RefSeq/) and a number of putative bovine RNA Sequences and ESTs (4730) kindly provided by Kirsty Jensen, Roslin Institute, University of Edinburgh. Only extant sequences in the RefSeq mRNA database (December 2011) or UMD3.1 genome assembly were used in the analysis and this represents 19 777 sequences. Design of oligonucleotides, array synthesis, hybridization to the array and normalization of array data were carried out by Roche NimbleGen, Madison, USA. Each gene was represented by two identical sets of five 60-mer oligonucleotide probes which were isothermal with respect to melting temperature. cDNA was generated from 10 μg of total RNA using oligo(dT) primer and tagged with 3′-Cy3 dye after which 13 μg of labelled cDNA was hybridized to the array. Gene expression values were calculated from a RMA-normalized data set (Irizarry *et al*., 2003) and differentially expressed genes were identified using Rank Product Analysis (Breitling *et al*., 2004). Genes were defined as differentially expressed using the criteria of a false discovery rate of less than 5% and a fold change of greater than two. In addition to the bovine sequences, the array contained oligonucleotide probes representing the *T. annulata* genome, although these data were not analysed in the course of the present study. The data discussed in this publication have been deposited in NCBI's Gene Expression Omnibus (Edgar *et al*., 2002) and are accessible through GEO Series Accession No. GSE36428 (http://www.ncbi.nlm.nih.gov/geo/query/acc.cgi?acc=GSE36428).

To assess the relationship between the differentially expressed gene sets identified by RPA in TBL20 and LPS stimulated BL20 cultures, a Monte Carlo simulation was used to estimate the overlap expected to occur by chance (Metropolis and Ulam, 1949; Kabakchiev *et al*., 2010). A chi-squared test was used to statistically test whether the observed overlap was greater than that expected by chance.

In order to identify gene expression networks that were enriched for genes highlighted in this study, Ingenuity Pathway Analysis V9 (Ingenuity® Systems, http://www.ingenuity.com) was utilized when possible. Each bovine RNA sequence represented on the array was mapped to its corresponding gene in the Ingenuity Pathways Knowledge Base. The list of canonical pathways in the system was augmented with a number of specific pathways, namely a list of 453 validated and predicted NF-κB target genes (Sharif *et al*., 2007), the NF-κB pathway, the cMyc pathway, validated targets of cMyc transcriptional repression, validated targets of cMyc transcriptional activation, AP-1 target genes, ATF2 network genes and the WNT signalling pathway, largely derived from information in the NCBI BioSystems database. The significance of the association between novel gene lists and each pathway was measured in two ways. First, a ratio was calculated for the number of genes in a given list that map to the pathway divided by the total number of genes on the array that map to the same pathway. Second, Fischer's exact test was used to calculate a *P-*value to determine the probability that an association between a given gene list and a particular canonical pathway occurred by chance alone.

### Reverse transcription PCR (RT-PCR)

Relative expression levels of selected candidate genes of interest were analysed by semi-quantitative PCR using Superscript III-One Step RT-PCR (Invitrogen, 12574-026) and the standard manufacturer's protocol. Primers were designed and tested for specificity using NCBI's online program (http://www.ncbi.nlm.nih.gov/tools/primer-blast/index.cgi) and oligonucleotide properties calculator (http://www.basic.northwestern.edu/biotools/oligocalc.html). Primer sequences, GenBank accession numbers, annealing temperatures and product lengths for all gene-specific primers used in this study are given in Table S9. Primers were synthesized by Eurofins MWG Operon (Ebersberg, Germany) and whenever possible one of the primer pair spanned an intron/exon junction so that only cDNA was amplified or the primer pair amplified across at least one intron to give differentially sized genomic and cDNA products. Initial PCR optimization reactions were performed to ascertain the best annealing temperature (Tm), magnesium concentration and overall PCR efficiency. RT-PCR was performed in a final reaction volume of 25 μl with gene-specific primers at 200 nM and 12.5 ng of RNA template. Thermal cycling conditions were: an initial cycle of 50°C for 30 min, 94°C for 2 min, followed by 36 cycles of 94°C 15 s, X°C for 30 s (where X°C indicates the experimentally determined annealing temperature) and 72°C for 1 min; a final extension step was performed at 72°C for 10 min.

RT-PCR products were resolved relative to a 100 bp DNA ladder by electrophoresis through ethidium bromide-stained 1% TAE agarose gels. For all semi-quantitative RT-PCR assays, the expression levels of candidate genes were normalized to the levels obtained with primers for the genes encoding Beta actin (ACTβ) or glyceraldehyde-3-phosphate dehydrogenase (GAPDH). These constitutively expressed genes did not show significant changes on the present array experiment and have been used previously as normalizing genes for bovine gene expression studies in *T. annulata*-infected cells (Sager *et al*., 1997).

SYBR QRT-PCR methodology was utilized to quantify more accurately the relative fold change in mRNA expression for selected differentially expressed genes. cDNA synthesis was carried out using 2 μg of DNase I-treated total RNA and an Affinityscript qPCR cDNA synthesis kit (Stratagene, USA; 600559) with oligo(dT) primers, according to the manufacturer's instructions. Control reactions without reverse transcriptase were always included. Real-time florescence detection was performed using 1 μl of cDNA with Brilliant SYBR Green qPCR Master Mix buffer (Stratagene, 600548) according to the manufacturer's protocol. The final concentration of each primer was 200 nM. A Stratagene Mx3005P Real-Time PCR System was used with the following thermal cycling parameters: 10 min at 95°C; 40 cycles of 30 s at 95°C (denaturation), 30 s annealing at X°C (Tm of primer pairs minus 3°C) and 30 s at 72°C (extension). After 40 cycles of amplification, a melting curve analysis was carried out to verify the correct product by its specific melting temperature (Tm). All qPCR data were captured and analysed by Stratagene MxPro v4.10 software. Quantitative values were normalized relative to those obtained for housekeeping genes, either β-actin or GAPDH, and fold changes calculated relative to the calibrator (DMSO-treated uninfected BL20 cells) using −2^−ΔΔCt^ equation (Livak and Schmittgen, 2001). Statistical analysis and generation of graphs were performed using a two-tailed Student's *t*-test with GraphPad Prism Software version 5.0 (La Jolla, CA).

## Supporting Information

**Fig. S1.** Immunofluorescence analysis of activated BL20 cells in response to LPS stimulation. Panels 1–3: IKK signalosomes were detected in BL20 and LPS stimulated BL20 using a monoclonal anti-IKKγ antibody. IKK signalosomes (green dots) were observed only in BL20 LPS. DAPI nuclear staining is shown in panel 3. The arrow denotes a cluster of IKK signalosomes. Panels 4–6: NF-κB was detected in BL20 and LPS stimulated BL20 using anti-p65 antibody. Elevated expression and nuclear localization of NF-κB was observed in BL20 LPS. DAPI nuclear staining is shown in panel 6. Bar = 15 μm. ****Fig. S2**. Panel of qRT-PCR results.** **Fig. S3**. Top ranking cellular and molecular functions from pathway analysis. **Table S1.** Full list of genes in profile 1 (*n* = 32). **Table S2.** Full list of genes in profile 2 (*n* = 180). **Table S3.** Full list of genes in profile 3 (*n* = 154). **Table S4.** Full list of genes in profile 4 (*n* = 55). **Table S5.** Full list of genes in profile 5 (*n* = 38). **Table S6.** Full list of genes in profile 6 (*n* = 13). **Table S7.** Full list of genes in profile 7 (*n* = 650). **Table S8.** Full list of genes in profile 8 (*n* = 98).
