# Supplementary material for: Modulation of activation-associated host cell gene expression by the apicomplexan parasite Theileria annulata
Source: Cell Microbiol. 2012 May 23;14(9):1434–54. doi: 10.1111/j.1462-5822.2012.01809.x (PMC3532605; doi:10.1111/j.1462-5822.2012.01809.x)
Supplement: Supplementary file 1 [file cmi0014-1434-SD1.pdf]

Supplementary data figure 1. Immunofluorescence analysis of activated BL20 cells in response to LPS stimulation

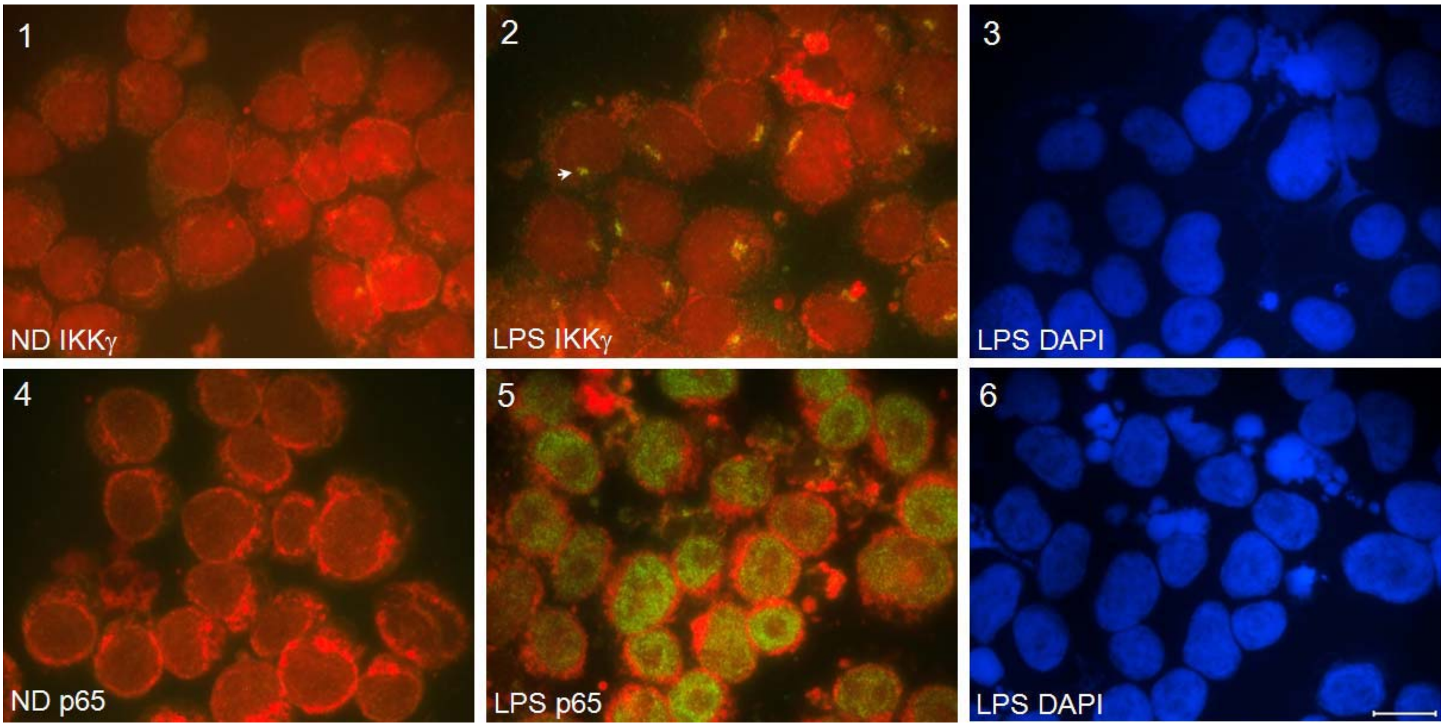

Panels 1-3: IKK signalosomes were detected in BL20 and LPS stimulated BL20 using a monoclonal anti-IKKγ antibody. IKK signalosomes (green dots) were observed only in BL20 LPS. DAPI nuclear-staining is shown in panel 3. The arrow denotes a cluster of IKK signalosomes.

Panels 4-6: NFκB was detected in BL20 and LPS stimulated BL20 using anti-p65 antibody. Elevated expression and nuclear localisation of NFκB was observed in BL20 LPS. DAPI nuclear-staining is shown in panel 6. Bar = 15 µm.

Supplementary data figure 2. Panel of qRT-PCR results

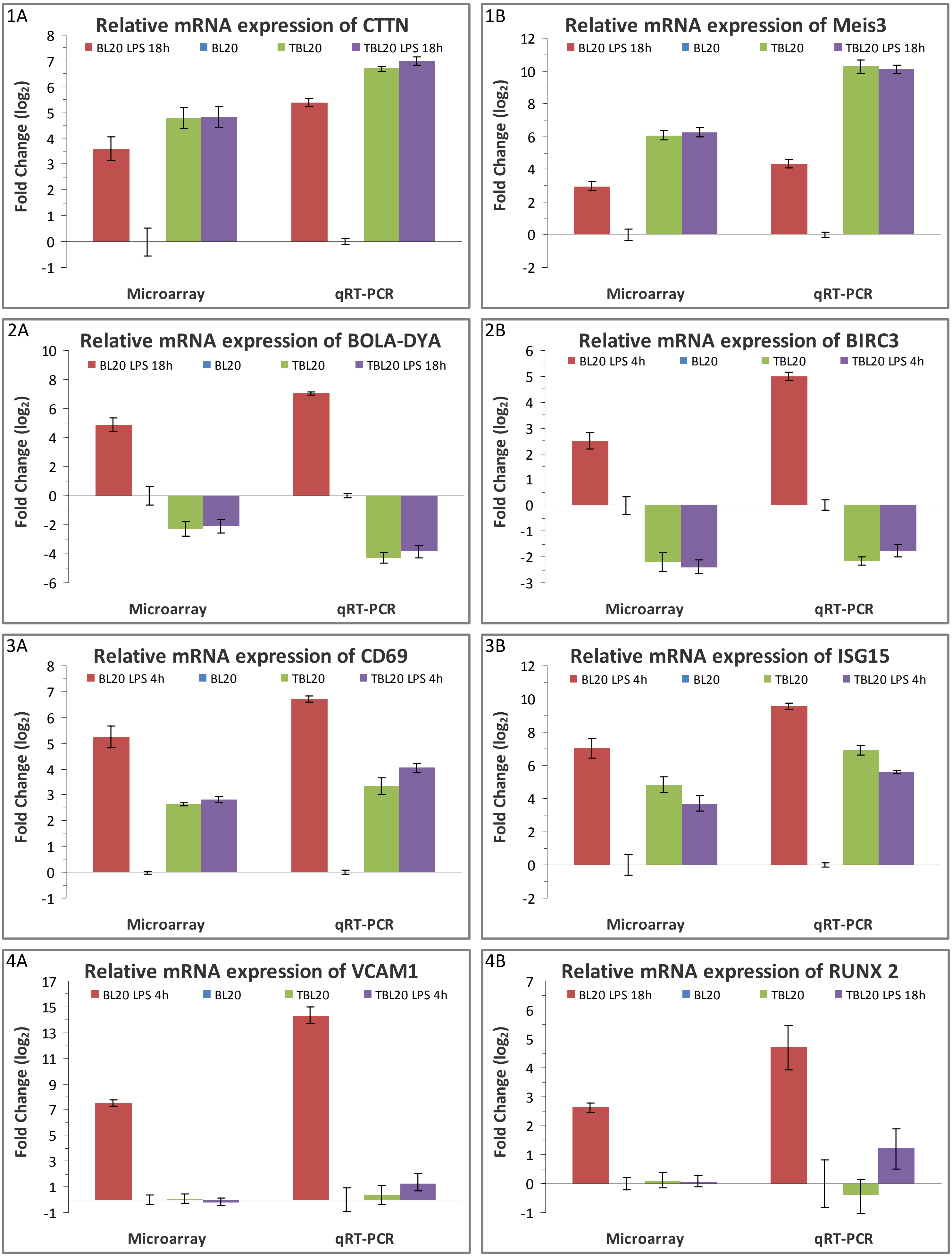

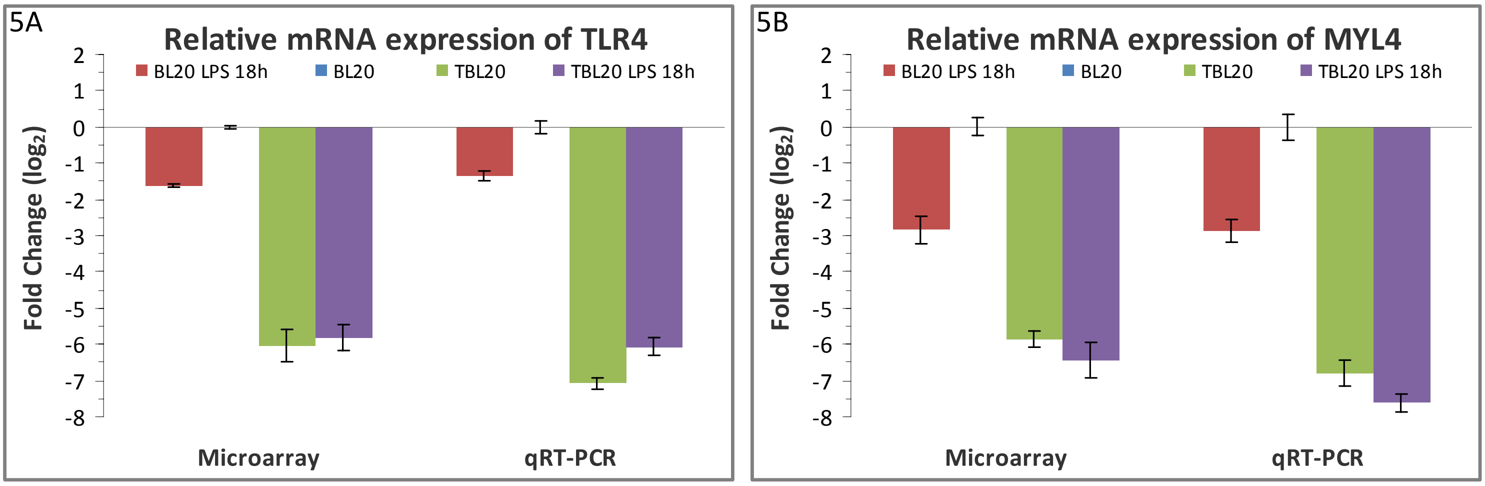
5A
Relative mRNA expression of TLR4
BL20 LPS 18h
BL20
TBL20
TBL20 LPS 18h
Fold Change (log_2)
Microarray
qRT-PCR
5B
Relative mRNA expression of MYL4
BL20 LPS 18h
BL20
TBL20
TBL20 LPS 18h
Fold Change (log_2)
Microarray
qRT-PCR

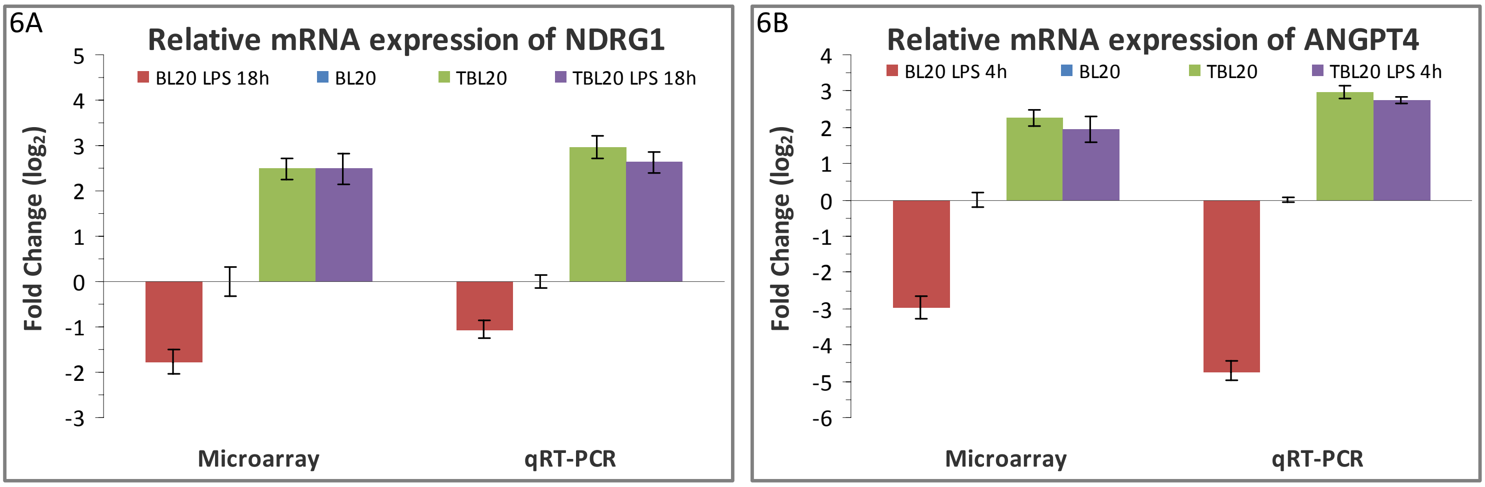
6A
Relative mRNA expression of NDRG1
BL20 LPS 18h
BL20
TBL20
TBL20 LPS 18h
Fold Change (log_2)
Microarray
qRT-PCR
6B
Relative mRNA expression of ANGPT4
BL20 LPS 4h
BL20
TBL20
TBL20 LPS 4h
Fold Change (log_2)
Microarray
qRT-PCR

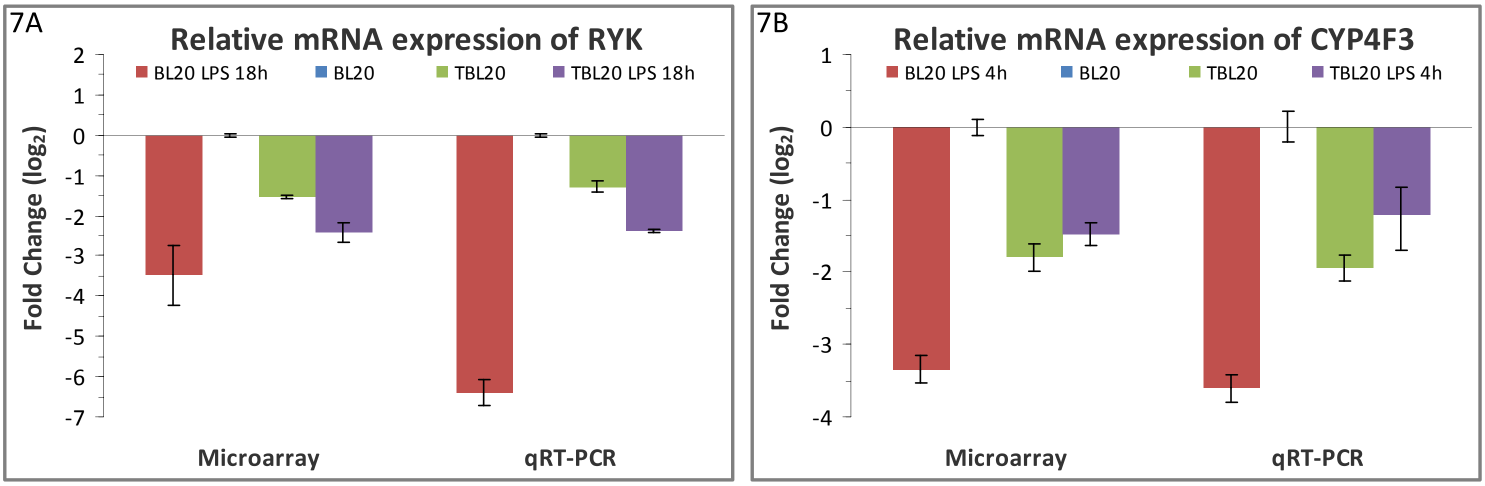
7A
Relative mRNA expression of RYK
BL20 LPS 18h
BL20
TBL20
TBL20 LPS 18h
Fold Change (log_2)
Microarray
qRT-PCR
7B
Relative mRNA expression of CYP4F3
BL20 LPS 4h
BL20
TBL20
TBL20 LPS 4h
Fold Change (log_2)
Microarray
qRT-PCR

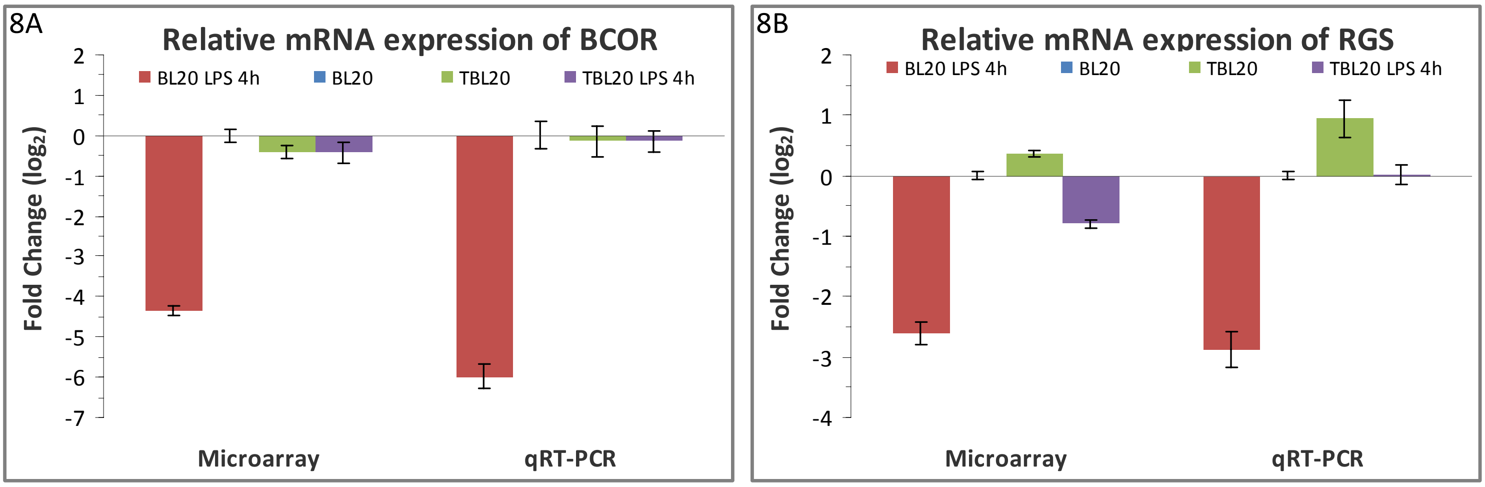
8A
Relative mRNA expression of BCOR
BL20 LPS 4h
BL20
TBL20
TBL20 LPS 4h
Fold Change (log_2)
Microarray
qRT-PCR
8B
Relative mRNA expression of RGS
BL20 LPS 4h
BL20
TBL20
TBL20 LPS 4h
Fold Change (log_2)
Microarray
qRT-PCR

Supplementary data figure 3. Top ranking cellular and molecular functions from pathway analysis

Category	p-value	Molecules
**Profile 1**		
Cellular Movement	$7.23x10^{-6}$	ELN, SELL, ICAM1, SLC2A1, TP73, XCL2, EGLN3, MMP13, SDC4, IL2RB, CTTN, CCL1
Cell-To-Cell Signaling and Interaction	$1.47x10^{-5}$	SELL, STXBP1, ICAM1, TP73, MMP13, SDC4, CTTN, IL2RB
Antigen Presentation	$5.3x10^{-5}$	ELN, SELL, ICAM1, EGLN3, CCL1
Cellular Compromise	$6.82x10^{-5}$	SELL, ICAM1, TP73, ATXN1, IL2RB, CTTN
Cell Signaling	$5.38x10^{-4}$	ELN, SELL, ICAM1, TP73, XCL2, CCL1
Molecular Transport	$5.38x10^{-4}$	ELN, SELL, STXBP1, ICAM1, SLC2A1, TP73, SLC6A6, XCL2, ABCG1, CCL1
Vitamin and Mineral Metabolism	$5.38x10^{-4}$	ELN, SELL, ICAM1, TP73, XCL2, CCL1
Cellular Growth and Proliferation	$1.49x10^{-3}$	ELN, ICAM1, SLC2A1, SLAMF7, TP73, EGLN3, ABCG1, IL2RB
Cell Morphology	$1.6x10^{-3}$	SELL, ICAM1, TP73, SDC4, CTTN, CCL1
Cellular Development	$1.6x10^{-3}$	SELL, ICAM1, TP73, EGLN3, MMP13, SDC4, IL2RB, CTTN, CCL1
**Profile 2**		
Cellular Growth and Proliferation	$2.21x10^{-4}$	GAB2, TBXA2R, null, MERTK, NGFR, IGF1R, COL18A1, ADRA1B, IGFBP6, CALCRL, PTPRG, LAIR1, ABCG2, LCN2, CD36, TRPC6, GGT1, PIGR, IL24, TLR4, MARCKSL1, IL18 (includes EG:16173), Gulo, DBC1, RASGRP1, NFE2, SH2B2, S1PR1, LEF1, PDGFD
Cellular Compromise	$3.76x10^{-4}$	TLR4, HSPB2, NGFR, NFE2, ATP2A1, IGF1R, COL18A1, null, GPX7, SGCA, IL24
Cellular Function and Maintenance	$3.76x10^{-4}$	GAB2, ABCG2, ATP2A1, CD36, TRPC6, IL24, PIGR, FCGR1A, ZNF423, TLR4, IL18 (includes EG:16173), MERTK, NGFR, SH2B2, IGF1R, S1PR1, RGNEF, COL18A1, CCR6, null
Cell Death	$4.94x10^{-4}$	GAB2, POU2AF1, HSPB2, ATP2A1, TBXA2R, TMEM132A, SGCA, FCGR1A, C16orf5, RPS3A, MERTK, RGS5, NGFR, IGF1R, ALPK2, COL18A1, IGFBP6, PTPRG, CALCRL, LAIR1, ABCG2, LCN2, CD36, SGMS2, F13A1, GGT1, IL24, GPX7, PIGR, TLR4, ZNF423, IL18 (includes EG:16173), EBF1, NFE2, RASGRP1, DBC1, PPP2R2B, S1PR1, LEF1, ABCC3
Cellular Development	$5.41x10^{-4}$	GAB2, TCN2, LMO2, POU2AF1, TBXA2R, CDH23, IGF2BP1, MBNL3, RPS3A, POU6F2, RGS5, MERTK, NGFR, IGF1R, COL18A1, CCR6, IGFBP6, PTPRG, CTSK, LAIR1, ABCG2, LCN2, CD36, TRPC6, GGT1, IL24, ZNF423, TLR4, IL18 (includes EG:16173), EBF1, RASGRP1, NFE2, SH2B2, S1PR1, LEF1, CPEB1
Cellular Response to Therapeutics	$6.24x10^{-4}$	ABCG2, ABCC3
Cell-To-Cell Signaling and Interaction	$7.3x10^{-4}$	GAB2, POU2AF1, TBXA2R, CDH23, MMRN1, null, FCGR1A, MERTK, IGF1R, AMICA1, COL18A1, CCR6, NINJ2, CALCRL, LAIR1, ABCG2, LCN2, CD36, F13A1, GGT1, IL24, PIGR, MARCKSL1, TLR4, IL18 (includes EG:16173), NFE2, RASGRP1, S1PR1, MYO1F, null, null
Cellular Movement	$9.31x10^{-4}$	GAB2, TBXA2R, null, IGF2BP1, NGFR, IGF1R, COL18A1, CCR6, IGFBP6, CALCRL, CTSK, LCN2, CD36, TRPC6, IL24, PIGR, TLR4, ZNF423, EBF1, IL18 (includes EG:16173), RASGRP1, S1PR1, MYO1F, LEF1, PDGFD, null
Amino Acid Metabolism	$1.3x10^{-3}$	ABCG2, FOLR2, ABCC3, GGT1
Drug Metabolism	$1.3x10^{-3}$	IL18 (includes EG:16173), ABCG2, FOLR2, GGT1, ABCC3
**Profile 3**		
Cell-To-Cell Signaling and Interaction	$3.51x10^{-5}$	BMP4, WNT10B, PDGFA, CUBN, SOCS2, CLU, PCDHGB4, HES1 (includes EG:15205), CD163, TNXB, LYST, TNFSF9, MARCKS, ICOSLG, FGR, MUC1, RGMB, GPR44, EPAS1, PLXNC1, THBS1, CHN1, CD200R1, IL15 (includes EG:16168), CD48, BAMBI, IFNAR2, ITGB3, KNG1, CD80 (includes EG:12519), VAV3, IRF8
Cell Morphology	$5.42x10^{-5}$	GAS7, WNT10B, BMP4, PDGFA, THBS1, CHN1, SOCS2, HES1 (includes EG:15205), ITGB3, UBD, KNG1, LYST, ICAM5, VAV3, CEBPA, ARHGAP31, PLEK, MARCKS, GBP2 (includes EG:14469), FGR
Cellular Development	$6.32x10^{-5}$	BMP4, WNT10B, PDGFA, CLU, SSBP2, HES1 (includes EG:15205), SIGLEC10, CD163, CDKN2B, TNFSF9, CEBPA, GBP2 (includes EG:14469), MARCKS, PLAC8, FGR, ICOSLG, MUC1, EPAS1, CHN1, THBS1, IL15 (includes EG:16168), STAP1, IKBKE, IFNAR2, ITGB3, KNG1, CD80 (includes EG:12519), VAV3, IRF8, MYO1E
Cellular Movement	$7.05x10^{-5}$	HTATIP2, BMP4, RGS18, PDGFA, SOCS2, CLU, LYST, TNFSF9, ADAM19, MARCKS, ICOSLG, FGR, MUC1, GPR44, CD207, EPAS1, THBS1, IL15 (includes EG:16168), CD48, ITGB3, PRKCG, KNG1, CD80 (includes EG:12519), VAV3, RPS6KA5, IRF8
Cellular Growth and Proliferation	$7.26x10^{-5}$	WNT10B, BMP4, PDGFA, SOCS2, CLU, SSBP2, SIGLEC10, HES1 (includes EG:15205), CD163, CDKN2B, RASAL1, TNFSF9, CEBPA, GBP2 (includes EG:14469), PLAC8, ICOSLG, MUC1, TP53INP1, EPAS1, THBS1, SAMSN1, IL15 (includes EG:16168), CD48, BAMBI, IKBKE, IFNAR2, ITGB3, PRKCG, KNG1, CD80 (includes EG:12519), RPS6KA5, IRF8
Cellular Function and Maintenance	$1.28x10^{-4}$	MUC1, BMP4, PDGFA, CHN1, THBS1, IL15 (includes EG:16168), CLU, IKBKE, CD163, IFNAR2, ITGB3, KNG1, CD80 (includes EG:12519), ICAM5, VAV3, CEBPA, MARCKS, PLEK, IRF8, FGR, ICOSLG

Cellular Compromise	$1.48x10^{-4}$	UBD, BMP4, CD80 (includes EG:12519), THBS1, IL15 (includes EG:16168), CLU, IRF8, FGR, ITGB3
Cell Death	$1.9x10^{-4}$	HTATIP2, BMP4, PDGFA, SGK1, SOCS2, CLU, HES1 (includes EG:15205), CDKN2B, TNFSF9, EAF2, CEBPA, BIRC3, PLAC8, ICOSLG, FGR, EPHA7, MUC1, GPR44, TP53INP1, EPAS1, THBS1, IL15 (includes EG:16168), CD48, IKBKE, IFNAR2, PRKCG, ITGB3, UBD, KNG1, NEK8, FABP6, CD80 (includes EG:12519), VAV3, RPS6KA5, IRF8, NTSR1
Cellular Assembly and Organization	$3.43x10^{-4}$	GAS7, CD207, SEPT5, BMP4, AP1M2, THBS1, CHN1, PDGFA, SOCS2, CD48, CLU, TNXB, ITGB3, KNG1, LYST, VAV3, ICAM5, MARCKS, PLEK, FGR
Gene Expression	$6.22x10^{-4}$	MUC1, RGMB, HTATIP2, EPAS1, BMP4, PDGFA, SGK1, SOCS2, IKBKE, HES1 (includes EG:15205), IFNAR2, RFX5, MEF2B, ITGB3, KNG1, CD80 (includes EG:12519), VAV3, EAF2, CEBPA, RPS6KA5, IRF8, SIM1 (includes EG:20464), FGR, PKIG
**Profile 4**		
Cellular Movement	$9.98x10^{-5}$	MTSS1, NDRG1, PIK3R5, LAMC1, PRF1, PAK1, HCK, ABI3, SPARC, ENPP2, TNFRSF1B, SEMA3F, CCR7
Cellular Function and Maintenance	$2.33x10^{-4}$	GZMA, PAK1, PRF1, HCK, TNFRSF1B, FOXA3, RNASEL, CCR7
Cellular Development	$6.98x10^{-4}$	LAMC1, GZMA, PRF1, PAK1, NDRG1, HCK, SPARC, MLLT3, TNFRSF1B, RNASEL, CCR7
Cellular Growth and Proliferation	$6.98x10^{-4}$	SUMO3, ENTPD1, NDRG1, LAMC1, PRF1, PAK1, HCK, SPARC, ENPP2, MLLT3, TNFRSF1B, SEMA3F, CCR7
Cell Death	$8.61x10^{-4}$	GZMA, PDE4C, NDRG1, RNASEL, PAK1, PRF1, ANTXR2, HCK, SPARC, MLLT3, TNFRSF1B, SEMA3F, CCR7, STEAP3, EPHX1
Cellular Assembly and Organization	$1.5x10^{-3}$	LAMC1, PSTPIP1, PRF1, PAK1, MTSS1, HCK, DGKG, SPARC, ENPP2, SEMA3F, CCR7
Cell-To-Cell Signaling and Interaction	$2.41x10^{-3}$	GZMA, LAMC1, PRF1, PAK1, NDRG1, ENTPD1, HCK, SPARC, AP3M2, TNFRSF1B, SEMA3F, CCR7
Carbohydrate Metabolism	$2.93x10^{-3}$	CMAH, ENPP2
Cell Morphology	$2.93x10^{-3}$	GZMA, PRF1, PAK1, ENTPD1, MTSS1, NDRG1, HCK, SPARC, AP3M2
Cellular Compromise	$2.93x10^{-3}$	GZMA, PAK1, PRF1, ENTPD1, NDRG1, MTSS1, SLA, HCK, TNFRSF1B, SEMA3F
**Profile 5**		
Cellular Movement	$9.36x10^{-7}$	SMAD3, CD69, TGFBR3, RELB, ITGA6, TNFAIP3, NFKB2, CX3CR1, NFKBIZ, IL7, CXCL10, JUN, MYO10
Cell-To-Cell Signaling and Interaction	$1.67x10^{-6}$	TIFA, TGFBR3, SMAD3, CD69, RELB, ACTA2, ITGA6, TNFAIP3, CD83, NFKB2, CX3CR1, BST2, IL7, CXCL10, JUN, BAIAP2, DDX58
Post-Translational Modification	$4.13x10^{-6}$	USP18, ITGA6, ISG15
Protein Folding	$4.13x10^{-6}$	USP18, ISG15
Gene Expression	$8.61x10^{-6}$	MX2, RELB, SMAD3, TGFBR3, ZBP1, ITGA6, TNFAIP3, NFKB2, NFKBIZ, IRF5, BST2, MAFF, IL7, JUN, PLK2, DDX58
Cell Death	$2.95x10^{-5}$	OAS1, USP18, INPP1, CD69, SMAD3, TGFBR3, RELB, ITGA6, TNFAIP3, CX3CR1, NFKB2, NFKBIZ, IRF5, IL7, ISG15, CXCL10, JUN, PLK2, DDX58
Cellular Development	$3.22x10^{-5}$	TGFBR3, SMAD3, CD69, RELB, ITGA6, EXT1, CD83, NFKB2, IL7, MAFF, CXCL10, JUN, PLK2, BAIAP2
Cell Signaling	$6.87x10^{-5}$	TIFA, TGFBR3, PLK2, TNFAIP3, BST2
Antigen Presentation	$1.5x10^{-4}$	CXCL10, CD69, RELB, SMAD3, DDX58, TNFAIP3, CD83, NFKB2, CX3CR1, IL7
Cellular Growth and Proliferation	$1.55x10^{-4}$	SMAD3, TGFBR3, RELB, ITGA6, CD83, NFKB2, IL7, BST2, MAFF, ISG15, CXCL10, JUN, PLK2
**Profile 6**		
Lipid Metabolism	$2.69x10^{-4}$	CYP3A4, FLT1, CYP4F3
Small Molecule Biochemistry	$2.69x10^{-4}$	CYP3A4, FLT1, CYP4F3, DNTT, STXBP6
Cell Cycle	$8x10^{-4}$	FLT1
Cell Morphology	$8x10^{-4}$	AQP3, TSPAN7, FLT1, RYK
Cell-To-Cell Signaling and Interaction	$8x10^{-4}$	PGLYRP1, FLT1
Cellular Movement	$8x10^{-4}$	FLT1
Drug Metabolism	$8x10^{-4}$	CYP3A4, STXBP6
Molecular Transport	$8x10^{-4}$	AQP3, CYP3A4, FLT1, STXBP6
Vitamin and Mineral Metabolism	$8x10^{-4}$	CYP3A4
Carbohydrate Metabolism	$1.6x10^{-3}$	CYP3A4, FLT1

**Profile 7**		
Cellular Development	9.99×10^{-6}	OSTN, SOCS1, CTGF, SP7, CDK5R1, IFIH1, MYLK, NCAM2, BAIAP2, CCR8, RIPK2, TEK, KLRC1, ITK, SPP1 (includes EG:20750), ATF3, CHN1, IL6R, TYK2, TDRD7, RORC, ANXA2, TSPAN33, JUNB, ADAR, PPP1R13L, CCL4, CD40, GCNT1, FSCN1, PTPRB, CFLAR, POU1F1, SNCA, B2M, CRLF2, LEPR, KLRD1, BATF, ROBO1, NOTCH2, NFKBIA, LZTS1, CREB1, KLRA1, MYO1A, ROBO2, TRAF1, HEY1, NCKAP1, VCAM1, IRF4, TP63, C5, NFATC1, FABP2, ZBTB32, PRKCI, NR4A2, IL12B, CSF1 (includes EG:12977), CDKN1A, NR4A1, REC8 (includes EG:290227), COL11A1
Cell Death	1.34×10^{-5}	SOCS1, CTGF, DENND4A, KLF6, null, CDK5R1, MYLK, IFIH1, CASR, UBA7, TRAF4, NEK6, CCR8, RIPK2, MVP, KLRC1, ITK, NOVA1, SPP1 (includes EG:20750), ATF3, DDX4, Prune2 (mouse), SNAI1, TYK2, IL6R, null, RORC, IRF9, JUNB, ADAR, NCOA3, UBD, CCL4, PPP1R13L, CD40, GCNT1, NUP210, MAPK10, STAT2, CFLAR, ITGA7, SNCA, B2M, TNFAIP8, CA2, SNX33, INPP1, KLRD1, LEPR, CASP4, TANK, NOTCH2, NFKBIA, HLA-A, TNIP2, STK17A, CREB1, KLRA1, IFI6, EYA4, CFH, GABRA2, FAP, B4GALT5, TRAF1, HEY1, NCKAP1, VCAM1, OAS1, IRF4, TP63, DVL1, C5, NFATC1, UACA, SERPINB4, CDC42BPA, GABRG2, PRKCI, NR4A2, CSF1 (includes EG:12977), CAPRIN2, IL12B, CDKN1A, NR4A1, MAP3K8, EIF2AK2
Gene Expression	2.34×10^{-5}	SOCS1, RGS18, VPS39, LEPR, NFKBIE, BATF, KLF6, ELL3, TANK, CASR, NOTCH2, NFKBIA, BCAT2, NEK6, CREB1, RIPK2, VCAM1, IRF4, TP63, ATF3, SPP1 (includes EG:20750), IL6R, DVL1, IRF9, NFATC1, JUNB, NCOA3, GPR183, CXCL16, ZBTB32, NR4A2, CD40, CSF1 (includes EG:12977), CDKN1A, NR4A1, MAP3K8, STAT2, NRIP1, EIF2AK2, CFLAR, POU1F1
Cellular Movement	2.64×10^{-5}	SOCS1, CTGF, EPS8, TCIRG1, SP7, null, CDK5R1, MYLK, CASR, LCAT, CCR8, TEK, ITK, SPP1 (includes EG:20750), SNAI1, IL6R, ANXA2, NCOA3, CCL4, CD40, GCNT1, FSCN1, TNFAIP8, AOC3, CLDN11, RGS18, ROBO1, PARP9, NFKBIA, KLRA1, ERRFI1, ROBO2, CFH, FAP, HEY1, NCKAP1, VCAM1, TP63, CD58, C5, NFATC1, CXCL16, PRKCI, NR4A2, CSF1 (includes EG:12977), IL12B, CDKN1A, MAP3K8, null
Cellular Function and Maintenance	2.93×10^{-5}	B2M, SOCS1, EPS8, CRLF2, KLRD1, BATF, MYLK, NFKBIA, PLS3, CREB1, CCR8, RIPK2, ITK, KLRC1, TRAF1, SPP1 (includes EG:20750), IRF4, KALRN, TYK2, IL6R, RORC, ANXA2, NFATC1, JUNB, CDC42BPA, CCL4, PRKCI, CD40, IL12B, CSF1 (includes EG:12977), FSCN1, CDKN1A, NR4A1, COL11A1, CFLAR, COL3A1
Carbohydrate Metabolism	5.61×10^{-5}	SOCS1, OSTN, DYRK2, CASR, CTGF, CD40, LCAT, LEPR, CSF1 (includes EG:12977), PPP1R3C, NR4A1, TEK
Cell-To-Cell Signaling and Interaction	9.34×10^{-5}	SOCS1, CTGF, TCIRG1, JAM2, MLLT4, null, CDK5R1, IFIH1, CASR, NCAM2, LPL, CCR8, TIMD4, RIPK2, TEK, KLRC1, ITK, BLOC1S3, SPP1 (includes EG:20750), TYK2, IL6R, ANXA2, JUNB, CCL4, CD40, GCNT1, PTPRB, STAT2, SNCA, ITGA7, C3AR1, AOC3, B2M, CLDN11, NLGN3, KLRD1, ROBO1, AICDA, IDH1, NFKBIA, HLA-A, KLRA1, CFH, ROBO2, VCAM1, TP63, CD58, NFATC1, C5, CXCL16, CSF1 (includes EG:12977), IL12B, CDKN1A, NR4A1, MAP3K8, EIF2AK2, IRAK2
Cellular Growth and Proliferation	9.59×10^{-5}	SOCS1, EPS8, CTGF, INSIG1, TCIRG1, KLF6, CDK5R1, IFIH1, CASR, LCAT, TRAF4, TIMD4, RIPK2, TEK, MVP, KLRC1, ITK, ATF3, SPP1 (includes EG:20750), ANGPTL6, DDX4, IL6R, TYK2, RORC, ANXA2, JUNB, NCOA3, CCL4, CD40, TES, GCNT1, FSCN1, CFLAR, POU1F1, C3AR1, SNCA, ITGA7, B2M, TNFAIP8, CLDN11, CRLF2, KLRD1, LEPR, BATF, AICDA, NOTCH2, NFKBIA, LZTS1, CREB1, TRAF1, HEY1, VCAM1, CACNA1D, IRF4, TP63, CD58, NFATC1, C5, CXCL16, ZBTB32, FABP2, IL12B, CSF1 (includes EG:12977), TBC1D8, CDKN1A, NR4A1, EIF2AK2, NRIP1, null
Protein Synthesis	3.11×10^{-4}	B2M, SPP1 (includes EG:20750), TP63, ATF3, DDX4, C5, CD40, CREB1, LPL, CDKN1A, MYO1A, CFH, POU1F1, TRAF1, NCKAP1
Cell Cycle	3.57×10^{-4}	SOCS1, SNX33, NOTCH2, NFKBIA, LZTS1, CREB1, NEK6, DSTN, ERRFI1, VCPIP1, TP63, ATF3, SPP1 (includes EG:20750), IL6R, null, IRF9, C5, NFATC1, JUNB, NCOA3, UBD, NR4A2, CD40, IL12B, CSF1 (includes EG:12977), PTPRB, CDKN1A, NR4A1, SIK1, REC8 (includes EG:290227), MAP3K8, NRIP1, EIF2AK2, null, null
**Profile 8**		
Cell Signaling	8.36×10^{-5}	RGS1, SPP1 (includes EG:20750), F2R, TRIM9, HBEGF, GRK5, MAPK13, FCGR2B, PDE4D, SV2A, LTB4R, ARRB2, CARD10, CD5, SPINT2, TLR3, CARD11, FCGR3A, ADRB2
DNA Replication, Recombination, and Repair	8.36×10^{-5}	IGFBP4, ARRB2, SPP1 (includes EG:20750), F2R, MAF, HBEGF, TLR3, PDE4D, ADRB2
Nucleic Acid Metabolism	8.36×10^{-5}	ARRB2, F2R, SLC19A1, GRK5, PDE4D, CTPS, ADRB2
Small Molecule Biochemistry	8.36×10^{-5}	IGFBP4, SPP1 (includes EG:20750), SLC7A11, SLC1A4, F2R, MFNG, SLC1A5, HBEGF, GRK5, FCGR2B, PDE4D, CTPS, SYNJ2, ARRB2, LTB4R, CD5, PHGDH, SLC19A1, PDK2, PDK1, ADRB2
Amino Acid Metabolism	4.36×10^{-4}	SLC1A4, SLC7A11, SLC1A5, PHGDH, SLC19A1
Molecular Transport	4.36×10^{-4}	RGS1, SPP1 (includes EG:20750), SLC7A11, SLC1A4, F2R, TRIM9, SLC1A5, GRK5, FCGR2B, PDE4D, SV2A, ARRB2, LTB4R, CD5, PHGDH, SPINT2, SLC19A1, TLR3, FCGR3A, ADRB2
Cellular Movement	4.36×10^{-4}	RGS1, IGFBP4, PODXL, SPP1 (includes EG:20750), F2R, HBEGF, FCGR2B, PDE4D, SERPINE2, LTB4R, ARRB2, STARD13, PCSK4, SPINT2, IGSF8, TLR3, PLCL1, TNS1, FCGR3A, E2F2, ETV5, ADRB2
Vitamin and Mineral Metabolism	4.38×10^{-4}	RGS1, SPP1 (includes EG:20750), F2R, HBEGF, GRK5, FCGR2B, LTB4R, ARRB2, CD5, SLC19A1, SPINT2, TLR3, FCGR3A, ADRB2
Cellular Assembly and Organization	1.82×10^{-3}	PODXL, SPP1 (includes EG:20750), F2R, HBEGF, GRK5, SERPINE2, SYNJ2, LTB4R, SORBS1, STARD13, FGD4, MAP1LC3A, ARHGEF3, TNS1, ARHGEF9, ADRB2
Cellular Growth and Proliferation	1.87×10^{-3}	IGFBP4, SPP1 (includes EG:20750), SLC7A11, F2R, SPRY1, MAF, HBEGF, GRK5, ARNT2, FCGR2B, PDE4D, SERPINE2, LTB4R, ANKRD1, CD5, STARD13, PCSK4, SPINT2, SLC19A1, TLR3, CARD11, E2F2, FCGR3A, ADRB2

Supplementary data table 1 Full list of genes in profile 1(n = 32)

Profile_ S. No	SEQ. ID	Acc. No. (RefSeq)	Putative Gene symbol	Gene Name	BL20 LPS Vs BL20 FDR (4h/or18)	BL20 LPS Vs BL20 Abs: FC (4h/or18)	TBL20 Vs BL20 Abs: FC	TBL20 Vs BL20 LPS Abs: FC (4h/or18)	BW720c Response
						↑	↑↑		
1_1	gi_119889107	XM_001256711	SLAMF7	Slam family member 7	0.00	2.8	415.4	148.0	---
1_2	gi_119903692	XM_871827	LRRTM4	Leucine rich repeat transmembrane neuronal 4	0.00	3.6	242.6	67.9	---
1_3	gi_31342133	NM_174389	MMP13	Matrix metallopeptidase 13 (collagenase 3)	0.00	3.1	168.2	54.2	↓
1_4	gi_31342239	NM_175716	XCL2	Chemokine (c motif) ligand 1	0.00	8.5	356.6	42.0	↓
1_5	gi_119927959	XM_593064	TP73	Tumor protein p73	0.00	2.7	53.6	20.0	---
1_6	gi_139948768	NM_001083791	SH3BGRL2	SH3 domain binding glutamic acid-rich protein like 2	0.00	3.2	45.0	14.0	---
1_7	gi_119894051	XM_587301	IL2RB	Interleukin 2 receptor, beta	0.00	8.3	93.1	11.2	---
1_8	gi_27901800	NM_174182	SELL	Selectin L	0.01	2.2	21.4	9.6	↑
1_9	gi_119911540	XM_001253061	CCL1	Chemokine (c-c motif) ligand 1	0.00	3.0	27.1	9.2	↓
1_10	gi_119910732	XM_869544	MEIS3	Meis homeobox 3	0.00	8.3	71.0	8.5	↓
1_11	gi_31342240	NM_174348	ICAM1	Intercellular adhesion molecule 1	0.00	3.8	22.8	6.0	---
1_12	gi_158341675	NM_001109983	PGPEP1	Pyroglutamyl-peptidase i	0.00	4.8	22.4	4.7	---
1_13	gi_119908880	XM_001253453	LOC785427	Similar to epidermis-specific serine protease-like protein	0.00	4.7	22.1	4.7	↑
1_14	gi_119900312	XM_612930	BNC2	Basonuclin 2	0.00	4.9	22.6	4.6	---
1_15	gi_114051048	NM_001046491	MOBKL2B	Mob1, mps one binder kinase activator-like 2b (yeast)	0.01	2.1	8.6	4.1	---
1_16	gi_31341516	NM_174619	STXBP1	Syntaxin binding protein 1	0.00	2.9	11.2	3.9	---
1_17	gi_119891399	XM_599768	JHDM1D	Jumonji c domain containing histone demethylase 1 homolog d (s. Cerevisiae)	0.00	2.7	9.9	3.7	---
1_18	gi_119915826	XM_868412	ATXN1	Ataxin 1	0.00	3.5	12.8	3.6	---
1_19	gi_119903194	XM_001255518	IL18RAP	Interleukin 18 receptor accessory protein	0.00	4.1	14.3	3.5	↓
1_20	gi_119914616	XM_597868	LOC519644	Similar to voltage-gated calcium channel alpha(2)delta-3 subunit	0.01	2.1	7.3	3.4	↑
1_21	gi_115495042	NM_001075961	RTP4	Receptor (chemosensory) transporter protein 4	0.00	4.5	14.9	3.3	↑
1_22	gi_31341539	NM_174610	SLC6A6	Solute carrier family 6 (neurotransmitter transporter, taurine), member 6	0.00	17.0	55.6	3.3	↓
1_23	gi_28461172	NM_175772	ELN	Elastin	0.00	3.2	10.2	3.2	↓
1_24	gi_119887104	XM_587930	ABCG1	Atp-binding cassette, sub-family g (white), member 1	0.00	3.5	9.6	2.7	↓
1_25	gi_31341556	NM_174602	SLC2A1	Solute carrier family 2 (facilitated glucose transporter), member 1	0.00	2.3	6.2	2.7	↓
1_26	gi_119906041	XM_584869	SDC4	Syndecan 4	0.00	5.6	14.9	2.7	---
1_27	gi_119890101	XM_616138	CACHD1	Cache domain containing 1	0.00	2.3	5.7	2.5	---
1_28	gi_114051635	NM_001046438	NRN1	Neuritin 1	0.00	12.3	29.4	2.4	---
1_29	gi_78369225	NM_001035273	SSBP4	Single stranded dna binding protein 4	0.00	5.6	13.3	2.4	---
1_30	gi_115497085	NM_001075287	CTTN	Cortactin	0.00	11.0	25.0	2.3	↓
1_31	gi_155372054	NM_001101164	EGLN3	EGL nine homolog 3 (c. Elegans)	0.00	2.6	5.8	2.2	---
1_32	gi_119919443	XM_588001	TNFRSF26	Tumor necrosis factor receptor serfamily, member 26	0.01	2.2	4.8	2.2	---

Supplementary data table 2 Full list of genes in profile 2 (n = 180)

Profile_ S. No	SEQ. ID	Acc. No. (RefSeq)	Putative Gene symbol	Gene Name	BL20 LPS Vs BL20 FDR (4h/or18)	BL20 LPS Vs BL20 Abs: FC (4h/or18)	TBL20 Vs BL20 Abs: FC	TBL20 Vs BL20 LPS Abs: FC (4h/or18)	BW720c Response
						↓	↓↓		
2_1	gi_119902260	XM_603355	LOC404176	T-cell receptor delta chain	0.00	-2.7	-238.3	-88.0	↑
2_2	gi_119913552	XM_606794	IGF1R	Insulin-like growth factor 1 receptor	0.01	-2.0	-171.5	-85.2	---
2_3	gi_119925613	XM_583887	GXYLT2	Glucoside xylosyltransferase 2	0.00	-2.7	-222.3	-83.3	---
2_4	gi_119915839	XM_588122	F13A1	Coagulation factor xiii, a1 polypeptide	0.00	-2.3	-165.6	-71.2	---
2_5	gi_119917736	XM_609898	SORCS3	Sortilin-related vps10 domain containing receptor 3	0.00	-5.4	-364.8	-67.3	---
2_6	gi_119919107	XM_591760	SLC15A3	Solute carrier family 15, member 3	0.00	-2.4	-158.8	-66.7	↑
2_7	gi_119911249	XM_867060	LAIR1	Leukocyte-associated immunoglobulin-like receptor 1	0.00	-3.3	-174.7	-52.8	---
2_8	gi_119923075	XM_001254039	LOC786327	Hypothetical protein	0.00	-2.5	-96.9	-39.1	↑
2_9	gi_119891326	XM_590508	CPA4	Carboxypeptidase a4	0.00	-3.4	-119.2	-35.2	↑
2_10	gi_119910462	XM_592455	LOC514582	Similar to b-cell receptor cd22-b	0.00	-2.4	-82.1	-34.8	↑
2_11	gi_119889163	XM_001251523	FCRLA	Fc receptor-like a	0.00	-2.8	-80.3	-28.2	↑
2_12	gi_134085858	NM_001083409	C1QTNF1	C1q and tumor necrosis factor related protein 1	0.00	-5.4	-150.3	-27.7	↑
2_13	gi_76660614	XM_597941	CCR6	Chemokine (c-c motif) receptor 6	0.00	-3.1	-71.6	-23.2	↑
2_14	gi_126723186	NM_174198	TLR4	Toll-like receptor 4	0.00	-2.9	-61.4	-21.4	↑
2_15	gi_134085612	NM_001083388	COL18A1	Collagen, type xviii, alpha 1	0.00	-3.7	-72.5	-19.6	---
2_16	gi_119895038	XM_001256059	ZNF77	Similar to zinc finger protein 77	0.00	-2.3	-42.3	-18.2	---
2_17	gi_119893570	XM_615475	LEF1	Lymphoid enhancer-binding factor 1	0.00	-3.5	-52.4	-15.0	↑
2_18	gi_119906950	XM_001251159	PPP1R14C	Protein phosphatase 1, regulatory (inhibitor) subunit 14c	0.00	-6.3	-92.1	-14.7	---
2_19	gi_119900339	XM_614789	KANK1	Kn motif and ankyrin repeat domains 1	0.00	-3.0	-43.9	-14.6	↑
2_20	gi_119895702	XM_616288	SH3TC2	Sh3 domain and tetratricopeptide repeats 2	0.01	-2.0	-26.6	-13.1	↑
2_21	gi_119909284	XM_868620	HVCN1	Hydrogen voltage-gated channel 1	0.00	-2.3	-30.4	-13.0	↑
2_22	gi_149642838	NM_001099200	PNPLA1	Patatin-like phospholipase domain containing 1	0.00	-7.5	-93.7	-12.5	---
2_23	gi_119910899	XM_001254077	LOC786386	Hypothetical protein	0.00	-2.4	-28.7	-12.0	---
2_24	gi_119911051	XM_599883	CACNG6	Calcium channel, voltage-dependent, gamma subunit 6	0.00	-2.6	-27.8	-10.6	---
2_25	gi_119919031	XM_869090	VSIG2	Similar to v-set and immunoglobulin domain containing 2	0.00	-2.3	-21.9	-9.3	---
2_26	gi_119916419	XM_587341	ALPK2	Alpha-kinase 2	0.00	-11.3	-104.0	-9.2	---
2_27	gi_119911988	XM_582998	SPECC1	Sperm antigen with calponin homology and coiled-coil domains 1	0.00	-2.8	-24.4	-8.8	↑
2_28	gi_119920600	XM_001249543	LOC781191	Hypothetical protein	0.01	-2.0	-17.7	-8.7	↑
2_29	gi_115496172	NM_001075149	MYL4	Myosin, light chain 4, alkali; atrial, embryonic	0.00	-6.9	-58.7	-8.6	↑
2_30	gi_156120728	NM_001102041	C13orf18	Chromosome 13 open reading frame 18	0.01	-2.1	-17.8	-8.6	↑
2_31	gi_119913257	XM_616682	SPEF2	Sperm flagellar 2	0.00	-2.5	-20.8	-8.2	---

2_32	gi_119920598	XM_001249501	LOC781114	Hypothetical protein	0.02	-2.0	-16.3	-8.2	---
2_33	gi_31341796	NM_174511	CCL3	Chemokine (c-c motif) ligand 3	0.00	-3.7	-29.2	-7.8	↑
2_34	gi_119889183	XR_028030	LOC517126	Similar to fc receptor-like 5	0.00	-3.9	-30.0	-7.8	---
2_35	gi_76672950	XM_607792	GAB2	Grb2-associated binding protein 2	0.00	-2.6	-20.1	-7.8	↑
2_36	gi_115496605	NM_001076352	LMO2	Lim domain only 2 (rhombotin-like 1)	0.01	-2.0	-15.7	-7.7	↑
2_37	gi_119912910	XM_599356	FAM20A	Family with sequence similarity 20, member a	0.00	-2.8	-21.3	-7.7	---
2_38	gi_156120738	NM_001102046	POU6F2	Pou class 6 homeobox 2	0.00	-2.9	-22.4	-7.7	---
2_39	gi_119918583	XR_027958	LOC531034	Similar to synaptopodin 2-like	0.00	-2.3	-17.6	-7.6	↑
2_40	gi_119905537	XM_001253283	LOC785870	Similar to prodynorphin	0.00	-2.8	-20.3	-7.3	---
2_41	gi_156523111	NM_001102500	MMRN1	Multimerin 1	0.00	-3.1	-21.8	-7.0	↑
2_42	gi_31342484	NM_174258	Cald1	Caldesmon 1	0.00	-4.6	-31.7	-6.9	---
2_43	gi_119910079	XM_592529	LPCAT2	Lysophosphatidylcholine acyltransferase 2	0.01	-2.2	-14.7	-6.8	↑
2_44	gi_78050050	NM_001035070	ASB14	Ankyrin repeat and socs box-containing 14	0.00	-2.2	-14.8	-6.8	↑
2_45	gi_119924763	XM_601339	PSD2	Pleckstrin and sec7 domain containing 2	0.00	-5.6	-37.8	-6.8	---
2_46	gi_119891244	XR_028414	C7orf58	Chromosome 7 open reading frame 58	0.00	-3.2	-21.5	-6.8	↑
2_47	gi_119893188	XM_608437	LOC529975	Hypothetical protein	0.00	-2.6	-17.0	-6.6	---
2_48	gi_115495262	NM_001075943	PEX5L	Peroxisomal biogenesis factor 5-like	0.00	-2.3	-15.2	-6.5	---
2_49	gi_119909623	XM_871314	LOC404062	Immunoglobin light chain vj region	0.00	-4.8	-30.8	-6.4	---
2_50	gi_118150919	NM_001077905	CPA5	Carboxypeptidase a5	0.00	-4.6	-28.2	-6.1	---
2_51	gi_119920009	XM_601830	NXF3	Nuclear rna export factor 3	0.00	-27.3	-166.5	-6.1	---
2_52	gi_156121314	NM_001102335	PGM5	Phosphoglucomutase 5	0.00	-8.5	-50.8	-6.0	↑
2_53	gi_115496048	NM_001075977	GMPR	Guanosine monophosphate reductase	0.00	-3.0	-17.7	-6.0	---
2_54	gi_32189337	NM_174143	PIGR	Polymeric immunoglobulin receptor	0.00	-6.4	-38.3	-5.9	↑
2_55	gi_119902288	XM_001253077	RASGRP1	Ras guanyl releasing protein 1 (calcium and dag-regulated)	0.00	-2.4	-14.3	-5.9	↑
2_56	gi_119900832	XM_001249686	LOC781282	Similar to polydom	0.00	-3.2	-19.1	-5.9	---
2_57	gi_148238272	NM_001098016	ITIH2	Inter-alpha (globulin) inhibitor h2	0.00	-5.3	-30.8	-5.9	↑
2_58	gi_89886135	NM_001013585	S1PR1	Sphingosine-1-phosphate receptor 1	0.00	-9.4	-54.6	-5.8	↑
2_59	gi_77404230	NM_001034043	Gulo	Gulonolactone (l-) oxidase	0.00	-4.9	-27.5	-5.6	---
2_60	gi_122692374	NM_001080353	MGC148792	Similar to elafin (MGC148792)	0.00	-3.6	-19.0	-5.3	---
2_61	gi_31341725	NM_174538	FCGR1A	Fc fragment of igg, high affinity ia, receptor (CD64)	0.00	-2.5	-12.8	-5.2	↑
2_62	gi_155372098	NM_001101188	ASB16	Ankyrin repeat and socs box containing 16	0.00	-5.1	-25.2	-5.0	---
2_63	gi_156523197	NM_001102543	DUSP27	Dual specificity phosphatase 27 (putative)	0.00	-4.6	-22.7	-5.0	↑
2_64	gi_119927744	XM_869472	bTrappin-5	Trappin 5 (btrappin-5)	0.00	-5.5	-26.9	-4.9	---
2_65	gi_158519866	NM_001110070	CTSW	Cathepsin W	0.00	-9.0	-43.6	-4.8	---
2_66	gi_156120752	NM_001102053	KANK4	Kn motif and ankyrin repeat domains 4	0.00	-2.8	-13.6	-4.8	---
2_67	gi_119910848	XM_581838	LOC505540	Hypothetical protein	0.00	-5.7	-27.2	-4.8	---
2_68	gi_119894347	XM_602917	DOK7	Docking protein 7	0.00	-2.6	-12.6	-4.8	---
2_69	gi_119891299	XM_607870	LOC529423	Similar to family with sequence similarity 40, member b	0.00	-4.4	-21.0	-4.7	↑
2_70	gi_118151371	NM_001078149	GSTA3	Glutathione s-transferase alpha 3	0.00	-10.0	-46.9	-4.7	---
2_71	gi_150247093	NM_001099397	GAPT	Grb2-binding adaptor protein, transmembrane	0.00	-2.5	-11.5	-4.7	↑
2_72	gi_31343049	NM_174010	CD36	CD36 molecule (thrombospondin receptor)	0.00	-24.3	-111.0	-4.6	↑
2_73	gi_76674996	XM_603316	XKRX	Similar to membrane protein xplac	0.00	-5.9	-26.7	-4.6	---
2_74	gi_155372256	NM_001101271	FAM176B	Family with sequence similarity 176, member b	0.00	-6.6	-30.0	-4.5	↑
2_75	gi_115495674	NM_001076228	PTPRG	Protein tyrosine phosphatase, receptor type, g	0.00	-2.5	-11.4	-4.5	↑
2_76	gi_84370178	NM_001038568	GPR84	G protein-coupled receptor 84	0.00	-4.1	-18.1	-4.5	↑
2_77	gi_119903128	XM_580552	MERTK	C-mer proto-oncogene tyrosine kinase	0.00	-4.5	-20.0	-4.4	---
2_78	gi_119888204	XM_595958	DNAH7	Dynein, axonemal, heavy chain 7	0.00	-2.8	-11.9	-4.3	---
2_79	gi_156120860	NM_001102107	CALCRL	Calcitonin receptor-like	0.00	-5.4	-23.2	-4.3	---
2_80	gi_115496321	NM_001075915	POU2AF1	Pou class 2 associating factor 1	0.00	-2.4	-10.1	-4.3	---
2_81	gi_119904117	XM_605012	LCN2	Lipocalin 2	0.00	-6.1	-25.6	-4.2	---
2_82	gi_119904406	XR_028798	LOC536255	Hypothetical protein	0.00	-2.5	-10.1	-4.0	↑
2_83	gi_148540299	NM_001098474	EG667604	Predicted gene 8721	0.01	-2.2	-8.7	-3.9	↑
2_84	gi_126723450	NM_001082467	C6orf26	Chromosome 6 open reading frame 26	0.00	-2.7	-9.9	-3.7	---
2_85	gi_139948854	NM_001083706	PDGFD	Platelet derived growth factor d	0.00	-2.6	-9.5	-3.7	↑
2_86	gi_114050912	NM_001046512	TSPAN12	Tetraspanin 12	0.00	-8.2	-29.9	-3.6	↑
2_87	gi_119918570	XM_001252944	CDH23	Cadherin-related 23	0.00	-7.8	-28.3	-3.6	---
2_88	gi_115494983	NM_001076301	APOD	Apolipoprotein d	0.00	-2.8	-10.1	-3.6	↑
2_89	gi_119894846	XM_611456	ARHGEF18	Rho/rac guanine nucleotide exchange factor (GEF) 18	0.00	-3.1	-11.2	-3.6	↑
2_90	gi_157428139	NM_001105511	RNASE2	Ribonuclease, rnase a family, 2 (liver, eosinophil-derived neurotoxin)	0.00	-10.0	-34.8	-3.5	---
2_91	gi_157074113	NM_001103299	PLS1	Plastin 1	0.00	-2.8	-9.9	-3.5	---
2_92	gi_118151317	NM_001078121	LOC768255	Hypothetical protein	0.00	-2.2	-7.7	-3.4	↑
2_93	gi_119904673	XM_869410	LOC617199	Similar to atpase, aminophospholipid transporter-like, class i, type 8a, member 2	0.00	-10.3	-34.1	-3.3	---
2_94	gi_119893582	XM_599206	SGMS2	Sphingomyelin synthase 2	0.00	-2.9	-9.7	-3.3	↑
2_95	gi_119920329	XM_586812	CACNA1F	Calcium channel, voltage-dependent, l type, alpha 1f subunit	0.00	-3.2	-10.4	-3.3	↑
2_96	gi_61888881	NM_001013600	BOLA-DOB	Major histocompatibility complex, class II, do beta	0.00	-3.5	-11.6	-3.3	↑
2_97	gi_149642786	NM_001099152	LBH	Limb bud and heart development homolog (mouse)	0.00	-2.9	-9.6	-3.3	↑
2_98	gi_156120434	NM_001101893	ZNF423	Zinc finger protein 423	0.01	-2.2	-7.0	-3.2	---
2_99	gi_110347592	NM_001024505		Engulfment and cell motility 1 (ELMO1), transcript variant 2	0.01	-2.1	-6.9	-3.2	---
2_100	gi_119917116	XM_870165	SH2B2	Sh2b adaptor protein 2	0.01	-2.0	-6.5	-3.2	---
2_101	gi_31342006	NM_174436	Prkg1	Protein kinase, cgmp-dependent, type 1	0.00	-4.3	-13.7	-3.2	---
2_102	gi_119917849	XM_607507	PPAPDC1A	Phosphatidic acid phosphatase type 2 domain containing 1a	0.00	-2.9	-9.1	-3.1	↑
2_103	gi_119907018	XM_866023	C11orf52	Chromosome 11 open reading frame 52	0.00	-11.5	-35.9	-3.1	↑
2_104	gi_115496130	NM_001076499	LOC390213	Double c2-like domains, gamma, pseudogene	0.01	-2.1	-6.6	-3.1	↑
2_105	gi_119912938	XR_028796	LOC615883	Similar to slit3	0.00	-4.8	-14.9	-3.1	---
2_106	gi_119912710	XR_028608	LOC788092	Similar to beta heavy chain of outer-arm axonemal dynein atpase	0.00	-2.4	-7.3	-3.1	---
2_107	gi_118151127	NM_001078018	SEZ6	Seizure related 6 homolog (mouse)	0.00	-3.1	-9.5	-3.0	---
2_108	gi_119895258	XR_028366	LOC514894	Hypothetical protein	0.01	-2.0	-6.1	-3.0	↑
2_109	gi_156121258	NM_001102307	RGNEF	190 kda guanine nucleotide exchange factor	0.00	-5.9	-17.5	-2.9	---
2_110	gi_31342140	NM_174388	MFAP2	Microfibrillar-associated protein 2	0.00	-7.7	-22.5	-2.9	↑
2_111	gi_62751568	NM_001015669	DBC1	Deleted in bladder cancer 1	0.00	-2.2	-6.4	-2.9	↑
2_112	gi_119912178	XM_612461	ABCC3	ATP-binding cassette, sub-family c (cftr/mrp), member 3	0.00	-2.3	-6.8	-2.9	↑
2_113	gi_76638859	XM_582510	GPR81	G protein-coupled receptor 81	0.00	-9.5	-27.3	-2.9	---
2_114	gi_110347586	NM_001014879	PPP2R2B	Protein phosphatase 2, regulatory subunit b, beta	0.00	-5.2	-14.9	-2.8	---
2_115	gi_119895769	XM_617891	EBF1	Early b-cell factor 1	0.00	-3.4	-9.5	-2.8	---
2_116	gi_119913498	XM_582218	zo1	Tight junction protein 1	0.00	-4.2	-11.6	-2.8	---
2_117	gi_119915518	XM_608214	LOC529757	Similar to lymphotoxin-beta, transcript variant 1	0.00	-6.8	-18.6	-2.7	↓
2_118	gi_76638411	XM_612023	MBNL3	Muscleblind-like 3 (drosophila)	0.01	-2.1	-5.6	-2.7	↑

2_119	gi_119908015	XM_604649	IL24	Interleukin 24	0.00	-2.4	-6.6	-2.7	---
2_120	gi_156121118	NM_001102237	TMEM132A	Transmembrane protein 132a	0.00	-2.2	-5.9	-2.7	---
2_121	gi_119910909	XM_870781	KLK12	Kallikrein-related peptidase 12	0.00	-2.7	-7.2	-2.7	---
2_122	gi_119895214	XM_867687	PRR16	Proline rich 16	0.00	-2.6	-6.9	-2.7	---
2_123	gi_66792889	NM_001024553	KCND1	Potassium voltage-gated channel, shal-related subfamily, member 1	0.00	-2.3	-6.1	-2.6	↑
2_124	gi_119889656	XM_001252338	KIAA1324	KIAA1324	0.00	-2.3	-5.9	-2.6	---
2_125	gi_31342494	NM_174254	CABP1	Calcium binding protein 1	0.00	-2.3	-6.0	-2.6	---
2_126	gi_62460475	NM_001014892	C16orf5	Chromosome 16 open reading frame 5	0.01	-2.1	-5.4	-2.5	---
2_127	gi_119891365	XM_605871	AGBL3	Atp/gtp binding protein-like 3	0.01	-2.2	-5.5	-2.5	↑
2_128	gi_119931673	XM_001249309	SERINC2	Serine incorporator 2	0.00	-2.2	-5.5	-2.5	↑
2_129	gi_119895089	XM_001256303	RAPGEF6	Rap guanine nucleotide exchange factor (GEF) 6	0.00	-3.2	-7.9	-2.5	---
2_130	gi_119917978	XM_591917	C10orf92	Chromosome 10 open reading frame 92	0.00	-2.5	-6.2	-2.5	---
2_131	gi_119893204	XM_001255230	LOC788054	Similar to bcl-rambo	0.00	-2.4	-5.9	-2.5	↑
2_132	gi_119903859	XM_613774	DTNB	Dystrobrevin, beta	0.01	-2.1	-5.2	-2.5	↑
2_133	gi_119906935	XM_586071	TRPC6	Transient receptor potential cation channel, subfamily c, member 6	0.00	-2.9	-7.0	-2.5	---
2_134	gi_119891011	XM_588627	LRRN3	Leucine rich repeat neuronal 3	0.00	-4.3	-10.7	-2.5	---
2_135	gi_119913675	XM_864691	CPEB1	Cytoplasmic polyadenylation element binding protein 1	0.00	-2.2	-5.5	-2.4	---
2_136	gi_31342868	NM_174091	IL18	Interleukin 18 (interferon-gamma-inducing factor)	0.00	-4.3	-10.4	-2.4	↑
2_137	gi_119927615	XM_001253466	LOC785446	Similar to ob-cadherin-1	0.00	-23.5	-57.1	-2.4	---
2_138	gi_61675701	NM_001013402	SGCA	Sarcoglycan, alpha (50kda dystrophin-associated glycoprotein)	0.00	-3.1	-7.4	-2.4	↑
2_139	gi_119908963	XM_866831	RNF150	Ring finger protein 150	0.00	-2.9	-7.0	-2.4	↑
2_140	gi_156121234	NM_001102295	TM6SF1	Transmembrane 6 superfamily member 1	0.00	-2.2	-5.3	-2.4	---
2_141	gi_119894870	XM_612193	MYO1F	Myosin if	0.00	-3.6	-8.5	-2.4	↑
2_142	gi_77736358	NM_001034707	RGS5	Regulator of g-protein signaling 5	0.00	-3.6	-8.5	-2.4	---
2_143	gi_119894939	XM_582663	TBXA2R	Thromboxane a2 receptor	0.00	-5.8	-13.7	-2.4	↑
2_144	gi_156120626	NM_001101989	TMEM144	Transmembrane protein 144	0.00	-5.7	-13.5	-2.4	↑
2_145	gi_156120736	NM_001102045	FCRL1	Fc receptor-like 1	0.00	-2.4	-5.5	-2.3	↑
2_146	gi_119912092	XM_595012	IGF2BP1	Insulin-like growth factor 2 mrna binding protein 1	0.01	-2.1	-5.0	-2.3	---
2_147	gi_119914721	XM_601010	LOC522724	Similar to bassoon protein	0.01	-2.1	-4.9	-2.3	↑
2_148	gi_115495806	NM_001076172	MARCKSL1	Marcks-like 1	0.00	-3.3	-7.5	-2.3	---
2_149	gi_119917956	XR_028155	LOC616097	Hypothetical protein	0.00	-2.4	-5.5	-2.3	---
2_150	gi_119893202	XM_870126	CECR1	Cat eye syndrome chromosome region, candidate 1	0.00	-2.7	-6.2	-2.3	↑
2_151	gi_119920461	XM_615546	LOC541298	Similar to interleukin 1 receptor accessory protein-like 1	0.01	-2.0	-4.7	-2.3	↑
2_152	gi_119886199	XM_590382	DZIP1L	Daz interacting protein 1-like	0.00	-3.9	-8.9	-2.3	---
2_153	gi_76681354	XM_876891	LOC510087	Similar to loc508140, transcript variant 2	0.01	-2.1	-4.7	-2.2	---
2_154	gi_119912116	XM_584605	NGFR	Nerve growth factor receptor	0.00	-25.0	-55.6	-2.2	---
2_155	gi_119891420	XM_597033	SVOPL	Svop-like	0.00	-9.8	-21.7	-2.2	---
2_156	gi_119909629	XM_867430	GGT1	Gamma-glutamyltransferase 1	0.00	-2.8	-6.1	-2.2	---
2_157	gi_156120476	NM_001101914	NINJ2	Ninjurin 2	0.00	-4.0	-8.7	-2.2	---
2_158	gi_119912285	XM_870899	RAPGEFL1	Rap guanine nucleotide exchange factor (gef)-like 1	0.00	-2.4	-5.2	-2.2	---
2_159	gi_119890374	XM_594566	KLF17	Kruppel-like factor 17	0.00	-2.9	-6.4	-2.2	---
2_160	gi_99028972	NM_174195	TCN2	Transcobalamin ii	0.01	-2.1	-4.6	-2.2	↑
2_161	gi_62460541	NM_001014923	NFE2	Nuclear factor (erythroid-derived 2), 45kda	0.00	-20.7	-44.5	-2.2	---
2_162	gi_118150847	NM_001077867	OSTBETA	Organic solute transporter beta	0.00	-6.9	-14.8	-2.1	↑
2_163	gi_110626118	NM_001040495	IGFBP6	Insulin-like growth factor binding protein 6	0.00	-2.9	-6.2	-2.1	---
2_164	gi_77735824	NM_001034435	CTSK	Cathepsin k	0.00	-3.7	-7.9	-2.1	↑
2_165	gi_115495086	NM_001075767	ATP2A1	Atpase, ca^{++} transporting, cardiac muscle, fast twitch 1	0.00	-2.4	-5.1	-2.1	---
2_166	gi_122692514	NM_001080250	AMICA1	Adhesion molecule, interacts with cxadr antigen 1	0.00	-2.2	-4.7	-2.1	↑
2_167	gi_164448627	NM_001015642	CPXM	Carboxypeptidase x	0.00	-4.8	-10.1	-2.1	---
2_168	gi_154152128	NM_001100334	AFAP1L1	Actin filament associated protein 1-like 1	0.00	-3.0	-6.3	-2.1	---
2_169	gi_115496685	NM_001075325	FOLR2	Folate receptor 2 (fetal)	0.00	-2.9	-6.1	-2.1	↑
2_170	gi_155371954	NM_001101113	GPX7	Glutathione peroxidase 7	0.01	-2.1	-4.4	-2.1	---
2_171	gi_149642888	NM_001099151	LHFPL2	Lipoma hmgic fusion partner-like 2	0.00	-2.5	-5.1	-2.0	↑
2_172	gi_112817614	NM_001037478	ABCG2	ATP-binding cassette, sub-family g (white), member 2	0.00	-2.9	-6.0	-2.0	↑
2_173	gi_115495372	NM_001076284	PCBP3	Poly(rc) binding protein 3	0.00	-2.7	-5.4	-2.0	---
2_174	gi_119895827	XM_001250068	ADRA1B	Adrenergic, alpha-1b-, receptor	0.00	-2.6	-5.4	-2.0	↑
2_175	gi_134085895	NM_001083469	TMEM156	Transmembrane protein 156	0.00	-4.5	-9.2	-2.0	↑
2_176	gi_119912559	XM_586841	LOC509801	Similar to IG-beta	0.00	-2.3	-4.6	-2.0	---
2_177	gi_155371996	NM_001101135	PAQR8	Progestin and adipoq receptor family member viii	0.00	-2.5	-5.1	-2.0	---
2_178	gi_28849942	NM_174776	TAP	Tracheal antimicrobial peptide	0.01	-2.0	-4.1	-2.0	---
2_179	gi_148356245	NM_001098380	HSPB2	Heat shock 27kda protein 2	0.01	-2.1	-4.2	-2.0	---
2_180	gi_157279838	NM_001104964	ART5	Adp-ribosyltransferase 5	0.00	-7.6	-15.2	-2.0	↑

Supplementary data table 3 Full list of genes in profile 3 (n = 154)

Profile_ S. No	SEQ. ID	Acc. No. (RefSeq)	Putative Gene symbol	Gene Name	BL20 LPS Vs BL20 FDR (4h/or18)	BL20 LPS Vs BL20 Abs: FC (4h/or18) ↑	TBL20 Vs BL20 Abs: FC ↓	TBL20 Vs BL20 LPS Abs: FC (4h/or18)	BW720c Response
3_1	gi_114050822	NM_001046464	MPEG1	Macrophage expressed 1	0.00	79.8	-5.6	-449.4	↑
3_2	gi_31343308	NM_173902	CLU	Clusterin	0.00	8.9	-31.7	-280.7	---
3_3	gi_60592981	NM_001012678	BOLA-DYA	Major histocompatibility complex, class ii, dy alpha (bola-dya)	0.00	27.4	-4.8	-132.1	↑
3_4	gi_119901378	XM_611161	EPHA7	Eph receptor a7	0.00	4.6	-26.0	-120.1	---
3_5	gi_119919728	XR_028639	LOC535439	Hypothetical protein	0.00	3.4	-33.9	-115.3	↑
3_6	gi_41386773	NM_174090	IL15	Interleukin 15	0.00	5.7	-18.9	-107.3	---
3_7	gi_119904381	XM_001256288	ABO	Abo blood group (transferase a, alpha 1-3-n-acetylgalactosaminyltransferase)	0.00	2.8	-35.5	-100.0	---
3_8	gi_31340694	NM_174703	TNXB	Tenascin Xb	0.00	6.6	-11.8	-78.0	---
3_9	gi_119896111	XM_593744	C8orf80	Chromosome 8 open reading frame 80	0.02	2.0	-32.4	-65.1	↑

3_10	gi_41386684	NM_174196	THBS1	Thrombospondin 1	0.00	2.5	-25.7	-65.0	---
3_11	gi_119908116	XM_864590	RGS18	Regulator of g-protein signaling 18	0.00	29.2	-2.2	-63.3	---
3_12	gi_75832075	NM_175773	IGJ	Immunoglobulin j polypeptide	0.00	2.9	-20.3	-58.1	↑
3_13	gi_119891985	XM_596354	PLXNC1	Plexin C1	0.00	2.6	-19.4	-50.5	↑
3_14	gi_78369299	NM_001035404	SAMSN1	Sam domain, sh3 domain and nuclear localization signals 1	0.00	4.5	-10.2	-46.2	↑
3_15	gi_119910850	XM_869691	RASIP1	Ras interacting protein 1	0.00	5.4	-8.5	-46.0	↑
3_16	gi_115497721	NM_001075231	PDGFA	Platelet-derived growth factor alpha polypeptide	0.00	18.0	-2.5	-44.7	---
3_17	gi_118150845	NM_001077868	STAP1	Signal transducing adaptor family member 1	0.02	2.1	-21.2	-43.8	↑
3_18	gi_76628966	XM_588243	CD207	CD207 molecule, Langerin	0.00	3.8	-11.3	-42.9	↑
3_19	gi_119892202	XM_586498	WNT10B	Wingless-type MMTV integration site family, member 10B	0.00	10.3	-4.2	-42.6	↑
3_20	gi_62751708	NM_001015520	LASS4	Lag1 homolog, ceramide synthase 4	0.00	9.2	-4.3	-39.9	↑
3_21	gi_119879508	XM_617543	CD80	CD80 molecule	0.00	13.4	-2.8	-37.9	↑
3_22	gi_31340626	NM_174725	EPAS1	Endothelial pas domain protein 1	0.00	12.0	-2.9	-35.2	↑
3_23	gi_150247135	NM_001099374	CD200R1	CD200 receptor 1	0.00	8.3	-4.2	-34.6	---
3_24	gi_76668819	XM_864611	SIM1	Single-minded homolog 1 (drosophila)	0.00	6.7	-4.9	-33.2	↑
3_25	gi_115494923	NM_001075952	ASGR2	Asialoglycoprotein receptor 2	0.00	3.4	-9.7	-32.7	↑
3_26	gi_119894218	XM_608086	LOC529634	Similar to mist	0.00	3.4	-9.5	-32.2	↑
3_27	gi_119904441	XM_580490	KBTBD6	Kelch repeat and btb (poz) domain containing 6	0.00	10.3	-3.0	-30.4	---
3_28	gi_119916402	XM_580886	CCDC68	Coiled-coil domain containing 68	0.00	5.7	-5.0	-28.1	↑
3_29	gi_94966920	NM_001040563	HTATIP2	Hiv-1 tat interactive protein 2, 30kda	0.00	6.5	-4.3	-27.9	---
3_30	gi_149642680	NM_001098991	FGR	Gardner-rasheed feline sarcoma viral (v-fgr) oncogene homolog	0.00	3.9	-7.1	-27.6	↑
3_31	gi_78369443	NM_001035293	BIRC3	Baculoviral iap repeat containing 3	0.00	5.8	-4.5	-26.1	---
3_32	gi_119910918	XM_869891	SIGLEC10	Sialic acid binding ig-like lectin 10	0.00	2.7	-9.4	-25.4	---
3_33	gi_77736010	NM_001034532	PYROXD2	Pyridine nucleotide-disulphide oxidoreductase domain 2	0.00	7.2	-3.2	-23.4	---
3_34	gi_119915684	XM_863936	UBD	Ubiquitin d	0.00	9.6	-2.4	-22.8	↑
3_35	gi_89886136	NM_001014870	TMEM106A	Transmembrane protein 106a	0.00	2.1	-10.5	-22.2	↑
3_36	gi_119934649	XM_001256269	LOC789535	Similar to vasoactive intestinal peptide receptor 2	0.00	2.4	-8.9	-21.5	---
3_37	gi_119901653	XM_604128	PLEKHG1	Pleckstrin homology domain containing, family g (with RHOGEF domain) member 1	0.00	2.1	-10.3	-21.5	---
3_38	gi_41386777	NM_174115	MUC1	Mucin 1, cell surface associated	0.00	10.2	-2.1	-21.2	---
3_39	gi_119903737	XM_596854	PLEK	Pleckstrin	0.00	5.6	-3.7	-20.3	↑
3_40	gi_162287249	NM_001075475	ADAM19	Adam metallopeptidase domain 19	0.00	3.0	-6.6	-19.8	---
3_41	gi_119890722	XM_592017	SAMD9	Sterile alpha motif domain containing 9	0.00	7.2	-2.8	-19.8	↑
3_42	gi_119904782	XM_868438	KCTD12	Potassium channel tetramerisation domain containing 12	0.00	5.7	-3.4	-19.5	↑
3_43	gi_119906769	XM_001250623	TP53INP1	Tumor protein p53 inducible nuclear protein 1	0.00	2.3	-8.3	-18.9	↑
3_44	gi_119887080	XM_584543	ICOSLG	Inducible t-cell co-stimulator ligand	0.00	3.8	-4.9	-18.9	↑
3_45	gi_119905627	XM_606839	NTSR1	Neurotensin receptor 1 (high affinity)	0.00	6.6	-2.8	-18.1	---
3_46	gi_156120748	NM_001102051	TXLNB	Taxilin beta	0.00	8.5	-2.0	-17.3	---
3_47	gi_119906824	XM_001251675	LOC784120	Hypothetical protein	0.00	3.5	-4.9	-17.3	↑
3_48	gi_119920140	XM_001251153	LOC782514	Hypothetical protein	0.00	8.3	-2.1	-17.1	---
3_49	gi_77735736	NM_001034391	LRRC33	Leucine rich repeat containing 33	0.00	2.2	-7.7	-16.7	↑
3_50	gi_119906373	XM_594631	KLHL38	Kelch-like 38 (drosophila)	0.00	4.5	-3.7	-16.7	↑
3_51	gi_139948810	NM_001083735	TRIM2	Tripartite motif containing 2	0.00	5.6	-3.0	-16.6	---
3_52	gi_119909414	XM_590469	RASAL1	Ras protein activator like 1 (gap1 like)	0.00	8.1	-2.0	-16.5	---
3_53	gi_119918508	XR_027379	MYPN	Myopalladin	0.00	3.5	-4.7	-16.4	---
3_54	gi_119887502	XR_027685	LOC540561	Similar to titin (connectin) (rhabdomyosarcoma antigen mu-rms-40.14)	0.02	2.1	-7.6	-16.2	↑
3_55	gi_119893129	XM_867752	PRMT8	Protein arginine methyltransferase 8	0.00	6.7	-2.4	-16.1	---
3_56	gi_119887408	XR_028509	LOC787276	Hypothetical protein	0.00	2.4	-6.6	-15.9	---
3_57	gi_60592983	NM_001012679	BOLA-DYB	Major histocompatibility complex, class ii, dy beta (bola-dyb)	0.00	5.9	-2.7	-15.7	---
3_58	gi_116004198	NM_001076987	PLAC8	Placenta-specific 8	0.00	3.4	-4.5	-15.3	↑
3_59	gi_119879546	XM_001251104	PARP14	Poly (adp-ribose) polymerase family, member 14	0.00	3.2	-4.6	-14.6	↑
3_60	gi_148236122	NM_001098043	CLMP	Cxadr-like membrane protein	0.00	4.5	-3.2	-14.5	---
3_61	gi_114050810	NM_001046507	EAF2	Ell associated factor 2	0.00	3.7	-3.8	-14.1	---
3_62	gi_119927163	XM_001249585	LOC783074	Hypothetical protein	0.00	3.8	-3.6	-13.7	↑
3_63	gi_156121166	NM_001102261	GBP4	Guanylate binding protein 4	0.00	2.8	-4.8	-13.5	↑
3_64	gi_125991949	NM_001081581	ABAT	4-aminobutyrate aminotransferase	0.00	6.3	-2.1	-13.5	---
3_65	gi_114052742	NM_001045877	BMP4	Bone morphogenetic protein 4	0.00	3.0	-4.2	-12.4	---
3_66	gi_119910364	XM_583222	KIRREL2	Kin of irre like 2 (drosophila)	0.00	3.4	-3.4	-11.9	---
3_67	gi_119907584	XM_867197	FCHSD2	Fch and double sh3 domains 2	0.00	2.9	-4.1	-11.8	↑
3_68	gi_84370132	NM_001038554	ADPRH	Adp-ribosylarginine hydrolase	0.00	2.1	-5.6	-11.8	↑
3_69	gi_76642926	XM_600064	SLFN11	Schlafen family member 11	0.00	2.9	-4.0	-11.8	↑
3_70	gi_156121204	NM_001102280	GAS7	Growth arrest-specific 7	0.00	2.1	-5.6	-11.6	---
3_71	gi_157279976	NM_001105034	GIMAP7	Gtpase, imap family member 7	0.00	4.4	-2.6	-11.4	---
3_72	gi_119913163	XM_001253420	LOC785366	Similar to embigin homolog (mouse)	0.01	2.2	-5.2	-11.3	↑
3_73	gi_119917968	XM_866446	JAKMIP3	Janus kinase and microtubule interacting protein 3	0.02	2.1	-5.3	-10.8	↑
3_74	gi_62751902	NM_001015610	PLA1A	Phospholipase a1 member a	0.00	5.1	-2.1	-10.7	---
3_75	gi_75832127	NM_001025326	SRGN	Serglycin (srgn)	0.00	4.6	-2.3	-10.6	---
3_76	gi_45430044	NM_205812	PKIG	Protein kinase (camp-dependent, catalytic) inhibitor gamma	0.00	4.2	-2.5	-10.6	---
3_77	gi_115496411	NM_001075349	CHN1	Chimerin (chimaerin) 1	0.00	3.2	-3.3	-10.6	↑
3_78	gi_155371902	NM_001101086	CPD	Carboxypeptidase d	0.00	3.8	-2.8	-10.5	---
3_79	gi_119921604	XM_001250189	GBP2	Guanylate binding protein 2, interferon-inducible	0.01	2.1	-5.1	-10.5	↑
3_80	gi_31341685	NM_174553	IFNAR2	Interferon (alpha, beta and omega) receptor 2	0.00	4.6	-2.3	-10.5	---
3_81	gi_114050846	NM_001046461	TRIM6-TRIM34	Trim6-trim34 readthrough	0.00	2.1	-4.9	-10.4	↑
3_82	gi_119911048	XM_602125	PRKCG	Protein kinase c, gamma	0.00	2.9	-3.6	-10.4	---
3_83	gi_114050784	NM_001046002	CD48	CD48 molecule	0.00	4.2	-2.5	-10.3	↑
3_84	gi_61870087	XM_588576	PCDHGA5	Protocadherin gamma subfamily a, 5	0.01	2.2	-4.7	-10.1	↑
3_85	gi_156120712	NM_001102033	SGK1	Serum/glucocorticoid regulated kinase 1	0.00	4.3	-2.3	-9.9	---
3_86	gi_115496777	NM_001076371	SEPT5	Septin 5	0.00	2.6	-3.9	-9.9	↑
3_87	gi_119911648	XM_001251943	LOC783305	Similar to tnf receptor-associated factor 4	0.00	3.7	-2.6	-9.8	---
3_88	gi_119894894	XM_600015	TNFSF9	Tumor necrosis factor (ligand) superfamily, member 9	0.00	2.3	-4.1	-9.7	---
3_89	gi_119888895	XM_581276	UBXN10	Ubx domain protein 10	0.00	2.5	-3.9	-9.6	---
3_90	gi_156120916	NM_001102135	LAMP3	Lysosomal-associated membrane protein 3	0.00	2.8	-3.4	-9.4	↑
3_91	gi_119914719	XM_001254179	CDHR4	Cadherin-related family member 4	0.00	2.4	-3.8	-9.3	---
3_92	gi_31342628	NM_174190	SVIL	Supervillin	0.00	4.1	-2.3	-9.2	---
3_93	gi_139949110	NM_001083769	IRF8	Interferon regulatory factor 8	0.00	2.4	-3.7	-8.8	↓
3_94	gi_119925221	XM_001254480	LOC614260	Similar to zinc finger protein 75	0.00	3.1	-2.8	-8.7	---
3_95	gi_31343026	NM_174020	LYST	Lysosomal trafficking regulator	0.00	2.2	-3.8	-8.6	---

3_96	gi_164450480	NM_001113277	KNG1	Kininogen 1	0.01	2.1	-4.1	-8.4	↑
3_97	gi_115495496	NM_001076276	MARCKS	Myristoylated alanine-rich protein kinase c substrate	0.00	3.8	-2.2	-8.3	---
3_98	gi_119889728	XM_001254754	LOC787315	Similar to vav 3 oncogene	0.00	2.3	-3.6	-8.3	---
3_99	gi_119915696	XM_870260	BTN2A2	Butyrophilin, subfamily 2, member a2	0.00	3.2	-2.5	-8.2	---
3_100	gi_156120442	NM_001101897	KIAA0226	KIAA0226	0.00	3.0	-2.7	-8.2	---
3_101	gi_119909598	XM_001256017	LOC789191	Similar to mgc127066 protein	0.00	3.6	-2.2	-8.0	---
3_102	gi_116004060	NM_001076918	TLR10	Toll-like receptor 10	0.00	3.5	-2.3	-8.0	---
3_103	gi_119891152	XM_594668	MYO1G	Myosin IG	0.00	2.6	-3.0	-8.0	---
3_104	gi_119895312	XM_866364	OR2G3	Olfactory receptor, family 2, subfamily g, member 3	0.00	3.5	-2.2	-7.8	---
3_105	gi_76678805	XM_586049	LOC509148	Similar to clll7 protein	0.00	3.1	-2.5	-7.7	---
3_106	gi_115496207	NM_001075746	GBP5	Guanylate binding protein 5	0.00	3.4	-2.2	-7.6	---
3_107	gi_119910891	XM_870599	LOC618268	Similar to CD33L1	0.00	2.3	-3.3	-7.5	---
3_108	gi_62751495	NM_001015680	NAPEPLD	N-acyl phosphatidylethanolamine phospholipase d	0.00	2.6	-2.9	-7.5	↑
3_109	gi_115495090	NM_001075392	ANKRD37	Ankyrin repeat domain 37	0.00	2.5	-3.0	-7.5	---
3_110	gi_114052650	NM_001046309	BAMBI	BMP and activin membrane-bound inhibitor homolog (xenopus laevis)	0.00	3.5	-2.1	-7.2	---
3_111	gi_119907378	XM_864679	LOC516599	Similar to tripartite motif protein trim5, transcript variant 2	0.01	2.2	-3.2	-7.1	↑
3_112	gi_119919105	XM_001254753	GPR44	G protein-coupled receptor 44	0.02	2.0	-3.4	-6.9	---
3_113	gi_115496154	NM_001075480	C11H2orf50	Chromosome 2 open reading frame 50 ortholog	0.00	2.6	-2.6	-6.9	---
3_114	gi_155371870	NM_001101070	FAM117A	Family with sequence similarity 117, member a	0.00	2.2	-3.1	-6.8	---
3_115	gi_31343630	NM_177523	SOCS2	Suppressor of cytokine signaling 2	0.00	2.5	-2.7	-6.8	---
3_116	gi_116003948	NM_001076862	SNX29	Sorting nexin 29	0.00	3.3	-2.1	-6.7	---
3_117	gi_156121170	NM_001102263	GRAMD4	Gram domain containing 4	0.00	3.2	-2.1	-6.7	---
3_118	gi_118150825	NM_001077856	LY6G6C	Lymphocyte antigen 6 complex, locus g6c	0.01	2.1	-3.2	-6.6	---
3_119	gi_114052363	NM_001046345	IKBKE	Inhibitor of kappa light polypeptide gene enhancer in b-cells, kinase epsilon	0.01	2.1	-3.1	-6.5	---
3_120	gi_164518975	NM_001113301	SERF1B	Small edrk-rich factor 1b (centromeric)	0.00	2.5	-2.6	-6.5	---
3_121	gi_126722664	NM_001082452	PARP11	Poly (adp-ribose) polymerase family, member 11	0.00	2.5	-2.6	-6.4	↑
3_122	gi_119879516	XM_593520	ARHGAP31	Rho gtpase activating protein 31	0.00	2.3	-2.8	-6.4	---
3_123	gi_115496919	NM_001075675	FABP6	Fatty acid binding protein 6, ileal	0.00	2.5	-2.6	-6.4	---
3_124	gi_119893047	XM_613380	CD163	CD163 molecule	0.01	2.1	-3.0	-6.4	↑
3_125	gi_114053094	NM_001046256	MTMR9	Myotubularin related protein 9	0.00	2.4	-2.7	-6.3	↑
3_126	gi_119912528	XM_616376	ITGB3	Integrin, beta 3 (platelet glycoprotein iiia, antigen Cd61)	0.00	2.6	-2.5	-6.3	↑
3_127	gi_119908832	XR_027675	LOC531974	Similar to mkiaa1151 protein	0.01	2.2	-2.8	-6.1	---
3_128	gi_76659540	XM_864058	LOC613370	Similar to HMGB3 protein	0.00	3.0	-2.0	-6.0	---
3_129	gi_115495920	NM_001075138	RTN4	Reticulon 4 (RTN4), transcript variant 1	0.00	3.0	-2.0	-6.0	↑
3_130	gi_119934667	XM_001256326	LOC789628	Hypothetical protein	0.00	2.8	-2.1	-6.0	---
3_131	gi_119915594	XM_606763	LOC528343	Similar to olfactory receptor mor256-5	0.00	2.5	-2.4	-6.0	↑
3_132	gi_166159169	NM_001114080		Protocadherin gamma subfamily c, 3 (pcdhgc3), transcript variant 1	0.02	2.0	-2.9	-5.9	↑
3_133	gi_119901056	XM_610591	LOC532081	Hypothetical protein	0.00	2.4	-2.4	-5.8	↑
3_134	gi_119915604	XM_589001	LOC511626	Similar to olfactory receptor, family 2, subfamily j, member 3	0.00	2.5	-2.3	-5.8	↑
3_135	gi_77735772	NM_001034409	EEPD1	Endonuclease/exonuclease/phosphatase family domain containing 1	0.00	2.3	-2.5	-5.8	---
3_136	gi_119915602	XM_001253092	Olfr129	Olfactory receptor 129	0.01	2.4	-2.4	-5.7	↑
3_137	gi_119895959	XM_613847	RGMB	Rgm domain family, member b	0.00	2.1	-2.7	-5.6	---
3_138	gi_119905278	XM_601498	CUBN	Cubilin (intrinsic factor-cobalamin receptor)	0.00	2.7	-2.0	-5.5	---
3_139	gi_125991875	NM_001081602	STK38	Serine/threonine kinase 38	0.02	2.0	-2.7	-5.5	---
3_140	gi_119889385	XM_590074	RFX5	Regulatory factor x, 5 (influences hla class ii expression)	0.00	2.1	-2.5	-5.4	---
3_141	gi_119879413	XM_001251003	LOC782355	Hypothetical protein	0.01	2.3	-2.3	-5.3	---
3_142	gi_77736300	NM_001034678	HES1	Hairy and enhancer of split 1, (drosophila)	0.00	2.1	-2.5	-5.2	↑
3_143	gi_166157485	NM_001113762	ICAM5	Intercellular adhesion molecule 5, telencephalin	0.00	2.5	-2.1	-5.2	---
3_144	gi_119903011	XM_580522	RPS6KA5	Ribosomal protein s6 kinase, 90kda, polypeptide 5	0.01	2.0	-2.6	-5.2	---
3_145	gi_119902242	XM_001253748	Olfr49	Olfactory receptor 49	0.02	2.1	-2.5	-5.2	---
3_146	gi_119934357	XM_001254441	LOC786885	Similar to glucose transporter 5	0.00	2.3	-2.2	-5.1	↑
3_147	gi_119934023	XM_001257056	LOC790637	Hypothetical protein	0.00	2.3	-2.2	-5.1	---
3_148	gi_156121152	NM_001102254	NCOA7	Nuclear receptor coactivator 7	0.00	2.1	-2.4	-5.0	---
3_149	gi_157073983	NM_001103231	MEF2B	Myocyte enhancer factor 2b	0.01	2.0	-2.5	-5.0	---
3_150	gi_115497499	NM_001075894	CDKN2B	Cyclin-dependent kinase inhibitor 2b (p15, inhibits cdk4)	0.01	2.1	-2.3	-4.9	---
3_151	gi_119902502	XM_601785	MYO1E	Myosin IE	0.01	2.1	-2.2	-4.7	---
3_152	gi_119911605	XM_610844	NEK8	Nima (never in mitosis gene a)- related kinase 8	0.01	2.1	-2.1	-4.5	---
3_153	gi_78369187	NM_001035478	SSBP2	Single-stranded dna binding protein 2	0.01	2.1	-2.2	-4.5	---
3_154	gi_119915568	XM_607721	LOC529277	Similar to homeostatic thymus hormone alpha	0.03	2.1	-2.1	-4.4	↑

Supplementary data table 4 Full list of genes in profile 4 (n = 55)

Profile_ S. No	SEQ. ID	Acc. No. (RefSeq)	Putative Gene symbol	Gene Name	BL20 LPS Vs BL20 FDR (4h/or18)	BL20 LPS Vs BL20 Abs: FC (4h/or18)	TBL20 Vs BL20 Abs: FC	TBL20 Vs BL20 LPS Abs: FC (4h/or18)	BW720c Response
						↓	↑		
4_1	gi_119919150	XM_587468	AHNAK	AHNAK nucleoprotein	0.01	-2.1	112.4	235.4	---
4_2	gi_119907136	XM_592427	OAF	Oaf homolog (drosophila)	0.00	-23.4	5.7	134.1	↓
4_3	gi_119895006	XM_590414	LOC512831	Similar to heterogeneous nuclear ribonucleoproteins a2/b1 (HNRNP a2 / HNRNP b1)	0.00	-2.4	44.5	106.1	↓
4_4	gi_122692346	NM_001080368	THEM4	Thioesterase superfamily member 4	0.00	-19.4	5.4	104.2	---
4_5	gi_119912853	XM_604523	LOC526163	Similar to cmrf-35 antigen	0.01	-2.1	47.4	99.0	---
4_6	gi_119902404	XM_869291	TMEM62	Transmembrane protein 62	0.00	-3.3	22.8	75.4	---
4_7	gi_148233315	NM_001098165	RNASEL	Ribonuclease l (2',5'-oligoisoadenylate synthetase-dependent)	0.00	-3.3	18.6	60.6	---
4_8	gi_31341924	NM_174464	SPARC	Secreted protein, acidic, cysteine-rich (osteonectin)	0.00	-14.0	3.8	53.4	---
4_9	gi_164451483	NM_001024533	SLA	Src-like-adaptor	0.00	-2.5	18.4	45.5	---
4_10	gi_78042479	NM_001035009	NDRG1	N-myc downstream regulated 1	0.00	-7.9	5.5	43.4	---
4_11	gi_119894531	XM_602953	PDE4C	Phosphodiesterase 4c, camp-specific	0.00	-2.4	17.1	41.5	↑

4_12	gi_115497115	NM_001076483	ANGPT4	Angiopoietin 4	0.00	-7.5	4.9	36.5	↓
4_13	gi_119937227	XM_001256143	LOC789368	Similar to solute carrier family 38, member 5	0.00	-9.8	3.7	36.4	---
4_14	gi_62751481	NM_001015534	GALNT6	Udp-n-acetyl-alpha-d-galactosamine:polypeptide n-acetylgalactosaminyltransferase 6	0.00	-2.6	12.4	32.8	---
4_15	gi_115495710	NM_001075904	LRRC6	Leucine rich repeat containing 6	0.00	-6.0	5.3	31.9	---
4_16	gi_119879461	XM_001253104	GCET2	Germinal center expressed transcript 2	0.00	-8.1	3.7	30.1	↑
4_17	gi_119915783	XM_617171	CMAH	Cytidine monophosphate-n-acetylneuraminic acid hydroxylase (cmp-n-acetylneuraminate monooxygenase) pseudogene	0.00	-11.0	2.5	27.7	---
4_18	gi_157279936	NM_001105014	TRIM44	Tripartite motif containing 44	0.00	-4.1	6.1	25.1	↑
4_19	gi_154707907	NM_001099095	GZMA	Granzyme a (granzyme 1, cytotoxic t-lymphocyte-associated serine esterase 3)	0.00	-4.1	6.1	24.8	---
4_20	gi_116004014	NM_001076898	PAK1	P21 protein (cdc42/rac)-activated kinase 1	0.00	-3.7	6.1	22.3	↑
4_21	gi_119888157	XM_580320	STEAP3	STEAP family member 3	0.01	-2.0	10.9	21.9	---
4_22	gi_119889474	XR_028680	LOC788729	Similar to B-cell cll/Lymphoma 9	0.00	-3.2	6.6	21.1	---
4_23	gi_119913792	XM_866155	PSTPIP1	Proline-serine-threonine phosphatase interacting protein 1	0.00	-6.4	3.2	20.7	---
4_24	gi_61316456	NM_001013003	NEIL2	Nei endonuclease viii-like 2 (e. Coli)	0.00	-2.8	7.1	20.1	---
4_25	gi_119913787	XM_616041	TBC1D2B	Tbc1 domain family, member 2b	0.00	-2.7	7.4	19.6	---
4_26	gi_119901593	XM_593801	FUCA2	Fucosidase, alpha-l- 2, plasma	0.00	-2.9	6.4	18.5	↑
4_27	gi_117935052	NM_001024930	CCR7	Chemokine (c-c motif) receptor 7	0.00	-8.9	2.0	17.9	---
4_28	gi_31341731	NM_174536	ENTPD1	Ectonucleoside triphosphate diphosphohydrolase 1	0.00	-2.3	7.6	17.1	---
4_29	gi_119879477	XM_585702	ZBTB20	Similar to btb/poz zinc finger protein DPZF	0.00	-2.3	6.7	15.1	---
4_30	gi_119896213	XM_600235	MLLT3	Myeloid/lymphoid or mixed-lineage leukemia (trithorax homolog, drosophila); translocated to, 3	0.00	-3.3	4.2	13.9	---
4_31	gi_77736587	NM_001034795	GALM	Galactose mutarotase (aldose 1-epimerase)	0.00	-6.9	2.0	13.9	---
4_32	gi_119911895	XM_866482	PIK3R5	Phosphoinositide-3-kinase, regulatory subunit 5	0.00	-2.3	6.0	13.5	---
4_33	gi_95147677	NM_001033119	FOXA3	Forkhead box a3	0.00	-2.5	4.3	10.8	---
4_34	gi_119930210	XM_001249776	LOC781367	Similar to ep2 receptor	0.01	-2.1	5.0	10.7	---
4_35	gi_119918268	XM_877825	AP3M2	Adaptor-related protein complex 3, mu 2 subunit	0.00	-2.5	4.1	10.4	---
4_36	gi_77736204	NM_001034629	EPHX1	Epoxide hydrolase 1, microsomal (xenobiotic)	0.00	-2.3	4.4	10.3	↑
4_37	gi_119906384	XM_601798	MTSS1	Metastasis suppressor 1	0.00	-2.5	3.8	9.3	↑
4_38	gi_119928343	XM_001255819	LOC788911	Similar to ectonucleotide pyrophosphatase/phosphodiesterase 2 (autotaxin)	0.00	-3.2	2.8	9.1	↓
4_39	gi_119918529	XM_585583	PRF1	Perforin 1 (pore forming protein)	0.01	-2.0	4.4	8.9	---
4_40	gi_115496985	NM_001076382	MGST2	Microsomal glutathione s-transferase 2	0.00	-3.1	2.7	8.2	---
4_41	gi_119879750	XM_596716	DGKG	Diacylglycerol kinase, gamma 90kda	0.00	-3.4	2.4	8.1	---
4_42	gi_156120702	NM_001102028	PIK3R6	Phosphoinositide-3-kinase, regulatory subunit 6	0.01	-2.0	3.8	7.8	↓
4_43	gi_134085680	NM_001083454	ABI3	ABI family, member 3	0.00	-3.0	2.5	7.6	---
4_44	gi_156121270	NM_001102313	HRC	Histidine rich calcium binding protein	0.01	-2.2	3.3	7.1	---
4_45	gi_154152162	NM_001100375	BBS12	Bardet-biedl syndrome 12	0.01	-2.1	3.3	7.0	---
4_46	gi_158937245	NM_001110194	HCK	Hemopoietic cell kinase	0.00	-2.7	2.3	6.3	---
4_47	gi_119907280	XM_001252306	SUMO3	SMT3 suppressor of mif two 3 homolog 3 (s. Cerevisiae)	0.00	-2.6	2.3	6.0	---
4_48	gi_94966784	NM_001040490	TNFRSF1B	Tumor necrosis factor receptor superfamily, member 1b	0.01	-2.1	2.8	5.9	---
4_49	gi_155372272	NM_001101279	LOC781494	Similar to myeloid-associated differentiation marker	0.01	-2.1	2.7	5.7	↑
4_50	gi_119893596	XM_584935	SLC39A8	Solute carrier family 39 (zinc transporter), member 8	0.01	-2.1	2.6	5.5	---
4_51	gi_119915318	XR_028520	LOC512304	Similar to enpp5	0.00	-2.5	2.2	5.5	---
4_52	gi_125991861	NM_001081619	MGC151839	Similar to calpain	0.01	-2.0	2.7	5.4	---
4_53	gi_116003874	NM_001076826	ANTXR2	Anthrax toxin receptor 2	0.01	-2.1	2.6	5.4	---
4_54	gi_119914706	XM_609627	SEMA3F	SEMA domain, immunoglobulin domain (ig), short basic domain, secreted, (semaphorin) 3f	0.00	-2.5	2.0	5.1	---
4_55	gi_119908660	XM_611689	LAMC1	Laminin, gamma 1 (formerly lamb2)	0.01	-2.0	2.1	4.2	---

Supplementary data table 5 Full list of genes in profile 5 (n = 38)

Profile_ S. No	SEQ. ID	Acc. No. (RefSeq)	Putative Gene symbol	Gene Name	BL20 LPS Vs BL20 FDR (4h/or18)	BL20 LPS Vs BL20 Abs: FC (4h/or18)	TBL20 Vs BL20 Abs: FC	TBL20 Vs BL20 LPS Abs: FC (4h/or18)	BW720c Response
						↑↑	↑		
5_1	gi_66792905	NM_001024557	OAS2	2'-5'-oligoadenylate synthetase 2, 69/71kda	0.00	125.9	3.9	-32.1	↑
5_2	gi_114051539	NM_001046590	CD83	CD83 molecule	0.00	57.1	2.1	-27.5	---
5_3	gi_114052748	NM_001046551	CXCL10	Chemokine (c-x-c motif) ligand 10	0.00	272.5	11.2	-24.3	↓
5_4	gi_119910676	XM_600955	RELB	V-rel reticuloendotheliosis viral oncogene homolog b	0.00	27.2	2.5	-10.7	---
5_5	gi_119901537	XM_584832	TNFAIP3	Tumor necrosis factor, alpha-induced protein 3	0.00	57.1	6.6	-8.6	---
5_6	gi_119889854	XM_001253071	TGFBR3	Transforming growth factor, beta receptor iii	0.00	37.0	4.5	-8.3	↑
5_7	gi_156523227	NM_001102558	CX3CR1	Chemokine (C-x3-C motif) receptor 1	0.00	48.5	5.9	-8.2	↑
5_8	gi_125991933	NM_001081621	TIFA	Traf-interacting protein with forkhead-associated domain	0.00	34.8	5.0	-7.0	---
5_9	gi_31343037	NM_174014	CD69	CD69 molecule	0.00	37.7	5.7	-6.6	↓
5_10	gi_119905687	XM_585095	ZBP1	Similar to putative tumor stroma and activated macrophage protein dlm-1	0.00	46.3	7.4	-6.3	↑
5_11	gi_119896106	XM_580928	DDX58	Dead (asp-glu-ala-asp) box polypeptide 58	0.00	13.9	2.2	-6.2	---
5_12	gi_62988293	NM_001017940	USP18	Ubiquitin specific peptidase 18	0.00	21.5	3.7	-5.7	---
5_13	gi_115496813	NM_001075310	BCL2L11	BCL2-like 11 (apoptosis facilitator)	0.00	14.7	2.8	-5.2	↓
5_14	gi_31343249	NM_173924	IL7	Interleukin 7	0.00	10.3	2.1	-5.0	↓
5_15	gi_27805954	NM_174366	ISG15	ISG15 ubiquitin-like modifier	0.00	120.9	25.8	-4.7	---
5_16	gi_71067099	NM_001029846	OAS1	2',5'-oligoadenylate synthetase 1, 40/46kda	0.00	13.1	2.9	-4.5	↑
5_17	gi_94967003	NM_001040606	OAS1	2',5'-oligoadenylate synthetase 1, 40/46kda	0.00	34.5	8.1	-4.3	↑
5_18	gi_62751388	NM_001015545	LGP2	Rna helicase lgp2	0.00	20.0	4.8	-4.2	↑
5_19	gi_156120848	NM_001102101	NFKB2	Nuclear factor of kappa light polypeptide gene enhancer in b-cells 2 (p49/p100)	0.00	28.3	7.1	-4.0	---
5_20	gi_115494987	NM_001075771	PARM1	Prostate androgen-regulated mucin-like protein 1	0.00	8.5	2.3	-3.7	---
5_21	gi_119894560	XM_584000	BST2	Bone marrow stromal cell antigen 2	0.00	17.6	4.8	-3.7	---
5_22	gi_119917771	XM_583612	LOC507061	Similar to dual specificity phosphatase 5	0.00	11.9	3.3	-3.6	---

5_23	gi_158341671	NM_001109981	ITGA6	Integrin, alpha 6	0.00	43.2	11.9	-3.6	---
5_24	gi_41386700	NM_174726	NFKBIZ	Nuclear factor of kappa light polypeptide gene enhancer in b-cells inhibitor, zeta	0.00	14.3	4.0	-3.6	---
5_25	gi_119913076	XM_587229	PLK2	Polo-like kinase 2	0.00	7.4	2.2	-3.4	---
5_26	gi_118150773	NM_001077827	JUN	Jun proto-oncogene	0.00	7.0	2.2	-3.1	---
5_27	gi_31342215	NM_174364	INPP1	Inositol polyphosphate-1-phosphatase	0.00	6.0	2.0	-3.0	---
5_28	gi_119908035	XM_589248	LOC511831	Similar to renin	0.00	9.4	3.3	-2.8	↑
5_29	gi_78365288	NM_001035465	IRF5	Interferon regulatory factor 5	0.00	5.9	2.1	-2.8	↑
5_30	gi_119901919	XM_593090	SMAD3	Smad family member 3	0.00	10.7	4.0	-2.7	---
5_31	gi_157074115	NM_001103300	MAFF	V-maf musculoaponeurotic fibrosarcoma oncogene homolog f (avian)	0.00	6.7	2.6	-2.6	↓
5_32	gi_119879663	XR_027887	LOC525407	Hypothetical protein	0.00	10.9	4.3	-2.6	↑
5_33	gi_31343213	NM_173941	MX2	Myxovirus (influenza virus) resistance 2 (mouse)	0.00	100.5	39.9	-2.5	↑
5_34	gi_119889992	XM_872122	IFI44	Interferon-induced protein 44	0.00	7.4	3.1	-2.4	↑
5_35	gi_118601803	NM_001079603	CBLN3	Cerebellin 3 precursor	0.00	9.3	3.9	-2.4	↑
5_36	gi_147902261	NM_001098095	EXT1	Exostosin 1	0.00	4.5	2.1	-2.2	---
5_37	gi_62988287	NM_001017937	BAIAP2	Bai1-associated protein 2	0.00	16.4	7.6	-2.2	---
5_38	gi_78045237	NM_001034502	ACTA2	Actin, alpha 2, smooth muscle, aorta	0.00	11.8	5.7	-2.1	---

Supplementary data table 6 Full list of genes in profile 6 (n = 13)

Profile_ S. No	SEQ. ID	Acc. No. (RefSeq)	Putative Gene symbol	Gene Name	BL20 LPS Vs BL20 FDR (4h/or18)	BL20 LPS Vs BL20 Abs: FC (4h/or18)	TBL20 Vs BL20 Abs: FC	TBL20 Vs BL20 LPS Abs: FC (4h/or18)	BW720c Response
						↓↓	↓		
6_1	gi_119886982	XM_001249766	RYK	RYK receptor-like tyrosine kinase	0.00	-25.8	-2.7	4.2	---
6_2	gi_150247091	NM_001099367	CYP3A4	Cytochrome p450, family 3, subfamily a, polypeptide 4	0.00	-19.4	-2.6	3.5	---
6_3	gi_115496823	NM_001076038	STXBP6	Syntaxin binding protein 6 (amisyn)	0.00	-13.0	-2.9	3.5	↑
6_4	gi_114052009	NM_001046391	CYP4F3	Cytochrome p450, family 4, subfamily f, polypeptide 3	0.00	-12.4	-3.6	3.4	---
6_5	gi_157074155	NM_001103321	GALNT4	Udp-n-acetyl-alpha-d-galactosamine:polypeptide n-acetylgalactosaminyltransferase 4 (Galnac-t4)	0.00	-11.5	-3.2	3.3	↑
6_6	gi_119911826	XR_028621	LOC528166	Similar to Nac-beta splice	0.00	-11.3	-3.6	3.2	↑
6_7	gi_31415866	NM_174573	PGLYRP1	Peptidoglycan recognition protein 1	0.00	-10.5	-2.7	3.1	---
6_8	gi_119929917	XM_001256345	LOC789663	Similar to KIAA0882 protein	0.00	-6.8	-2.2	2.4	---
6_9	gi_119904624	XM_001249768	FLT1	FMS-related tyrosine kinase 1 (vascular endothelial growth factor/vascular permeability factor receptor)	0.00	-5.7	-2.2	2.3	---
6_10	gi_119331227	NM_001079794	AQP3	Aquaporin 3 (gill blood group)	0.00	-5.6	-17.2	2.3	↑
6_11	gi_31343510	NM_177495	DNTT	Deoxynucleotidyltransferase, terminal	0.00	-5.4	-2.7	2.2	↓
6_12	gi_115496979	NM_001076384	TSPAN7	Tetraspanin 7	0.00	-5.1	-2.4	2.1	---
6_13	gi_119889935	XM_866638	LOC614782	Riken cDNA 4930503b20 gene	0.00	-5.0	-2.2	2.1	---

Supplementary data table 7 Full list of genes in profile 7 (n = 650)

Profile_ S. No	SEQ. ID	Acc. No. (RefSeq)	Putative Gene symbol	Gene Name	BL20 LPS Vs BL20 FDR (4h/or18)	BL20 LPS Vs BL20 Abs: FC (4h/or18)	TBL20 Vs BL20 Abs: FC	TBL20 Vs BL20 LPS Abs: FC (4h/or18)	BW720c Response
						↑	---		
7_1	gi_41386706	NM_174484	VCAM1	Vascular cell adhesion molecule 1	0.00	183.1	1.0	-184.6	---
7_2	gi_139947562	NM_001083739	TFEC	Transcription factor ec	0.00	34.2	-1.4	-47.1	---
7_3	gi_119913384	XM_596594	ANKRD33B	Ankyrin repeat domain 33b	0.00	30.5	-1.5	-45.1	---
7_4	gi_119924697	XM_589150	LOC511753	Similar to seven transmembrane helix receptor	0.00	35.2	-1.1	-39.7	---
7_5	gi_119922152	XM_001252866	LOC784595	Similar to olfactory receptor olfr1174	0.00	16.1	-1.7	-26.8	---
7_6	gi_119930245	XM_868109	ADAMTSL5	Adamts-like 5	0.00	15.6	-1.7	-26.6	---
7_7	gi_119911031	XM_581045	AU018091	Expressed sequence AU018091	0.00	13.5	-1.8	-24.2	---
7_8	gi_157427901	NM_001105388	ITK	Il2-inducible t-cell kinase	0.00	16.2	-1.4	-22.6	---
7_9	gi_119925538	XM_586833	LOC509794	Similar to tesc protein, transcript variant 1	0.00	16.5	-1.4	-22.5	---
7_10	gi_119892989	XM_591681	STYK1	Serine/threonine/tyrosine kinase 1	0.00	23.1	1.1	-21.4	---
7_11	gi_155372040	NM_001101158	VCAM1	Vascular cell adhesion molecule 1	0.00	14.4	-1.5	-20.9	---
7_12	gi_119892383	XM_587244	SRGAP1	Slit-robo rho gtpase activating protein 1	0.00	27.7	1.3	-20.9	---
7_13	gi_119879695	XM_867115	TP63	Tumor protein p63	0.00	36.2	1.9	-19.1	---
7_14	gi_115496711	NM_001075696	IRAK2	Interleukin-1 receptor-associated kinase 2	0.00	9.4	-2.0	-18.5	---
7_15	gi_84579864	NM_001038682	AICDA	Activation-induced cytidine deaminase	0.00	16.0	-1.1	-18.3	---
7_16	gi_119914336	XM_871109	CCR8	Chemokine (c-c motif) receptor 8	0.00	18.2	1.0	-17.7	---
7_17	gi_119934789	XM_001256599	LOC790001	Similar to transmembrane protein 17	0.00	11.0	-1.6	-17.3	---
7_18	gi_115496259	NM_001075147	CCL4	Chemokine (c-c motif) ligand 4	0.00	13.4	-1.3	-17.3	---
7_19	gi_114052247	NM_001045923	PLS3	Plastin 3	0.00	23.5	1.4	-16.5	---
7_20	gi_84000076	NM_001038050	IFI27	Interferon, alpha-inducible protein 27	0.00	14.2	-1.2	-16.5	---
7_21	gi_119927221	XM_586292	FAM111B	Family with sequence similarity 111, member b	0.00	9.8	-1.6	-15.4	---
7_22	gi_115495364	NM_001075414	IFIT3	Interferon-induced protein with tetratricopeptide repeats 3	0.00	10.4	-1.4	-14.7	---
7_23	gi_149944702	NM_001099086	BLOC1S3	Biogenesis of lysosomal organelles complex-1, subunit 3	0.00	8.2	-1.7	-14.3	---
7_24	gi_149642684	NM_001098935	OSTN	Osteocrin	0.00	10.5	-1.4	-14.2	---
7_25	gi_119906018	XM_581509	CD40	CD40 molecule, tnf receptor superfamily member 5	0.00	10.7	-1.3	-13.8	---
7_26	gi_115497163	NM_001075120	LPL	Lipoprotein lipase	0.00	7.2	-1.9	-13.8	---
7_27	gi_31343065	NM_174002	CASR	Calcium-sensing receptor	0.00	7.8	-1.7	-13.2	---

7_28	gi_114052816	NM_001045868	NFKBIA	Nuclear factor of kappa light polypeptide gene enhancer in b-cells inhibitor, alpha	0.00	6.6	-2.0	-13.0	---
7_29	gi_118150869	NM_001077879	MOBP	Myelin-associated oligodendrocyte basic protein	0.00	8.4	-1.5	-12.5	---
7_30	gi_119908270	XR_027843	LOC515996	Similar to kynurenine 3-monooxygenase	0.00	9.8	-1.3	-12.3	---
7_31	gi_119918849	XM_586751	ODZ4	Odz, odd oz/ten-m homolog 4 (drosophila)	0.00	6.9	-1.8	-12.1	---
7_32	gi_116004430	NM_001077106	MGC140080	Hypothetical protein	0.00	8.0	-1.5	-12.0	---
7_33	gi_119890779	XM_864659	AGMO	Alkylglycerol monooxygenase	0.00	5.9	-1.9	-11.2	---
7_34	gi_119907990	XM_606934	LOC528508	Similar to DTX4 protein	0.00	9.2	-1.2	-11.0	---
7_35	gi_78045490	NM_001035045	FSCN1	Fascin homolog 1, actin-bundling protein (strongylocentrotus purpuratus)	0.00	5.5	-2.0	-10.9	---
7_36	gi_84000092	NM_001038063	TMCC3	Transmembrane and coiled-coil domain family 3	0.00	16.0	1.5	-10.8	---
7_37	gi_119895319	XM_001254960	LOC787618	Similar to olfactory receptor mor104-2	0.00	8.0	-1.4	-10.8	---
7_38	gi_119926791	XM_001250097	LOC781642	Similar to olfactory receptor mor8-2	0.00	8.7	-1.2	-10.6	---
7_39	gi_156120634	NM_001101994	SLC6A12	Solute carrier family 6, member 12	0.00	9.2	-1.1	-10.4	---
7_40	gi_119924440	XM_868840	TAAR6	Trace amine associated receptor 6	0.00	7.3	-1.4	-10.1	---
7_41	gi_119910036	XR_027515	LOC539231	Hypothetical protein	0.00	11.1	1.1	-9.9	---
7_42	gi_119903170	XM_864858	TBC1D8	Tbc1 domain family, member 8 (with gram domain)	0.00	6.2	-1.6	-9.9	---
7_43	gi_31340697	NM_178572	CA2	Carbonic anhydrase ii	0.00	6.6	-1.5	-9.8	---
7_44	gi_31342632	NM_174187	SPP1	Secreted phosphoprotein 1	0.00	12.0	1.2	-9.7	---
7_45	gi_139948963	NM_001083752	C3AR1	Complement component 3a receptor 1	0.00	19.4	2.0	-9.7	---
7_46	gi_156120912	NM_001102133	FRMD6	Ferm domain containing 6	0.00	5.3	-1.8	-9.6	---
7_47	gi_59676571	NM_001012284	UBA7	Ubiquitin-like modifier activating enzyme 7	0.00	6.3	-1.5	-9.4	---
7_48	gi_119926098	XM_001254787	LOC787369	Similar to olfactory receptor Olfr1174	0.00	7.4	-1.2	-8.9	---
7_49	gi_115496899	NM_001075588	IFI6	Interferon, alpha-inducible protein 6	0.00	10.5	1.2	-8.7	---
7_50	gi_61827360	XM_586955	OR5M9	Olfactory receptor, family 5, subfamily m, member 9	0.00	8.1	-1.1	-8.7	---
7_51	gi_61826046	XM_610803	Olfr1390	Olfactory receptor 1390	0.00	6.4	-1.4	-8.6	---
7_52	gi_30794323	NM_181017	TAC3	Tachykinin 3	0.00	4.8	-1.8	-8.6	---
7_53	gi_59676558	NM_001012281	CFLAR	Casp8 and fadd-like apoptosis regulator	0.00	5.5	-1.6	-8.6	---
7_54	gi_119922313	XM_601384	Olr53	Olfactory receptor 53	0.00	7.2	-1.2	-8.5	---
7_55	gi_76681522	XM_593553	OR5D18	Olfactory receptor, family 5, subfamily d, member 18	0.00	6.3	-1.4	-8.5	---
7_56	gi_119892557	XM_001255374	Olr1012	Olfactory receptor 1012	0.00	8.5	1.0	-8.3	---
7_57	gi_157427871	NM_001105373	TMEM140	Transmembrane protein 140	0.00	5.3	-1.6	-8.3	---
7_58	gi_119895328	XM_001255032	LOC787758	Similar to olfactory receptor mor277-1	0.00	7.8	-1.0	-8.2	---
7_59	gi_119927666	XM_587987	LOC510786	Similar to olfactory receptor olfr1034	0.00	6.9	-1.2	-8.1	---
7_60	gi_155372274	NM_001101280	TRAF4	Tnf receptor-associated factor 4	0.00	4.3	-1.9	-8.1	---
7_61	gi_119914298	XR_028825	LOC509859	Similar to KIAA0342 protein	0.00	4.8	-1.7	-8.0	---
7_62	gi_76718610	XM_871454	LOC619135	Hypothetical	0.00	6.8	-1.2	-8.0	---
7_63	gi_31341586	NM_174593	RASSF2	Ras association (ralgds/af-6) domain family member 2	0.00	4.7	-1.7	-7.9	---
7_64	gi_119892545	XM_001255291	OR6C3	Olfactory receptor, family 6, subfamily c, member 3	0.00	6.3	-1.2	-7.8	---
7_65	gi_119931104	XM_001256850	LOC790355	Similar to olfactory receptor mor17-2	0.00	6.8	-1.1	-7.6	---
7_66	gi_119901479	XM_001250862	LOC783380	Similar to trace amine receptor 8	0.00	6.9	-1.1	-7.5	---
7_67	gi_119924695	XM_605606	Olfr827	Olfactory receptor 827	0.00	5.7	-1.3	-7.5	---
7_68	gi_119879545	XM_592997	DTX3L	Deltex 3-like (drosophila)	0.00	4.4	-1.7	-7.5	---
7_69	gi_76688849	XM_589845	LOC512340	Similar to olfactory receptor mor210-5	0.00	4.7	-1.5	-7.3	---
7_70	gi_166157489	NM_001113764	TYK2	Tyrosine kinase 2	0.00	3.8	-1.9	-7.3	---
7_71	gi_119921250	XM_587900	OR4A47	Olfactory receptor, family 4, subfamily a, member 47	0.00	7.3	1.0	-7.3	---
7_72	gi_119921722	XM_001255525	LOC788476	Similar to olfactory receptor olfr1174	0.00	6.4	-1.1	-7.3	---
7_73	gi_162951886	NM_001112708	SNAI1	Snail homolog 1 (drosophila)	0.00	3.8	-1.9	-7.3	---
7_74	gi_114051256	NM_001046193	ATF3	Activating transcription factor 3	0.00	3.7	-2.0	-7.3	---
7_75	gi_119887633	XM_615590	IFIH1	Interferon induced with helicase c domain 1	0.00	11.4	1.6	-7.3	---
7_76	gi_119910857	XM_587935	PLEKHA4	Pleckstrin homology domain containing, family a (phosphoinositide binding specific) member 4	0.00	7.4	1.0	-7.3	---
7_77	gi_31341381	NM_176668	AOX1	Aldehyde oxidase 1	0.00	4.1	-1.7	-7.2	---
7_78	gi_119901000	XM_613776	TRAF1	Tnf receptor-associated factor 1	0.00	3.9	-1.8	-7.2	---
7_79	gi_114051859	NM_001045963	Chn2	Chimerin (chimaerin) 2	0.00	5.3	-1.4	-7.2	---
7_80	gi_78369427	NM_001035317	MVP	Major vault protein	0.00	3.6	-2.0	-7.1	---
7_81	gi_76633796	XM_585978	C20orf123	Chromosome 20 open reading frame 123	0.00	3.8	-1.9	-7.1	---
7_82	gi_119895348	XM_001255168	OR2L3	Olfactory receptor, family 2, subfamily l, member 3	0.00	6.6	-1.1	-7.0	---
7_83	gi_119930502	XM_001255446	LOC788363	Hypothetical protein	0.00	5.8	-1.2	-7.0	---
7_84	gi_76670425	XM_588413	TAAR6	Trace amine associated receptor 6	0.00	6.0	-1.2	-7.0	---
7_85	gi_31342492	NM_178108	OAS1	2',5'-oligoadenylate synthetase 1, 40/46kda	0.00	11.2	1.6	-6.9	---
7_86	gi_119922211	XM_611011	Olfr1199	Olfactory receptor 1199	0.00	5.2	-1.3	-6.9	---
7_87	gi_119891154	XM_863839	ZMIZ2	Similar to zmiz2 protein, transcript variant 2	0.00	5.2	-1.3	-6.8	---
7_88	gi_76619274	XM_597187	OVCH1	Ovochymase 1	0.00	5.2	-1.3	-6.8	---
7_89	gi_76670295	XM_865067	RNF213	Ring finger protein 213	0.00	6.8	1.0	-6.8	---
7_90	gi_119904826	XM_001249505	SLITRK6	Slit and ntrk-like family, member 6	0.00	6.1	-1.1	-6.6	---
7_91	gi_119879459	XM_865244	C3orf52	Chromosome 3 open reading frame 52	0.00	6.6	1.0	-6.6	---
7_92	gi_118150959	NM_001077930	ERRFI1	Erbb receptor feedback inhibitor 1	0.00	6.3	1.0	-6.6	---
7_93	gi_119906065	XM_589621	ZNFX1	Similar to kiaa1404 protein	0.00	5.0	-1.3	-6.5	---
7_94	gi_114051699	NM_001046522	TMEM100	Transmembrane protein 100	0.00	7.5	1.1	-6.5	---
7_95	gi_87196509	NM_001010995	TPM2	Tropomyosin 2 (beta)	0.00	4.1	-1.6	-6.5	---
7_96	gi_115497773	NM_001075226	RILPL1	Rab interacting lysosomal protein-like 1	0.00	4.1	-1.6	-6.4	---
7_97	gi_119894058	XM_605463	ENAM	Enamelin	0.00	4.5	-1.4	-6.4	---
7_98	gi_119894556	XM_593682	LOC515629	Similar to b-cell novel protein isoform 1; bcnp1	0.00	3.9	-1.7	-6.4	---
7_99	gi_119921254	XM_609448	OR9G1	Olfactory receptor, family 9, subfamily g, member 1	0.00	5.4	-1.2	-6.4	---
7_100	gi_119902162	XM_001252369	OR11G2	Olfactory receptor, family 11, subfamily g, member 2	0.00	5.2	-1.2	-6.3	---
7_101	gi_118151335	NM_001078135	LOC768323	Hypothetical protein	0.00	6.7	1.1	-6.2	---
7_102	gi_119889733	XM_001253222	COL11A1	Collagen, type xi, alpha 1	0.00	5.2	-1.2	-6.2	---
7_103	gi_119924512	XM_001253583	Olfr1303	Olfactory receptor 1303	0.00	4.5	-1.4	-6.1	↑
7_104	gi_31342122	NM_174395	MYO1A	Myosin 1A	0.00	3.5	-1.8	-6.1	---
7_105	gi_76683585	XM_592773	Olr1159	Olfactory receptor 1159	0.00	4.2	-1.4	-6.0	---
7_106	gi_164451473	NM_001024530	SIDT2	Sid1 transmembrane family, member 2	0.00	5.6	-1.1	-6.0	---
7_107	gi_70778941	NM_001025332	FABP2	Fatty acid binding protein 2, intestinal	0.00	3.2	-1.9	-5.9	↑
7_108	gi_119915708	XM_870237	LOC617905	Hypothetical protein	0.00	4.0	-1.5	-5.9	---
7_109	gi_119915700	XM_592049	OR2M2	Olfactory receptor, family 2, subfamily m, member 2	0.00	4.6	-1.3	-5.9	---
7_110	gi_119923762	XM_001253761	OR5AN1	Olfactory receptor, family 5, subfamily an, member 1	0.00	3.7	-1.6	-5.9	---
7_111	gi_119917459	XM_580537	SLC16A12	Solute carrier family 16, member 12 (monocarboxylic acid transporter 12)	0.00	4.3	-1.4	-5.8	---
7_112	gi_119923766	XM_580276	OR5AN1	Olfactory receptor, family 5, subfamily an, member 1	0.00	3.9	-1.5	-5.8	---

7_113	gi_119887639	XM_587821	TANK	Traf family member-associated nfkb activator	0.00	4.5	-1.3	-5.7	---
7_114	gi_115497925	NM_001076102	EPS8	Epidermal growth factor receptor pathway substrate 8	0.00	6.0	1.1	-5.7	---
7_115	gi_119907935	XM_001253806	OR4A47	Olfactory receptor, family 4, subfamily a, member 47	0.00	4.8	-1.2	-5.7	---
7_116	gi_119914992	XM_583261	TXNRD3	Thioredoxin reductase 3	0.00	3.0	-1.9	-5.7	---
7_117	gi_119924693	XM_001255886	LOC788998	Similar to olfactory receptor mor210-5	0.00	4.8	-1.2	-5.7	---
7_118	gi_119892513	XM_001254983	Olfr802	Olfactory receptor 802	0.00	4.3	-1.3	-5.7	---
7_119	gi_119903539	XM_607432	LOC528994	Hypothetical protein	0.00	4.8	-1.2	-5.7	---
7_120	gi_119889996	XR_028184	LOC508347	Similar to interferon-induced protein 44-like	0.00	5.5	1.0	-5.6	---
7_121	gi_119901657	XM_595033	IPCEF1	Interaction protein for cytohesin exchange factors 1	0.00	5.0	-1.1	-5.6	---
7_122	gi_82617557	NM_205774	LOC404073	Riken cDNA 1700024p04 gene	0.00	5.0	-1.1	-5.6	---
7_123	gi_31442874	NM_174655	SLC24A1	Solute carrier family 24 (sodium/potassium/calcium exchanger), member 1	0.00	3.6	-1.5	-5.6	---
7_124	gi_119922456	XM_868862	OR8G1	Olfactory receptor, family 8, subfamily g, member 1	0.00	4.0	-1.4	-5.5	---
7_125	gi_119938335	XM_001257058	LOC790639	Similar to pol; truncated polymerase	0.00	4.3	-1.3	-5.5	---
7_126	gi_119937909	XM_001256935	LOC790478	Hypothetical protein	0.00	5.3	1.0	-5.4	---
7_127	gi_119892561	XM_001255399	OR6C74	Olfactory receptor, family 6, subfamily c, member 74	0.00	5.3	1.0	-5.4	---
7_128	gi_76685850	XM_599512	Olfr1178	Olfactory receptor 1178	0.00	5.1	-1.1	-5.4	---
7_129	gi_119926161	XM_589549	OR4L1	Olfactory receptor, family 4, subfamily l, member 1	0.00	4.6	-1.2	-5.4	---
7_130	gi_61877384	XM_591641	LOC513884	Similar to seven transmembrane helix receptor	0.00	3.9	-1.4	-5.4	---
7_131	gi_148233351	NM_001098001	FAP	Fibroblast activation protein, alpha	0.00	4.8	-1.1	-5.4	---
7_132	gi_119915305	XR_028046	RUNX2	Similar to runt-related transcription factor 2	0.00	6.3	1.2	-5.4	---
7_133	gi_31340693	NM_174701	HSD17B14	Hydroxysteroid (17-beta) dehydrogenase 14	0.00	3.0	-1.8	-5.3	---
7_134	gi_119917348	XM_583388	GPR146	G protein-coupled receptor 146	0.00	2.9	-1.9	-5.3	---
7_135	gi_118151305	NM_001078119	FBXO16	F-box protein 16	0.00	3.2	-1.6	-5.3	---
7_136	gi_119895380	XM_001255445	OR2T4	Olfactory receptor, family 2, subfamily t, member 4	0.00	4.5	-1.2	-5.3	---
7_137	gi_149642672	NM_001099071	MAP3K8	Mitogen-activated protein kinase kinase kinase 8	0.00	5.5	1.0	-5.3	---
7_138	gi_76657295	XM_869298	Olfr890	Olfactory receptor 890	0.00	4.1	-1.3	-5.3	↑
7_139	gi_157279948	NM_001105020	KRTAP9L3	Keratin associated protein 9-1	0.00	3.6	-1.5	-5.2	---
7_140	gi_76678008	XM_584199	OR10A3	Olfactory receptor, family 10, subfamily a, member 3	0.00	4.8	-1.1	-5.2	---
7_141	gi_119910279	XM_600249	DPY19L3	Dpy-19-like 3 (c. Elegans)	0.00	3.8	-1.4	-5.2	---
7_142	gi_119922019	XM_868429	OR8U8	Olfactory receptor, family 8, subfamily u, member 8	0.00	4.5	-1.1	-5.2	---
7_143	gi_119902030	XR_028230	REC8	Rec8 homolog (yeast)	0.00	3.5	-1.5	-5.2	---
7_144	gi_119931138	XM_597087	OR8G2	Olfactory receptor, family 8, subfamily g, member 2	0.00	4.9	-1.0	-5.2	---
7_145	gi_119889564	XM_001250774	CD58	CD58 molecule	0.00	2.6	-2.0	-5.2	---
7_146	gi_119892967	XM_870975	DUSP16	Dual specificity phosphatase 16	0.00	6.0	1.2	-5.1	---
7_147	gi_27806678	NM_174026	CSF1	Colony stimulating factor 1 (macrophage)	0.00	5.0	1.0	-5.1	---
7_148	gi_119915547	XM_601579	LOC523283	Similar to olfactory receptor olfr1365	0.00	4.2	-1.2	-5.1	---
7_149	gi_119918606	XM_868011	Dusp13	Dual specificity phosphatase 13 (rat)	0.00	3.8	-1.3	-5.1	---
7_150	gi_119927681	XM_001254405	LOC786840	Similar to Cfolf4	0.00	4.7	-1.1	-5.1	---
7_151	gi_119906190	XM_588232	PARP10	Poly (adp-ribose) polymerase family, member 10	0.00	2.9	-1.7	-5.0	---
7_152	gi_119892577	XM_001255552	Olr1070	Olfactory receptor 1070	0.00	4.9	1.0	-5.0	---
7_153	gi_119915596	XM_001253001	Olfr129	Olfactory receptor 129	0.00	3.1	-1.6	-5.0	---
7_154	gi_149642646	NM_001099168	RBM43	Rna binding motif protein 43	0.00	3.8	-1.3	-5.0	---
7_155	gi_119928883	XM_581609	LOC505334	Hypothetical protein	0.00	2.5	-2.0	-5.0	↑
7_156	gi_166157493	NM_001113766	TMC2	Transmembrane channel-like 2	0.00	3.2	-1.6	-5.0	---
7_157	gi_119915598	XM_588373	LOC511103	Similar to olfactory receptor, family 2, subfamily j, member 3	0.00	2.9	-1.7	-4.9	---
7_158	gi_119891937	XM_588753	ALX1	Alx homeobox 1	0.00	5.1	1.0	-4.9	---
7_159	gi_156120432	NM_001101892	C1orf109	Chromosome 1 open reading frame 109	0.00	4.3	-1.1	-4.9	---
7_160	gi_157427785	NM_001105329	CSNK1G3	Casein kinase 1, gamma 3	0.00	3.6	-1.4	-4.9	---
7_161	gi_76670917	XM_605163	LOC526789	Hypothetical protein	0.00	3.1	-1.6	-4.8	---
7_162	gi_162287311	NM_001080908	CA5B	Carbonic anhydrase vb, mitochondrial	0.00	4.1	-1.2	-4.8	---
7_163	gi_160707930	NM_001110785	IL6R	Interleukin 6 receptor	0.00	2.9	-1.7	-4.8	---
7_164	gi_119889431	XM_585905	C1orf51	Chromosome 1 open reading frame 51	0.00	3.4	-1.4	-4.8	---
7_165	gi_119923401	XM_001249461	SHISA3	Shisa homolog 3 (xenopus laevis)	0.00	4.4	-1.1	-4.8	---
7_166	gi_119892314	XM_582025	PTPRB	Protein tyrosine phosphatase, receptor type, b	0.00	4.6	-1.0	-4.8	---
7_167	gi_119912777	XM_586217	LOC509283	Similar to chromosome 17 open reading frame 27	0.00	6.4	1.3	-4.8	---
7_168	gi_119909812	XM_582583	LOC506171	Similar to phospholipase c, gamma 2	0.00	2.8	-1.7	-4.8	---
7_169	gi_119915205	XM_001250256	KCNK5	Potassium channel, subfamily k, member 5	0.00	3.1	-1.5	-4.8	---
7_170	gi_119917845	XR_027657	LOC539889	Hypothetical protein	0.00	3.4	-1.4	-4.8	---
7_171	gi_119929609	XM_611004	OR4A47	Olfactory receptor, family 4, subfamily a, member 47	0.00	4.8	1.0	-4.8	---
7_172	gi_119879472	XM_589632	ZDHHC23	Similar to membrane-associated dhhc23 zinc finger protein	0.00	5.5	1.2	-4.7	---
7_173	gi_139948994	NM_001083697	MGC157405	Pregnancy-associated glycoprotein	0.00	4.0	-1.2	-4.7	---
7_174	gi_119926223	XM_588414	OR2J2	Olfactory receptor, family 2, subfamily j, member 2	0.00	2.9	-1.6	-4.7	↑
7_175	gi_148539985	NM_174512	CDK5R1	Cyclin-dependent kinase 5, regulatory subunit 1 (p35)	0.00	2.6	-1.8	-4.7	---
7_176	gi_116003876	NM_001076828	PARP9	Poly (adp-ribose) polymerase family, member 9	0.00	4.2	-1.1	-4.7	---
7_177	gi_157427889	NM_001105382	Prune2	Prune homolog 2 (drosophila) (rat)	0.00	7.8	1.7	-4.7	---
7_178	gi_119930582	XM_001255843	Olfr591	Olfactory receptor 591	0.00	4.5	1.0	-4.7	---
7_179	gi_119925715	XM_001255793	OR5F1	Olfactory receptor, family 5, subfamily f, member 1	0.00	3.7	-1.3	-4.6	---
7_180	gi_118151377	NM_001077829	LYZ1	Lysozyme 1 (lyz1)	0.00	5.0	1.1	-4.6	---
7_181	gi_119892521	XM_001255098	Olr1012	Olfactory receptor 1012	0.00	4.5	1.0	-4.6	---
7_182	gi_119916743	XM_864316	SOCS1	Suppressor of cytokine signaling 1	0.00	8.9	2.0	-4.6	↑
7_183	gi_119922658	XM_001256086	OR5W2	Olfactory receptor, family 5, subfamily w, member 2	0.00	3.6	-1.3	-4.6	---
7_184	gi_114051785	NM_001046630	Tas2r122	Taste receptor, type 2, member 122	0.00	4.5	1.0	-4.5	---
7_185	gi_119895839	XR_028142	LOC535967	Similar to kiaa0715 protein	0.00	4.3	-1.0	-4.5	---
7_186	gi_119892689	XM_001251716	LOC783379	Similar to kiaa1033 protein	0.00	2.3	-2.0	-4.5	---
7_187	gi_115496313	NM_001075738	Gstt3	Glutathione s-transferase, theta 3	0.00	3.7	-1.2	-4.5	---
7_188	gi_119915666	XM_580689	UBD	Ubiquitin D	0.00	3.3	-1.4	-4.5	---
7_189	gi_119892596	XM_867721	ITGA7	Integrin, alpha 7	0.00	3.8	-1.2	-4.5	---
7_190	gi_119900588	XM_588491	LZTS1	Leucine zipper, putative tumor suppressor 1	0.00	2.9	-1.5	-4.5	---
7_191	gi_31343556	NM_177510	GCNT1	Glucosaminyl (n-acetyl) transferase 1, core 2	0.00	5.1	1.1	-4.4	---
7_192	gi_119901485	XM_001253189	LOC785047	Similar to trace amine receptor 8	0.00	3.7	-1.2	-4.4	---
7_193	gi_119902191	XM_609796	Olfr731	Olfactory receptor 731	0.00	4.5	1.0	-4.4	---
7_194	gi_119901577	XM_614057	GPR126	G protein-coupled receptor 126	0.00	3.3	-1.3	-4.4	---
7_195	gi_119924379	XM_001254841	OR5T1	Olfactory receptor, family 5, subfamily t, member 1	0.00	4.0	-1.1	-4.4	---
7_196	gi_119922460	XM_001250569	OR8G2	Olfactory receptor, family 8, subfamily g, member 2	0.00	3.9	-1.1	-4.4	---
7_197	gi_122692322	NM_001080336	LOC781146	Lysozyme	0.00	3.5	-1.3	-4.4	---
7_198	gi_76679710	XM_606544	PRAMEF12	Prame family member 12	0.00	3.9	-1.1	-4.4	---
7_199	gi_119926726	XM_599805	NUP210	Nucleoporin 210kda	0.00	4.3	1.0	-4.4	---
7_200	gi_119921161	XM_001254952	LOC787604	Similar to olfactory receptor olr1075	0.00	2.8	-1.5	-4.4	---
7_201	gi_119874947	XM_604589	LOC526226	Similar to histone h4	0.00	2.8	-1.5	-4.4	---

7_202	gi_116003864	NM_001076823	TAGAP	T-cell activation rhogtpase activating protein	0.00	7.0	1.6	-4.3	---
7_203	gi_119918717	XM_599400	Olfr214	Olfactory receptor 214	0.00	3.4	-1.3	-4.3	---
7_204	gi_119895410	XM_001255686	Olfr30	Olfactory receptor 30	0.00	3.7	-1.2	-4.3	---
7_205	gi_119933344	XM_001256835	Olr94	Olfactory receptor 94	0.00	3.7	-1.2	-4.3	---
7_206	gi_119879605	XM_865427	OSBPL11	Oxysterol binding protein-like 11	0.00	3.1	-1.4	-4.3	---
7_207	gi_115497767	NM_001075224	PPP1R13L	Protein phosphatase 1, regulatory (inhibitor) subunit 13 like	0.00	2.3	-1.9	-4.3	---
7_208	gi_66792729	NM_001024506	IRF9	Interferon regulatory factor 9	0.00	4.0	-1.1	-4.3	---
7_209	gi_119894006	XM_612336	UGT2B4	Udp glucuronosyltransferase 2 family, polypeptide b4	0.00	2.8	-1.5	-4.3	---
7_210	gi_119902087	XM_605073	LOC526700	Similar to olfactory receptor mor118-1	0.00	3.1	-1.4	-4.3	---
7_211	gi_119917902	XM_614294	LOC534506	Similar to KIAA0598 protein, transcript variant 1	0.00	2.9	-1.5	-4.3	---
7_212	gi_119902917	XM_869926	BATF	Basic leucine zipper transcription factor, atf-like	0.00	3.3	-1.3	-4.3	---
7_213	gi_115497501	NM_001075911	NR4A1	Nuclear receptor subfamily 4, group a, member 1	0.00	2.7	-1.6	-4.3	---
7_214	gi_114052379	NM_001046538	RAB15	Rab15, member ras onocogene family	0.00	2.5	-1.7	-4.3	---
7_215	gi_139949042	NM_001083711	TNFAIP8	Tumor necrosis factor, alpha-induced protein 8	0.00	2.4	-1.7	-4.2	---
7_216	gi_119923777	XM_001250364	LOC783509	Similar to l antigen family, member 3	0.00	2.5	-1.7	-4.2	---
7_217	gi_119924442	XM_001251569	TAAR8	Trace amine associated receptor 8	0.00	3.9	-1.1	-4.2	---
7_218	gi_76657212	XM_610318	Olfr921	Olfactory receptor 921	0.00	3.5	-1.2	-4.2	---
7_219	gi_119904452	XM_617702	DGKH	Diacylglycerol kinase, eta	0.01	2.3	-1.9	-4.2	---
7_220	gi_119879589	XM_590786	MYLK	Myosin, light polypeptide kinase (mylk)	0.00	2.6	-1.6	-4.2	---
7_221	gi_119920960	XM_001249726	OR52A5	Olfactory receptor, family 52, subfamily a, member 5	0.00	4.2	1.0	-4.2	---
7_222	gi_119894644	XM_001256245	LOC789504	Similar to bc85395_3	0.00	3.7	-1.1	-4.2	---
7_223	gi_164518983	NM_001113297	ACBD7	Acyl-coa binding domain containing 7	0.00	2.5	-1.7	-4.2	---
7_224	gi_119900621	XM_607313	LOC528877	Similar to neuron-derived orphan receptor-1 alfa	0.00	6.4	1.5	-4.2	---
7_225	gi_119902066	XM_589218	ZFHX2	Zinc finger homeobox 2	0.00	2.4	-1.7	-4.2	---
7_226	gi_119925108	XM_590561	LOC512948	Similar to olf4	0.00	4.0	-1.0	-4.2	---
7_227	gi_119902240	XM_605072	Olfr49	Olfactory receptor 49	0.01	2.3	-1.8	-4.2	---
7_228	gi_119922646	XM_867468	LOC615605	Similar to olfactory receptor mor231-1	0.00	3.5	-1.2	-4.1	---
7_229	gi_115497413	NM_001076421	SPIC	Spi-c transcription factor (Spi-1/pu.1 related)	0.00	4.5	1.1	-4.1	---
7_230	gi_119920986	XM_591949	LOC514146	Similar to olfactory receptor mor30-1	0.00	3.0	-1.4	-4.1	---
7_231	gi_156121338	NM_001102348	ARL4C	Adp-ribosylation factor-like 4c	0.00	5.6	1.4	-4.1	---
7_232	gi_119922122	XM_584568	Olfr513	Olfactory receptor 513	0.00	4.0	1.0	-4.1	---
7_233	gi_119901491	XM_865063	LOC613867	Similar to trace amine receptor 8	0.00	3.7	-1.1	-4.1	---
7_234	gi_119889139	XM_592982	OR10K1	Olfactory receptor, family 10, subfamily k, member 1	0.00	3.4	-1.2	-4.1	---
7_235	gi_119919114	XM_870538	LOC618211	Hypothetical protein	0.00	2.6	-1.6	-4.1	---
7_236	gi_119919246	XM_001254190	LOC788447	Hypothetical protein	0.00	3.6	-1.1	-4.0	---
7_237	gi_48374082	NM_001001440	ANXA4	Annexin a4	0.00	2.4	-1.7	-4.0	---
7_238	gi_119924438	XM_001251459	LOC782807	Similar to trace amine receptor 8	0.00	3.2	-1.3	-4.0	---
7_239	gi_119890020	XM_606535	WLS	Hypothetical protein	0.00	3.4	-1.2	-4.0	---
7_240	gi_139948327	NM_001083728	MAPK10	Mitogen-activated protein kinase 10	0.00	3.1	-1.3	-4.0	---
7_241	gi_119929037	XM_001250178	LOC783299	Similar to b7 olfactory receptor	0.00	4.6	1.1	-4.0	---
7_242	gi_119892563	XM_001255417	OR6C70	Olfactory receptor, family 6, subfamily c, member 70	0.00	3.7	-1.1	-4.0	---
7_243	gi_119907486	XM_580632	OR2D2	Olfactory receptor, family 2, subfamily d, member 2	0.01	2.3	-1.8	-4.0	---
7_244	gi_119912471	XM_584593	LOC539069	Similar to copper amine oxidase	0.00	3.9	1.0	-4.0	---
7_245	gi_119907337	XM_001254939	Olfr676	Olfactory receptor 676	0.00	3.0	-1.3	-4.0	---
7_246	gi_119924385	XM_583364	OR5I1	Olfactory receptor, family 5, subfamily i, member 1	0.00	4.3	1.1	-4.0	---
7_247	gi_119901487	XM_001253215	LOC785080	Similar to trace amine associated receptor 6	0.00	3.2	-1.2	-4.0	---
7_248	gi_119926100	XR_028522	LOC787388	Similar to olfactory receptor olfr1174	0.00	3.1	-1.3	-4.0	---
7_249	gi_119875275	XM_609842	BACH1	Btb and cnc homology 1, basic leucine zipper transcription factor 1	0.00	2.3	-1.7	-4.0	---
7_250	gi_119914325	XM_616073	SCN11A	Sodium channel, voltage-gated, type xi, alpha subunit	0.00	3.7	-1.1	-4.0	---
7_251	gi_119902193	XM_001252966	OR4K2	Olfactory receptor, family 4, subfamily k, member 2	0.00	4.3	1.1	-4.0	---
7_252	gi_119923020	XM_001253097	Olfr1303	Olfactory receptor 1303	0.00	4.1	1.0	-4.0	---
7_253	gi_115496034	NM_001076311	SNX24	Sorting nexin 24	0.00	6.4	1.6	-4.0	---
7_254	gi_119907606	XM_590174	Olr36	Olfactory receptor 36	0.00	3.2	-1.2	-3.9	---
7_255	gi_119901591	XM_590527	STX11	Syntaxin 11	0.00	4.5	1.1	-3.9	---
7_256	gi_119894463	XM_597995	PBX4	Pre-b-cell leukemia homeobox 4	0.00	2.7	-1.4	-3.9	---
7_257	gi_119890208	XM_588670	CC2D1B	Coiled-coil and c2 domain containing 1b	0.00	2.9	-1.3	-3.9	---
7_258	gi_119896062	XR_027676	LOC782371	Hypothetical protein	0.00	3.5	-1.1	-3.9	---
7_259	gi_119922219	XM_585077	LOC508315	Similar to olfactory receptor mor232-3	0.00	3.0	-1.3	-3.9	---
7_260	gi_119921045	XM_593866	OR52A1	Olfactory receptor, family 52, subfamily a, member 1	0.00	3.4	-1.1	-3.9	---
7_261	gi_32189319	NM_181033	LPHN3	Latrophilin 3	0.00	3.6	-1.1	-3.9	---
7_262	gi_119921882	XM_001256453	LOC789812	Similar to olfactory receptor mor262-6	0.00	3.9	1.0	-3.9	---
7_263	gi_136256478	NM_001077899	NFE2L3	Nuclear factor (erythroid-derived 2)-like 3	0.00	4.8	1.2	-3.9	---
7_264	gi_119889279	XM_001253716	NUP210L	Nucleoporin 210kda-like	0.00	2.6	-1.5	-3.9	---
7_265	gi_119892579	XM_001255560	LOC788524	Similar to cor9k3 olfactory receptor family 9 subfamily k-like	0.00	4.1	1.1	-3.9	---
7_266	gi_119906802	XM_869405	SLC26A7	Solute carrier family 26, member 7	0.01	2.1	-1.8	-3.9	---
7_267	gi_119892535	XM_001255202	OR6C1	Olfactory receptor, family 6, subfamily c, member 1	0.00	3.7	-1.1	-3.9	---
7_268	gi_119903343	XM_609234	SULT6B1	Sulfotransferase family, cytosolic, 6b, member 1	0.00	3.1	-1.3	-3.9	---
7_269	gi_119910954	XM_001254677	Vmn1r233	Similar to pheromone receptor	0.00	3.0	-1.3	-3.9	---
7_270	gi_119931962	XM_587211	GPCRLTM7	Putative olfactory receptor GPCRLTM7	0.00	4.2	1.1	-3.9	---
7_271	gi_119905969	XM_604955	TTPAL	Tocopherol (alpha) transfer protein-like	0.00	2.3	-1.7	-3.8	---
7_272	gi_119907359	XM_001255071	Olfr661	Olfactory receptor 661	0.00	3.9	1.0	-3.8	---
7_273	gi_118151441	NM_001078159	LYZ	Lysozyme	0.01	2.2	-1.7	-3.8	---
7_274	gi_119922109	XM_001250343	LOC781841	Hypothetical protein	0.00	2.4	-1.6	-3.8	---
7_275	gi_77736454	NM_001034755	MOBKL2C	Mob1, mps one binder kinase activator-like 2c (yeast)	0.00	2.6	-1.5	-3.8	---
7_276	gi_126723003	NM_001082454	CCRN4L	CCR4 carbon catabolite repression 4-like (s. Cerevisiae)	0.00	4.6	1.2	-3.8	---
7_277	gi_119921039	XM_001252349	LOC783898	Similar to mor 3beta4	0.00	3.7	1.0	-3.8	---
7_278	gi_119925825	XM_001251152	Olfr1351	Olfactory receptor 1351	0.00	2.7	-1.4	-3.8	---
7_279	gi_119895944	XM_001250359	ELL2	Elongation factor, rna polymerase ii, 2	0.00	3.9	1.0	-3.8	---
7_280	gi_119887309	XM_585327	NIPA1	Non imprinted in prader-willi/angelman syndrome 1	0.00	2.4	-1.6	-3.8	---
7_281	gi_119901496	XM_001253352	LOC785282	Similar to trace amine receptor 8	0.00	3.0	-1.2	-3.8	---
7_282	gi_119915592	XM_001252942	OR2J3	Olfactory receptor, family 2, subfamily j, member 3	0.00	2.9	-1.3	-3.8	---
7_283	gi_119916182	XM_866291	PSMA8	Proteasome (prosome, macropain) subunit, alpha type, 8	0.00	3.4	-1.1	-3.8	---
7_284	gi_76674338	XM_865987	OR7D4	Olfactory receptor, family 7, subfamily d, member 4	0.00	3.3	-1.1	-3.8	---
7_285	gi_114052944	NM_001046095	CXCL16	Chemokine (c-x-c motif) ligand 16	0.00	2.3	-1.6	-3.8	---
7_286	gi_119925385	XM_866146	LOC614592	Similar to olfactory receptor olr1414	0.00	2.7	-1.4	-3.7	---
7_287	gi_119922177	XM_001253330	LOC785259	Hypothetical protein	0.00	3.0	-1.3	-3.7	---
7_288	gi_119907937	XM_591624	OR4C13	Olfactory receptor, family 4, subfamily c, member 13	0.00	3.6	-1.1	-3.7	---
7_289	gi_119902201	XM_001253086	OR4K14	Olfactory receptor, family 4, subfamily k, member 14	0.00	3.3	-1.1	-3.7	---
7_290	gi_77404220	NM_001034056	MS4A8B	Membrane-spanning 4-domains, subfamily a, member 8b	0.00	2.3	-1.6	-3.7	---

7_291	gi_119884978	XM_001253176	GPR87	G protein-coupled receptor 87	0.00	3.6	1.0	-3.7	---
7_292	gi_119890516	XM_868290	KCNJ13	Potassium inwardly-rectifying channel, subfamily j, member 13	0.02	2.1	-1.8	-3.7	---
7_293	gi_119894665	XM_583975	LOC507378	Similar to Cfolf4	0.00	3.1	-1.2	-3.7	---
7_294	gi_119923034	XM_001253258	LOC785145	Similar to seven transmembrane helix receptor	0.00	2.3	-1.6	-3.7	---
7_295	gi_119895352	XM_591451	OR2T8	Olfactory receptor, family 2, subfamily t, member 8	0.00	3.5	-1.1	-3.7	---
7_296	gi_119908082	XM_001256153	OR5L1	Olfactory receptor, family 5, subfamily l, member 1	0.00	2.6	-1.4	-3.7	---
7_297	gi_119915994	XM_001256549	LOC789936	Similar to olfactory receptor 1160	0.00	2.5	-1.5	-3.7	---
7_298	gi_119907040	XM_590126	ZC3H12C	Similar to KIAA1726 protein, transcript variant 1	0.00	5.1	1.4	-3.6	---
7_299	gi_119910056	XM_001250846	NLRC5	Nlr family, card domain containing 5	0.00	4.3	1.2	-3.6	---
7_300	gi_119910346	XM_001253667	ZBTB32	Similar to testis zinc finger protein	0.00	2.3	-1.6	-3.6	---
7_301	gi_114703724	NM_174509	CACNB3	Calcium channel, voltage-dependent, beta 3 subunit	0.00	2.7	-1.3	-3.6	---
7_302	gi_156121078	NM_001102216	ZNF565	Zinc finger protein 565	0.00	2.4	-1.5	-3.6	---
7_303	gi_119924436	XM_001251414	TAAR8	Trace amine associated receptor 8	0.00	3.4	-1.1	-3.6	---
7_304	gi_119924775	XM_587889	LOC510712	Similar to olf4	0.00	3.4	-1.1	-3.6	---
7_305	gi_31342998	NM_174030	CTGF	Connective tissue growth factor	0.00	2.6	-1.4	-3.6	---
7_306	gi_27881411	NM_174294	CSN3	Casein kappa	0.00	2.5	-1.4	-3.6	---
7_307	gi_119921576	XM_583518	KIAA1147	KIAA1147	0.00	4.8	1.3	-3.6	---
7_308	gi_119921932	XM_871139	Olfr768	Olfactory receptor 768	0.00	2.9	-1.2	-3.6	---
7_309	gi_134085668	NM_001083451	RORC	Rar-related orphan receptor c	0.02	2.0	-1.8	-3.6	---
7_310	gi_119923914	XM_001255594	OR4A5	Olfactory receptor, family 4, subfamily a, member 5	0.00	3.1	-1.2	-3.6	---
7_311	gi_119912384	XM_586375	CCDC103	Coiled-coil domain containing 103	0.01	2.0	-1.8	-3.6	---
7_312	gi_156120686	NM_001102020	CLIP4	Cap-gly domain containing linker protein family, member 4	0.00	2.4	-1.5	-3.6	---
7_313	gi_119905750	XM_001253180	C20orf112	Chromosome 20 open reading frame 112	0.00	2.7	-1.3	-3.6	---
7_314	gi_119892451	XM_588270	STAT2	Signal transducer and activator of transcription 2, 113kda	0.00	2.9	-1.2	-3.5	---
7_315	gi_119889480	XM_868112	LOC616149	Hypothetical protein	0.01	2.3	-1.6	-3.5	---
7_316	gi_119921163	XM_869117	LOC616964	Similar to cfolf4	0.00	2.6	-1.4	-3.5	---
7_317	gi_119906071	XM_586129	B4GALT5	Udp-gal:betaglcnac beta 1,4- galactosyltransferase, polypeptide 5	0.00	6.8	1.9	-3.5	---
7_318	gi_110347578	NM_174257	S100G	S100 calcium binding protein g	0.01	2.1	-1.7	-3.5	---
7_319	gi_115496050	NM_001076208	NR4A2	Nuclear receptor subfamily 4, group a, member 2	0.00	3.4	1.0	-3.5	---
7_320	gi_156071442	NM_001101661	DEFB119	Defensin, beta 119	0.00	3.0	-1.1	-3.5	---
7_321	gi_119914146	XM_868259	RAGE	Renal tumor antigen	0.00	2.6	-1.3	-3.5	---
7_322	gi_76679265	XM_591472	OR7A10	Olfactory receptor, family 7, subfamily a, member 10	0.00	2.9	-1.2	-3.5	---
7_323	gi_119877840	XM_616959	ROBO1	Roundabout, axon guidance receptor, homolog 1 (drosophila)	0.00	2.9	-1.2	-3.5	---
7_324	gi_119877632	XM_603300	NRIP1	Nuclear receptor interacting protein 1	0.00	2.1	-1.7	-3.5	---
7_325	gi_119920994	XM_001251099	OR51T1	Olfactory receptor, family 51, subfamily t, member 1	0.00	3.5	1.0	-3.5	---
7_326	gi_164448591	NM_001113258	FHL1	Four and a half lim domains 1 (fhl1), transcript variant 1	0.00	2.5	-1.4	-3.5	---
7_327	gi_119913871	XM_617995	AKAP6	A kinase (prka) anchor protein 6	0.00	2.9	-1.2	-3.5	---
7_328	gi_119902298	XM_601356	LOC523062	Similar to seven transmembrane helix receptor	0.00	3.3	-1.1	-3.5	---
7_329	gi_76683775	XM_586650	LOC509641	Similar to olf4	0.01	2.2	-1.6	-3.5	---
7_330	gi_119919833	XM_001254041	LOC786332	Similar to transmembrane protein with egf-like and two follistatin-like domains 1	0.00	2.8	-1.3	-3.5	---
7_331	gi_119929117	XM_589033	OR4A15	Olfactory receptor, family 4, subfamily a, member 15	0.00	4.0	1.2	-3.5	---
7_332	gi_115496747	NM_001075692	MTUS1	Mitochondrial tumor suppressor 1 (mtus1), nuclear gene encoding mitochondrial protein	0.00	2.3	-1.5	-3.4	---
7_333	gi_119918970	XM_581982	OR8G5	Olfactory receptor, family 8, subfamily g, member 5	0.01	2.4	-1.5	-3.4	---
7_334	gi_119892519	XM_001255059	OR6C76	Olfactory receptor, family 6, subfamily c, member 76	0.00	3.2	-1.1	-3.4	---
7_335	gi_119892525	XM_001255131	Olfr802	Olfactory receptor 802	0.00	3.2	-1.1	-3.4	---
7_336	gi_119889486	XM_867242	NOTCH2	NOTCH 2	0.00	2.2	-1.5	-3.4	---
7_337	gi_119915359	XM_583214	NFKBIE	Nuclear factor of kappa light polypeptide gene enhancer in b-cells inhibitor, epsilon	0.00	3.9	1.2	-3.4	---
7_338	gi_119910944	XM_001254581	Vmn1r67	Similar to pheromone receptor	0.00	2.8	-1.2	-3.4	---
7_339	gi_119901769	XM_581038	MLLT4	Myeloid/lymphoid or mixed-lineage leukemia translocated to, 4	0.00	2.9	-1.2	-3.4	---
7_340	gi_119915909	XM_865312	IRF4	Interferon regulatory factor 4	0.00	4.7	1.4	-3.4	---
7_341	gi_119921525	XM_001254038	LOC786324	Similar to enterocytin	0.00	3.2	-1.1	-3.4	---
7_342	gi_119915588	XM_001252901	OR2B3	Olfactory receptor, family 2, subfamily b, member 3	0.02	2.1	-1.6	-3.4	---
7_343	gi_119920962	XM_581965	OR52A1	Olfactory receptor, family 52, subfamily a, member 1	0.00	3.3	1.0	-3.3	---
7_344	gi_119903302	XM_588244	CLEC4F	C-type lectin domain family 4, member f	0.01	2.1	-1.6	-3.3	---
7_345	gi_119892583	XM_001255583	Olr1070	Olfactory receptor 1070	0.00	2.6	-1.3	-3.3	---
7_346	gi_116004282	NM_001077032	CXorf65	Chromosome x open reading frame 65	0.01	2.2	-1.5	-3.3	---
7_347	gi_31343166	NM_173964	TEK	Tek tyrosine kinase, endothelial	0.00	2.3	-1.4	-3.3	---
7_348	gi_119908232	XM_618596	CDC42BPA	Cdc42 binding protein kinase alpha (dmpk-like)	0.00	2.4	-1.4	-3.3	---
7_349	gi_115496687	NM_001075698	IFIT5	Interferon-induced protein with tetratricopeptide repeats 5	0.00	4.1	1.2	-3.3	---
7_350	gi_66792745	NM_001024493	TRPV2	Transient receptor potential cation channel, subfamily v, member 2	0.01	2.2	-1.5	-3.3	---
7_351	gi_119910478	XM_580597	RINL	Ras and rab interactor-like	0.00	3.2	1.0	-3.3	---
7_352	gi_119912581	XR_027582	PITPNC1	Phosphatidylinositol transfer protein, cytoplasmic 1	0.00	2.3	-1.4	-3.3	---
7_353	gi_119922265	XM_001254653	LOC787179	Similar to t cell receptor alpha chain	0.00	3.4	1.0	-3.3	---
7_354	gi_78369353	NM_001035367	LOC527068	Aldo-keto reductase family 1 member c3 -like	0.01	2.2	-1.5	-3.3	---
7_355	gi_119913798	XM_588916	SNX33	Sorting nexin 33	0.00	2.2	-1.5	-3.3	---
7_356	gi_84000234	NM_001038135	REEP6	Receptor accessory protein 6	0.00	3.3	1.0	-3.3	---
7_357	gi_76625185	XM_603327	LOC524985	Similar to olfactory receptor mor222-1	0.00	3.0	-1.1	-3.3	---
7_358	gi_119889147	XM_585637	LOC508806	Similar to seven transmembrane helix receptor	0.00	2.5	-1.3	-3.3	---
7_359	gi_119916331	XM_608193	TNIP2	Tnfaip3 interacting protein 2	0.00	2.9	-1.1	-3.3	---
7_360	gi_114051342	NM_001046143	CDKN2AIP	CDKN2A interacting protein)	0.00	2.1	-1.5	-3.3	---
7_361	gi_124249271	NM_001080906	NUB1	Negative regulator of ubiquitin-like proteins 1	0.00	5.7	1.7	-3.3	---
7_362	gi_119926964	XM_594793	Olfr6	Olfactory receptor 6	0.00	3.4	1.0	-3.3	---
7_363	gi_110626110	NM_001013593	BCAT2	Branched chain amino-acid transaminase 2, mitochondrial	0.00	2.3	-1.4	-3.3	---
7_364	gi_119892555	XM_001255364	Olfr820	Olfactory receptor 820	0.00	2.7	-1.2	-3.2	---
7_365	gi_119908569	XR_028737	LOC789222	Similar to zinc finger protein riz	0.00	2.3	-1.4	-3.2	---
7_366	gi_119914504	XM_001251656	LOC783001	Similar to chromosome 12 open reading frame 2	0.00	2.4	-1.4	-3.2	---
7_367	gi_119923954	XM_595420	Olfr1276	Olfactory receptor 1276	0.00	3.1	-1.1	-3.2	---
7_368	gi_119919022	XM_001251836	OR8A1	Olfactory receptor, family 8, subfamily a, member 1	0.00	2.4	-1.3	-3.2	---
7_369	gi_119892575	XM_001255503	LOC788438	Similar to olfactory receptor mor210-4	0.00	3.0	-1.1	-3.2	---
7_370	gi_156120584	NM_001101968	STYXL1	Serine/threonine/tyrosine interacting-like 1	0.01	2.2	-1.4	-3.2	---
7_371	gi_119922882	XM_600016	LOC521749	Similar to olfactory receptor mor245-4	0.00	3.5	1.1	-3.2	---
7_372	gi_119913158	XM_588962	PARP8	Poly (adp-ribose) polymerase family, member 8	0.00	3.2	1.0	-3.2	---
7_373	gi_114050764	NM_001046472	GPR183	G protein-coupled receptor 183	0.01	2.2	-1.4	-3.2	---

7_374	gi_77736288	NM_001034672	TSPAN33	Tetraspanin 33	0.00	4.4	1.4	-3.2	---
7_375	gi_27807288	NM_174716	ANXA2	Annexin a2	0.00	3.2	1.0	-3.2	---
7_376	gi_119921731	XM_001255638	LOC788640	Similar to olfactory receptor olr87	0.00	3.3	1.0	-3.2	---
7_377	gi_119921037	XM_604679	Olfr622	Olfactory receptor 622	0.00	2.5	-1.3	-3.2	---
7_378	gi_119902174	XM_605040	LOC526667	Similar to olfactory receptor mor106-5	0.01	2.3	-1.4	-3.2	---
7_379	gi_119902413	XM_617674	MDGA2	Mam domain containing glycosylphosphatidylinositol anchor2	0.00	2.9	-1.1	-3.2	---
7_380	gi_119895360	XM_586131	LOC509217	Similar to seven transmembrane helix receptor	0.00	2.7	-1.2	-3.2	---
7_381	gi_119893497	XM_607704	NDST4	N-deacetylase/n-sulfotransferase (heparan glucosaminyl) 4	0.00	2.7	-1.2	-3.2	---
7_382	gi_156121044	NM_001102199	MYO1B	Myosin ib	0.00	2.4	-1.3	-3.2	---
7_383	gi_134085656	NM_001083422	STK17A	Serine/threonine kinase 17a	0.00	2.3	-1.4	-3.2	---
7_384	gi_119915895	XM_001250447	LOC782393	Similar to eukaryotic translation elongation factor 1 epsilon 1	0.00	2.1	-1.5	-3.2	---
7_385	gi_119888596	XR_028504	SP110	Sp110 nuclear body protein	0.00	2.3	-1.4	-3.2	---
7_386	gi_119901002	XM_589483	C5	Complement component 5	0.00	2.4	-1.3	-3.2	---
7_387	gi_119886996	XM_001249828	LCA5L	Leber congenital amaurosis 5-like	0.00	2.6	-1.2	-3.1	---
7_388	gi_119892507	XM_001254890	Olfr770	Olfactory receptor 770	0.00	2.9	-1.1	-3.1	---
7_389	gi_119924514	XM_001253602	Olfr1293	Olfactory receptor 1293	0.00	3.0	-1.1	-3.1	---
7_390	gi_134085676	NM_001083530	S100A3	S100 calcium binding protein a3	0.00	4.0	1.3	-3.1	---
7_391	gi_119926165	XM_001255258	LOC788100	Similar to seven transmembrane helix receptor	0.01	2.2	-1.4	-3.1	---
7_392	gi_157279990	NM_001105041	RUSC1	Run and sh3 domain containing 1	0.00	4.2	1.3	-3.1	---
7_393	gi_116734828	NM_001075320	TIMD4	T-cell immunoglobulin and mucin domain containing 4	0.00	2.5	-1.2	-3.1	---
7_394	gi_31341720	NM_174541	GABRA2	Gamma-aminobutyric acid (gaba) a receptor, alpha 2	0.00	2.5	-1.3	-3.1	---
7_395	gi_119892357	XM_592833	DYRK2	Dual-specificity tyrosine-(y)-phosphorylation regulated kinase 2	0.01	2.2	-1.4	-3.1	---
7_396	gi_119926000	XM_001250683	CA1	Carbonic anhydrase i	0.00	2.9	-1.1	-3.1	---
7_397	gi_119923232	XM_612459	LOC533149	Hypothetical protein	0.00	2.3	-1.4	-3.1	---
7_398	gi_119910251	XM_001250104	FTSJD1	Ftsj methyltransferase domain containing 1	0.00	5.1	1.6	-3.1	---
7_399	gi_119920696	XM_608251	CRLF2	Cytokine receptor-like factor 2	0.00	4.0	1.3	-3.1	---
7_400	gi_119907426	XM_001255664	OR52A1	Olfactory receptor, family 52, subfamily a, member 1	0.00	3.4	1.1	-3.1	---
7_401	gi_119902080	XM_588656	BVd1.18	T cell receptor delta chain variable region	0.00	2.6	-1.2	-3.1	---
7_402	gi_119888478	XM_587645	CPS1	Carbamoyl-phosphate synthase 1, mitochondrial	0.02	2.0	-1.5	-3.1	---
7_403	gi_119884953	XM_591264	PLCH1	Phospholipase c, eta 1	0.00	5.0	1.6	-3.1	---
7_404	gi_119924516	XM_001253624	LOC785683	Similar to olfactory receptor olr758	0.00	2.5	-1.2	-3.1	---
7_405	gi_119889928	XM_609742	COL24A1	Collagen, type xxiv, alpha 1	0.00	3.1	1.0	-3.1	---
7_406	gi_115497951	NM_001075871	IER2	Immediate early response 2	0.00	4.0	1.3	-3.1	---
7_407	gi_119915866	XM_607555	HIVEP1	Human immunodeficiency virus type i enhancer binding protein 1	0.00	3.8	1.2	-3.1	---
7_408	gi_119911171	XM_609549	ZNF584	Similar to zinc finger protein 420	0.01	2.1	-1.5	-3.1	---
7_409	gi_119920543	XM_868002	MAP7D2	Map7 domain containing 2	0.00	3.1	1.0	-3.1	---
7_410	gi_119893022	XM_587046	Clec2e	C-type lectin domain family 2, member e	0.00	2.5	-1.2	-3.1	---
7_411	gi_119921415	XM_001249872	LOC781446	Similar to olfactory receptor olr1108	0.00	2.9	-1.0	-3.0	---
7_412	gi_119901510	XM_001253691	TAAR1	Trace amine associated receptor 1	0.00	3.0	1.0	-3.0	---
7_413	gi_119891945	XM_001249702	MGAT4C	Mannosyl (alpha-1,3-)-glycoprotein beta-1,4-n-acetylglucosaminyltransferase, isozyme c (putative)	0.00	3.1	1.0	-3.0	---
7_414	gi_119923045	XM_001253400	LOC785338	Hypothetical protein	0.00	2.4	-1.2	-3.0	---
7_415	gi_119938379	XM_001257069	LOC790653	Similar to mll5	0.01	2.3	-1.3	-3.0	---
7_416	gi_156120930	NM_001102142	SP7	Sp7 transcription factor	0.00	2.5	-1.2	-3.0	---
7_417	gi_164448597	NM_001035271	KLF6	Kruppel-like factor 6	0.00	2.1	-1.4	-3.0	---
7_418	gi_119901506	XM_001253621	Taar3	Trace amine-associated receptor 3	0.00	2.4	-1.2	-3.0	---
7_419	gi_119895362	XM_868904	OR2M4	Olfactory receptor, family 2, subfamily m, member 4	0.00	2.8	-1.1	-3.0	---
7_420	gi_119929817	XM_593643	Olfr1086	Olfactory receptor 1086	0.00	2.5	-1.2	-3.0	---
7_421	gi_119889478	XM_608618	Olfr1402	Olfactory receptor 1402	0.00	2.8	-1.1	-3.0	---
7_422	gi_119915614	XM_001253184	LOC785040	Similar to seven transmembrane helix receptor	0.00	2.9	-1.1	-3.0	---
7_423	gi_119894688	XM_001249956	ZSWIM4	Similar to flj00044 protein	0.00	2.5	-1.2	-3.0	---
7_424	gi_119877213	XM_614756	ROBO2	Roundabout, axon guidance receptor, homolog 2 (drosophila)	0.01	2.2	-1.4	-3.0	---
7_425	gi_119916408	XM_616601	WDR7	Similar to rabconnectin-3 beta	0.00	2.2	-1.4	-3.0	---
7_426	gi_119906499	XM_588521	VCPIP1	Similar to kiaa1850 protein, transcript variant 1	0.01	2.0	-1.5	-3.0	---
7_427	gi_119922419	XM_001254973	Olfr1132	Olfactory receptor 1132	0.00	2.8	-1.1	-3.0	---
7_428	gi_119930323	XM_001253262	LOC785149	Similar to olfactory receptor olr1414	0.01	2.2	-1.4	-3.0	---
7_429	gi_119923950	XM_001250244	LOC781758	Similar to olfactory receptor olr753	0.02	2.2	-1.4	-3.0	---
7_430	gi_119889472	XM_001255499	LOC788724	Similar to hist2h2aa1 protein	0.00	2.5	-1.2	-3.0	---
7_431	gi_76662928	XM_588102	OR2M3	Olfactory receptor, family 2, subfamily m, member 3	0.00	2.9	1.0	-3.0	---
7_432	gi_119902300	XM_001254661	Olfr1276	Olfactory receptor 1276	0.00	3.0	1.0	-3.0	---
7_433	gi_119924838	XM_001253851	LOC786035	Similar to seven transmembrane helix receptor	0.00	2.7	-1.1	-3.0	---
7_434	gi_119895332	XM_001255076	OR2T8	Olfactory receptor, family 2, subfamily t, member 8	0.00	2.8	-1.1	-3.0	---
7_435	gi_116003900	NM_001076841	H2-T24	Histocompatibility 2, t region locus 24	0.00	3.1	1.0	-3.0	---
7_436	gi_77735876	NM_001034463	C21orf33	Chromosome 21 open reading frame 33	0.00	2.6	-1.1	-3.0	---
7_437	gi_119921644	XM_001254228	LOC786593	Similar to t-cell receptor alpha chain	0.00	3.0	1.0	-3.0	---
7_438	gi_119894394	XM_585315	BOD1L	Biorientation of chromosomes in cell division 1-like	0.00	2.1	-1.4	-3.0	---
7_439	gi_119915680	XM_001253103	LOC786846	Similar to olfactory receptor 9, rat orthologue of mouse mor156-2a, transcript variant 1	0.01	2.2	-1.3	-3.0	---
7_440	gi_119907926	XM_869607	OR8H3	Olfactory receptor, family 8, subfamily h, member 3	0.00	2.6	-1.1	-3.0	---
7_441	gi_119922650	XM_601354	Olfr1239	Olfactory receptor 1239	0.01	2.3	-1.3	-3.0	---
7_442	gi_119895350	XM_001255203	LOC788011	Similar to olfactory receptor olr1566	0.00	2.4	-1.2	-3.0	---
7_443	gi_119923779	XM_001250462	LOC783543	Similar to l antigen family, member 3, transcript variant 2	0.00	2.4	-1.2	-3.0	---
7_444	gi_119879601	XM_612607	KALRN	Kalirin, rhogef kinase	0.01	2.2	-1.4	-3.0	---
7_445	gi_31341372	NM_176674	COX7A1	Cytochrome c oxidase subunit viia polypeptide 1 (muscle)	0.01	2.0	-1.4	-2.9	---
7_446	gi_117935050	NM_178109	EIF2AK2	Eukaryotic translation initiation factor 2-alpha kinase 2	0.00	2.9	1.0	-2.9	---
7_447	gi_119921043	XM_001252468	OR52A5	Olfactory receptor, family 52, subfamily a, member 5	0.00	2.8	-1.0	-2.9	---
7_448	gi_139949155	NM_001083740	GPT	Glutamic-pyruvate transaminase (alanine aminotransferase)	0.00	2.4	-1.2	-2.9	---
7_449	gi_119929446	XM_001256647	LOC790072	Hypothetical protein	0.00	3.4	1.2	-2.9	---
7_450	gi_148226500	NM_001098047	GPR21	G protein-coupled receptor 21	0.01	2.4	-1.2	-2.9	---
7_451	gi_119925030	XM_001251274	LOC786338	Hypothetical protein	0.00	2.9	1.0	-2.9	---
7_452	gi_77736166	NM_001034610	RIPK2	Receptor-interacting serine-threonine kinase 2	0.00	2.6	-1.1	-2.9	---
7_453	gi_119924682	XM_581926	LOC538674	Similar to protocadherin	0.01	2.2	-1.3	-2.9	---
7_454	gi_119892509	XM_001254905	Olfr794	Olfactory receptor 794	0.01	2.2	-1.3	-2.9	---
7_455	gi_119895309	XM_601243	OR2C3	Olfactory receptor, family 2, subfamily c, member 3	0.00	2.7	-1.1	-2.9	---
7_456	gi_119892559	XM_001255392	OR6C6	Olfactory receptor, family 6, subfamily c, member 6	0.00	2.7	-1.1	-2.9	---
7_457	gi_119924200	XM_614803	GLRA3	Glycine receptor, alpha 3	0.00	2.7	-1.0	-2.9	---
7_458	gi_76662918	XM_868812	OR2L2	Olfactory receptor, family 2, subfamily l, member 2	0.01	2.3	-1.3	-2.9	---
7_459	gi_50872142	NM_001002890	KLRD1	Killer cell lectin-like receptor subfamily d, member 1	0.01	2.2	-1.3	-2.9	---
7_460	gi_119925323	XM_001255584	Olfr905	Olfactory receptor 905	0.00	2.5	-1.2	-2.9	---

7_461	gi_119927767	XM_868179	OR5M3	Olfactory receptor, family 5, subfamily m, member 3	0.00	2.6	-1.1	-2.9	---
7_462	gi_139947537	NM_001083736	JAM2	Junctional adhesion molecule 2	0.00	4.4	1.5	-2.9	---
7_463	gi_116004236	NM_001077009	P2RY14	Purinergic receptor p2y, g-protein coupled, 14	0.00	2.7	-1.1	-2.9	---
7_464	gi_119915421	XM_584616	LOC507917	MHC class I heavy chain	0.00	2.6	-1.1	-2.9	---
7_465	gi_76677494	XM_587133	OR6C4	Olfactory receptor, family 6, subfamily c, member 4	0.01	2.2	-1.3	-2.9	---
7_466	gi_119894667	XM_001256348	LOC789667	Similar to olf4	0.00	3.1	1.1	-2.9	---
7_467	gi_157427975	NM_001105426	RNASE13	Ribonuclease, rnase a family, 13 (non-active)	0.00	3.5	1.2	-2.9	---
7_468	gi_76635921	XM_596754	Olfr561	Olfactory receptor 561	0.01	2.3	-1.3	-2.9	---
7_469	gi_119906418	XM_001250149	LOC782051	Similar to alpha 2 actin	0.00	2.7	-1.0	-2.9	---
7_470	gi_119892569	XM_001255472	LOC788396	Similar to olfactory receptor mor265-1	0.00	3.1	1.1	-2.8	---
7_471	gi_119922015	XM_001253993	OR8J1	Olfactory receptor, family 8, subfamily j, member 1	0.01	2.5	-1.2	-2.8	---
7_472	gi_31341996	NM_174439	TMPRSS15	Transmembrane protease, serine 15	0.01	2.3	-1.2	-2.8	---
7_473	gi_118151099	NM_001078004	PELI1	Pellino homolog 1 (drosophila)	0.00	3.8	1.3	-2.8	---
7_474	gi_119920980	XM_001250243	OR51F2	Olfactory receptor, family 51, subfamily f, member 2	0.00	2.4	-1.2	-2.8	---
7_475	gi_119921914	XM_001250926	LOC782296	Similar to olfactory receptor mor114-3	0.00	2.7	-1.0	-2.8	---
7_476	gi_149642782	NM_001098895	SLC25A30	Solute carrier family 25, member 30	0.00	2.2	-1.3	-2.8	---
7_477	gi_119902302	XM_001254703	Olfr1276	Olfactory receptor 1276	0.01	2.2	-1.3	-2.8	---
7_478	gi_119903309	XM_605410	SRD5A2	Steroid-5-alpha-reductase, alpha polypeptide 2 (3-oxo-5 alpha-steroid delta 4-dehydrogenase alpha 2)	0.01	2.1	-1.3	-2.8	---
7_479	gi_76684604	XM_870419	Olr468	Olfactory receptor 468	0.01	2.3	-1.2	-2.8	---
7_480	gi_119907381	XM_593669	OR52H1	Olfactory receptor, family 52, subfamily h, member 1	0.01	2.3	-1.2	-2.8	---
7_481	gi_119911558	XM_588697	TNFAIP1	Tumor necrosis factor, alpha-induced protein 1 (endothelial)	0.00	2.7	-1.0	-2.8	---
7_482	gi_119892515	XM_584195	LOC507560	Similar to olfactory receptor olr920	0.01	2.4	-1.2	-2.8	---
7_483	gi_119926167	XM_001255280	LOC788129	Similar to seven transmembrane helix receptor	0.01	2.3	-1.2	-2.8	---
7_484	gi_27805856	NM_174285	CREB1	Camp responsive element binding protein 1	0.00	2.1	-1.3	-2.8	---
7_485	gi_119887404	XM_589101	FSIP2	Fibrous sheath interacting protein 2	0.00	2.4	-1.2	-2.8	---
7_486	gi_119893030	XM_584745	LOC508026	Similar to pregnancy-zone protein	0.03	2.0	-1.4	-2.8	---
7_487	gi_119894646	XM_590569	LOC512956	Similar to olfactory receptor	0.00	2.6	-1.1	-2.8	---
7_488	gi_119921997	XM_001253442	LOC785406	Similar to olfactory receptor mor185-5	0.00	2.8	1.0	-2.8	---
7_489	gi_119892531	XM_001255188	Olr1012	Olfactory receptor 1012	0.01	2.3	-1.2	-2.8	---
7_490	gi_119903385	XM_599529	Ttc39d	Tetratricopeptide repeat domain 39d	0.00	2.5	-1.1	-2.8	---
7_491	gi_119922520	XM_001252579	LOC784191	Similar to seven transmembrane helix receptor	0.00	2.5	-1.1	-2.8	---
7_492	gi_149643056	NM_001099198	MGC165685	Hypothetical protein	0.01	2.2	-1.3	-2.8	---
7_493	gi_119892549	XM_001255334	LOC788199	Similar to olfactory receptor mor113-4	0.01	2.2	-1.2	-2.8	---
7_494	gi_119922221	XM_001254166	Olfr1199	Olfactory receptor 1199	0.00	2.7	1.0	-2.8	---
7_495	gi_119909126	XM_001252899	LOC784650	Similar to guanylate cyclase 70 kda subunit	0.01	2.3	-1.2	-2.8	---
7_496	gi_119925348	XM_001250814	LOC785762	Similar to prostaglandin f synthase-like1 protein	0.00	2.7	1.0	-2.8	---
7_497	gi_119922591	XM_001254813	LOC787405	Similar to t cell receptor delta chain	0.00	3.4	1.2	-2.8	---
7_498	gi_119922293	XM_610277	Gm4911	Predicted pseudogene 4911	0.00	2.6	-1.1	-2.8	---
7_499	gi_116004418	NM_001077100	MGC137454	Hypothetical protein	0.00	2.5	-1.1	-2.7	---
7_500	gi_119921172	XM_001255156	LOC787947	Similar to Cfolf4	0.01	2.3	-1.2	-2.7	---
7_501	gi_119924969	XM_590908	OR52N5	Olfactory receptor, family 52, subfamily n, member 5	0.00	2.5	-1.1	-2.7	---
7_502	gi_88319945	NM_176638	CASP4	Caspase 4, apoptosis-related cysteine peptidase	0.00	3.2	1.2	-2.7	---
7_503	gi_119894615	XM_868476	OR10H3	Olfactory receptor, family 10, subfamily h, member 3	0.00	2.5	-1.1	-2.7	---
7_504	gi_163915356	NM_001113173	CXCL11	Chemokine (c-x-c motif) ligand 11	0.01	2.4	-1.1	-2.7	---
7_505	gi_119907422	XM_592902	LOC514976	Similar to olfactory receptor hpfh1or	0.02	2.1	-1.3	-2.7	---
7_506	gi_119893818	XM_612659	LOC533295	Similar to y53f4b.4a	0.01	2.4	-1.1	-2.7	---
7_507	gi_115497081	NM_001075656	JUNB	Jun B proto-oncogene	0.00	3.9	1.4	-2.7	---
7_508	gi_116003880	NM_001076831	COL3A1	Collagen, type iii, alpha 1	0.00	2.7	1.0	-2.7	---
7_509	gi_119927416	XM_001256048	LOC789240	Similar to testis derived transcript	0.01	2.2	-1.2	-2.7	---
7_510	gi_119902188	XM_586246	OR4M1	Olfactory receptor, family 4, subfamily m, member 1	0.02	2.1	-1.3	-2.7	---
7_511	gi_76686152	XM_593724	OR4C46	Olfactory receptor, family 4, subfamily c, member 46	0.00	2.3	-1.2	-2.7	---
7_512	gi_119920037	XR_027774	LOC784212	Riken cDNA 1700014n06 gene	0.02	2.0	-1.3	-2.7	---
7_513	gi_76677166	XM_598492	OR6Y1	Olfactory receptor, family 6, subfamily y, member 1	0.00	2.4	-1.1	-2.7	---
7_514	gi_119902168	XM_001252434	OR11G2	Olfactory receptor, family 11, subfamily g, member 2	0.00	2.5	-1.1	-2.7	---
7_515	gi_31342172	NM_174376	KLRA1	Killer cell lectin-like receptor subfamily a pseudogene 1	0.00	3.2	1.2	-2.7	---
7_516	gi_119901504	XM_600290	Taar4	Trace amine-associated receptor 4	0.00	2.5	-1.1	-2.7	---
7_517	gi_41386789	NM_174356	IL12B	Interleukin 12b (natural killer cell stimulatory factor 2, cytotoxic lymphocyte maturation factor 2, p40)	0.00	2.3	-1.2	-2.7	---
7_518	gi_119884883	XM_606901	PRKCI	Protein kinase c, iota	0.00	2.6	-1.0	-2.7	---
7_519	gi_115497291	NM_001075634	TCIRG1	T-cell, immune regulator 1, atpase, h+ transporting, lysosomal v0 subunit a3	0.00	2.1	-1.3	-2.7	---
7_520	gi_31341616	NM_174579	POU1F1	Pou class 1 homeobox 1	0.01	2.2	-1.2	-2.7	---
7_521	gi_119925795	XM_586018	OR9G4	Olfactory receptor, family 9, subfamily g, member 4	0.01	2.4	-1.1	-2.7	---
7_522	gi_119928284	XM_580340	OR13C9	Olfactory receptor, family 13, subfamily c, member 9	0.00	2.6	1.0	-2.7	---
7_523	gi_119915640	XM_592285	Olr1768	Olfactory receptor 1768	0.01	2.4	-1.1	-2.7	---
7_524	gi_119926136	XM_599438	PM20D2	Peptidase m20 domain containing 2	0.00	4.2	1.6	-2.7	---
7_525	gi_134085852	NM_001083407	SPATA20	Spermatogenesis associated 20	0.00	2.4	-1.1	-2.7	---
7_526	gi_119901508	XM_001253652	TAAR2	Trace amine associated receptor 2	0.00	2.4	-1.1	-2.7	---
7_527	gi_61808422	XM_586491	OR4C6	Olfactory receptor, family 4, subfamily c, member 6	0.01	2.2	-1.2	-2.7	---
7_528	gi_76680252	XM_870182	LOC521256	Similar to olfactory receptor olfr566	0.01	2.2	-1.2	-2.7	---
7_529	gi_77404247	NM_001034041	SNCA	Synuclein, alpha (non a4 component of amyloid precursor)	0.00	2.5	-1.1	-2.7	---
7_530	gi_119903002	XM_867514	TTC8	Tetratricopeptide repeat domain 8	0.00	2.3	-1.2	-2.7	---
7_531	gi_119909243	XM_614458	CLIP1	Cap-gly domain containing linker protein 1	0.00	2.2	-1.2	-2.6	---
7_532	gi_149643100	NM_001098958	CDKN1A	Cyclin-dependent kinase inhibitor 1a (p21, cip1)	0.00	2.2	-1.2	-2.6	---
7_533	gi_119924383	XM_001254912	Olfr1124	Olfactory receptor 1124	0.00	2.5	-1.1	-2.6	---
7_534	gi_119920952	XM_870254	LOC617921	Similar to olfactory receptor mor14-2	0.01	2.2	-1.2	-2.6	---
7_535	gi_119907885	XM_001251593	LOC782933	Similar to olfactory receptor olfr987	0.03	2.0	-1.3	-2.6	---
7_536	gi_31342685	NM_174169	RGS7	Regulator of g-protein signaling 7	0.01	2.3	-1.2	-2.6	---
7_537	gi_31342076	NM_174411	PAG1B	Pregnancy-associated glycoprotein 1	0.02	2.1	-1.3	-2.6	---
7_538	gi_119902589	XR_027721	DMXL2	Dmx-like 2	0.00	2.4	-1.1	-2.6	---
7_539	gi_78050076	NM_001035055	CLDN11	Claudin 11	0.01	2.4	-1.1	-2.6	---
7_540	gi_158519826	NM_001110096	BEX4	Brain expressed, x-linked 4	0.02	2.2	-1.2	-2.6	---
7_541	gi_119921691	XM_001249303	LOC781004	Hypothetical protein	0.00	2.5	1.0	-2.6	---
7_542	gi_119921055	XM_001252743	Olr94	Olfactory receptor 94	0.01	2.3	-1.2	-2.6	---
7_543	gi_119889562	XM_001250696	LOC782107	Similar to KIAA0466 protein	0.00	2.1	-1.2	-2.6	---
7_544	gi_119911429	XR_027573	LOC783256	Similar to brca1-binding helicase-like protein bach1	0.00	2.5	-1.1	-2.6	---
7_545	gi_119915977	XM_588519	NFATC1	Nuclear factor of activated t-cells, cytoplasmic, calcineurin-dependent 1	0.00	2.5	1.0	-2.6	---
7_546	gi_119922217	XM_593873	Olfr1257	Olfactory receptor 1257	0.00	2.5	1.0	-2.6	---

7_547	gi_119923068	XM_001253958	LOC786202	Similar to olfactory receptor	0.02	2.2	-1.2	-2.6	---
7_548	gi_115495730	NM_001076164	PPP1R3C	Protein phosphatase 1, regulatory (inhibitor) subunit 3c	0.03	2.0	-1.3	-2.6	---
7_549	gi_164452934	NM_001080297	SWAP70	Swap-70 protein (swap70)	0.00	2.1	-1.2	-2.6	---
7_550	gi_119929994	XM_001256551	OR13F1	Olfactory receptor, family 13, subfamily f, member 1	0.00	2.6	1.0	-2.6	---
7_551	gi_119905975	XM_602010	NCOA3	Nuclear receptor coactivator 3	0.01	2.2	-1.2	-2.6	---
7_552	gi_116004376	NM_001077079	C1orf185	Chromosome 1 open reading frame 185	0.02	2.1	-1.2	-2.6	---
7_553	gi_27806988	NM_174545	GABRG2	Gamma-aminobutyric acid (gaba) a receptor, gamma 2	0.00	2.4	-1.1	-2.6	---
7_554	gi_119887145	XR_028592	SIK1	Salt-inducible kinase 1	0.00	2.1	-1.2	-2.6	---
7_555	gi_157428069	NM_001105473	STOM	Stomatin	0.00	4.0	1.6	-2.6	---
7_556	gi_116004410	NM_001077096	TNFRSF19	Tumor necrosis factor receptor superfamily, member 19	0.00	2.2	-1.1	-2.6	---
7_557	gi_119902141	XM_001252038	RNASE11	Ribonuclease, rnase a family, 11 (non-active)	0.02	2.1	-1.2	-2.5	---
7_558	gi_119922207	XM_001253994	LOC786248	Similar to c4bp alpha chain	0.03	2.0	-1.3	-2.5	---
7_559	gi_28603729	NM_176626	PAG18	Pregnancy-associated glycoprotein 18	0.00	2.4	-1.0	-2.5	---
7_560	gi_119912262	XM_001254120	KRTAP4-6	Keratin associated protein 4-6	0.02	2.1	-1.2	-2.5	---
7_561	gi_115497393	NM_001076097	SULT1C4	Sulfotransferase family, cytosolic, 1c, member 4	0.03	2.0	-1.3	-2.5	---
7_562	gi_94966908	NM_001040554	HLA-A	Major histocompatibility complex, class i, a	0.00	4.0	1.6	-2.5	---
7_563	gi_119929795	XM_581848	OR8A1	Olfactory receptor, family 8, subfamily a, member 1	0.01	2.2	-1.2	-2.5	---
7_564	gi_119928966	XM_001253584	LOC785624	Similar to olf4	0.01	2.2	-1.1	-2.5	---
7_565	gi_119922291	XM_001251181	MAGEB17	Melanoma antigen family b, 17	0.02	2.0	-1.2	-2.5	---
7_566	gi_119907553	XM_870343	Olfr561	Olfactory receptor 561	0.01	2.2	-1.1	-2.5	---
7_567	gi_119901893	XM_001251257	DENND4A	Denn/madd domain containing 4a	0.00	2.1	-1.2	-2.5	---
7_568	gi_119922801	XM_589238	Olfr594	Olfactory receptor 594	0.01	2.3	-1.1	-2.5	---
7_569	gi_119924375	XM_001254801	OR5F1	Olfactory receptor, family 5, subfamily f, member 1	0.01	2.3	-1.1	-2.5	---
7_570	gi_76651124	XM_870382	Olfr124	Olfactory receptor 124	0.01	2.2	-1.2	-2.5	---
7_571	gi_119927729	XM_001254998	Olfr143	Olfactory receptor 143	0.01	2.2	-1.1	-2.5	---
7_572	gi_134085628	NM_001083391	ELMO2	Engulfment and cell motility 2	0.00	2.3	-1.1	-2.5	---
7_573	gi_115496269	NM_001075993	ODF2L	Outer dense fiber of sperm tails 2-like	0.02	2.0	-1.2	-2.5	---
7_574	gi_119903020	XM_001251107	B2M	Beta-2-microglobulin	0.00	2.2	-1.2	-2.5	---
7_575	gi_119888344	XM_001252286	LOC784637	Similar to bicaudal d homolog 1 (drosophila)	0.02	2.0	-1.2	-2.5	---
7_576	gi_119891769	XM_001249423	INSIG1	Insulin induced gene 1	0.00	2.4	1.0	-2.5	---
7_577	gi_119915423	XR_028666	LOC788634	Similar to non-classical mhc class i antigen	0.01	2.1	-1.2	-2.5	---
7_578	gi_119889249	XM_581374	ADAR	Adenosine deaminase, RNA-specific	0.00	4.0	1.6	-2.5	---
7_579	gi_119892609	XM_867045	OR6C4	Olfactory receptor, family 6, subfamily c, member 4	0.01	2.2	-1.1	-2.5	---
7_580	gi_76616267	XM_870706	TRIM69	Tripartite motif containing 69	0.01	2.2	-1.1	-2.5	---
7_581	gi_118151015	NM_001077961	NDST3	N-deacetylase/n-sulfotransferase (heparan glucosaminyl) 3	0.00	2.3	-1.1	-2.4	---
7_582	gi_119915551	XM_871334	Olfr1535	Olfactory receptor 1535	0.01	2.2	-1.1	-2.4	---
7_583	gi_154152200	NM_001100309	TDRD7	Tudor domain containing 7	0.00	4.4	1.8	-2.4	---
7_584	gi_119921007	XM_001251527	LOC782875	Similar to olfactory receptor olr88	0.01	2.2	-1.1	-2.4	---
7_585	gi_119933559	XM_001256916	LOC790451	Hypothetical protein	0.00	2.4	1.0	-2.4	---
7_586	gi_119894398	XM_001253396	OR5W2	Olfactory receptor, family 5, subfamily w, member 2	0.01	2.4	1.0	-2.4	---
7_587	gi_149643024	NM_001099037	ABCD2	ATP-binding cassette, sub-family d (ald), member 2	0.02	2.1	-1.2	-2.4	---
7_588	gi_119895358	XM_582424	OR2L13	Olfactory receptor, family 2, subfamily l, member 13	0.01	2.2	-1.1	-2.4	---
7_589	gi_119912322	XM_618105	KRT39	Keratin 39	0.01	2.3	-1.0	-2.4	---
7_590	gi_119922011	XM_001253957	OR8K1	Olfactory receptor, family 8, subfamily k, member 1	0.02	2.1	-1.2	-2.4	---
7_591	gi_76253719	NM_001002892	ST3GAL2	ST3 beta-galactoside alpha-2,3-sialyltransferase 2	0.00	2.4	1.0	-2.4	---
7_592	gi_149642586	NM_001098900	SLC44A3	Solute carrier family 44, member 3	0.01	2.2	-1.1	-2.4	---
7_593	gi_157074077	NM_001103280	TRDN	Triadin	0.01	2.2	-1.1	-2.4	---
7_594	gi_119921413	XM_583529	LOC506992	Similar to olfactory receptor mor160-5	0.01	2.3	-1.0	-2.4	---
7_595	gi_119908975	XM_594035	SETD7	Set domain containing (lysine methyltransferase) 7	0.00	2.1	-1.1	-2.4	---
7_596	gi_56119115	NM_001007819	DDX4	Dead (asp-glu-ala-asp) box polypeptide 4	0.00	2.4	1.0	-2.4	---
7_597	gi_115495458	NM_001075504	NLGN3	Neuroligin 3	0.01	2.1	-1.1	-2.4	---
7_598	gi_155372024	NM_001101150	NOVA1	Neuro-oncological ventral antigen 1	0.01	2.1	-1.1	-2.4	---
7_599	gi_119895374	XM_001255418	LOC788323	Similar to olfactory receptor 2t29	0.00	2.4	1.0	-2.4	---
7_600	gi_119902186	XM_869270	LOC617084	Similar to seven transmembrane helix receptor	0.01	2.3	1.0	-2.4	---
7_601	gi_119930158	XM_869637	Olfr284	Olfactory receptor 284	0.01	2.3	1.0	-2.4	---
7_602	gi_84000380	NM_001038200	PRP8	Prolactin-related protein viii (PRP8)	0.01	2.2	-1.1	-2.4	---
7_603	gi_31341292	NM_176643	DDHD1	Ddhd domain containing 1	0.00	2.2	-1.1	-2.4	---
7_604	gi_119921234	XM_597790	RAPGEF5	Rap guanine nucleotide exchange factor (GEF) 5	0.02	2.0	-1.2	-2.4	---
7_605	gi_119892869	XM_871851	CAPRIN2	Caprin family member 2	0.00	2.3	-1.0	-2.4	---
7_606	gi_115496829	NM_001075309	GPM6A	Glycoprotein m6a	0.01	2.3	1.0	-2.3	---
7_607	gi_119895074	XM_001255309	LOC789216	Similar to interferon regulatory factor 1	0.00	4.5	1.9	-2.3	---
7_608	gi_149642938	NM_001098988	NEK6	Nima (never in mitosis gene a)-related kinase 6	0.00	3.5	1.5	-2.3	---
7_609	gi_119893961	XM_598333	RASSF6	Ras association (ralgds/af-6) domain family member 6	0.02	2.1	-1.1	-2.3	---
7_610	gi_31341715	NM_174543	GABRA4	Gamma-aminobutyric acid (gaba) a receptor, alpha 4	0.01	2.3	1.0	-2.3	---
7_611	gi_119923620	XM_609508	LOC531024	Similar to olfactory receptor olfr1197	0.01	2.3	1.0	-2.3	---
7_612	gi_62751672	NM_001015586	DSTN	Destrin (actin depolymerizing factor)	0.01	2.2	-1.1	-2.3	---
7_613	gi_147900717	NM_001098141	GYPA	Glycophorin a	0.01	2.2	-1.0	-2.3	---
7_614	gi_119912260	XM_001254106	LOC787090	Similar to hair keratin cysteine rich protein	0.02	2.1	-1.1	-2.3	---
7_615	gi_119875947	XM_615721	NCAM2	Neural cell adhesion molecule 2	0.02	2.1	-1.1	-2.3	---
7_616	gi_119879088	XM_001252292	OR5H1	Olfactory receptor, family 5, subfamily h, member 1	0.01	2.1	-1.1	-2.3	---
7_617	gi_119924185	XM_001255673	OR4D1	Olfactory receptor, family 4, subfamily d, member 1	0.01	2.1	-1.1	-2.3	---
7_618	gi_119892551	XM_001255346	OR6C74	Olfactory receptor, family 6, subfamily c, member 74	0.01	2.2	-1.0	-2.3	---
7_619	gi_119890804	XM_001252604	SNX13	Sorting nexin 13	0.01	2.1	-1.1	-2.3	---
7_620	gi_119892287	XM_001252824	LOC785803	Similar to lysozyme	0.01	2.3	1.0	-2.3	---
7_621	gi_119878574	XM_595936	CADM2	Cell adhesion molecule 2	0.01	2.1	-1.1	-2.3	---
7_622	gi_119921417	XM_586484	Olfr282	Olfactory receptor 282	0.01	2.1	-1.1	-2.3	---
7_623	gi_157279882	NM_001104987	MCTP1	Multiple c2 domains, transmembrane 1	0.01	2.2	-1.0	-2.3	---
7_624	gi_76623803	XM_586022	LOC509128	Similar to olfactory receptor, family 11, subfamily h, member 1	0.01	2.2	1.0	-2.3	---
7_625	gi_119928697	XM_593499	OR4K15	Olfactory receptor, family 4, subfamily k, member 15	0.01	2.1	-1.1	-2.3	---
7_626	gi_75832089	NM_181012	IDH1	Isocitrate dehydrogenase 1 (nadp+), soluble	0.02	2.0	-1.1	-2.3	---
7_627	gi_119895354	XM_001255231	OR2L3	Olfactory receptor, family 2, subfamily l, member 3	0.01	2.1	-1.1	-2.3	---
7_628	gi_119887420	XM_001254873	NCKAP1	Nck-associated protein 1	0.01	2.2	-1.0	-2.3	---
7_629	gi_119906534	XM_612068	PI15	Peptidase inhibitor 15	0.01	2.1	-1.0	-2.2	---
7_630	gi_119892977	XM_591626	KLRC1	Killer cell lectin-like receptor subfamily c, member 1	0.01	2.2	1.0	-2.2	---
7_631	gi_90991697	NM_001012285	LEPR	Leptin receptor	0.03	2.0	-1.1	-2.2	---
7_632	gi_119914208	XM_580659	LOC538493	Similar to chromosome 14 open reading frame 173	0.00	2.2	1.0	-2.2	---
7_633	gi_119922421	XM_602604	Olfr1134	Olfactory receptor 1134	0.02	2.1	-1.1	-2.2	---
7_634	gi_31342589	NM_174209	UACA	Uveal autoantigen with coiled-coil domains and ankyrin repeats	0.01	2.2	1.0	-2.2	---
7_635	gi_119901594	XM_600433	PHACTR2	Phosphatase and actin regulator 2	0.01	2.2	1.0	-2.2	---

7_636	gi_119891943	XM_001249912	LRRIQ1	Leucine-rich repeats and iq motif containing 1	0.01	2.1	-1.0	-2.2	---
7_637	gi_119918958	XM_583490	LOC506960	Similar to seven transmembrane helix receptor	0.02	2.1	1.0	-2.2	---
7_638	gi_119902535	XM_613448	PRTG	Protogenin	0.02	2.1	-1.0	-2.2	---
7_639	gi_119902733	XM_617753	LOC537580	Hypothetical protein	0.02	2.1	1.0	-2.2	---
7_640	gi_119921109	XM_001253971	LOC786219	Similar to t-cell receptor alpha chain	0.01	2.2	1.0	-2.2	---
7_641	gi_119921057	XM_001252809	LOC784539	Similar to olfactory receptor olfr598	0.03	2.0	-1.1	-2.2	---
7_642	gi_119907383	XM_610954	LOC532436	Similar to olfactory receptor mor31-7	0.02	2.0	-1.0	-2.1	---
7_643	gi_157074135	NM_001103310	LOC789977	Hypothetical protein	0.02	2.1	1.0	-2.1	---
7_644	gi_119892713	XR_028263	LOC505479	Similar to grp94 neighboring nucleotidase	0.02	2.1	1.0	-2.1	---
7_645	gi_76669664	XM_864501	OR6C75	Olfactory receptor, family 6, subfamily c, member 75	0.02	2.1	1.0	-2.1	---
7_646	gi_164420765	NM_001113247	CHN1	Chimerin (chimaerin) 1	0.03	2.0	-1.1	-2.1	---
7_647	gi_148229657	NM_181002	AOC3	Amine oxidase, copper containing 3 (vascular adhesion protein 1)	0.02	2.0	-1.0	-2.1	---
7_648	gi_119919308	XM_600752	BATF2	Basic leucine zipper transcription factor, atf-like 2	0.01	2.0	1.0	-2.1	---
7_649	gi_119900577	XM_617223	LOC537068	Similar to piwi-like 2 (drosophila)	0.02	2.1	1.0	-2.1	---
7_650	gi_77735964	NM_001034509	LOC515736	Caspase-15	0.02	2.0	1.0	-2.1	---

Supplementary data table 8 Full list of genes in profile 8 (n = 98)

Profile_ S. No	SEQ. ID	Acc. No. (RefSeq)	Putative Gene symbol	Gene Name	BL20 LPS Vs BL20 FDR (4h/or18)	BL20 LPS Vs BL20 Abs: FC (4h/or18)	TBL20 Vs BL20 Abs: FC	TBL20 Vs BL20 LPS Abs: FC (4h/or18)	BW720c Response
						↓	---		
8_1	gi_116004314	NM_001077046	PLIN3	Perilipin 3	0.00	-9.5	1.8	17.2	---
8_2	gi_119920411	XM_001252291	BCOR	BCL6 corepressor	0.00	-21.0	-1.4	15.2	---
8_3	gi_31340780	NM_174669	SERPINE2	Serpin peptidase inhibitor, clade e (nexin, plasminogen activator inhibitor type 1), member 2	0.00	-9.2	1.6	15.0	---
8_4	gi_119892844	XM_581489	FGD4	Fyve, rhogef and ph domain containing 4	0.00	-9.4	1.6	14.9	---
8_5	gi_116004122	NM_001076954	GOLGA7B	Golgin a7 family, member b	0.00	-5.7	1.9	10.8	---
8_6	gi_115495426	NM_001075876	CAMKV	Cam kinase-like vesicle-associated	0.00	-15.7	-1.5	10.6	---
8_7	gi_119913698	XM_868029	LOC616063	Similar to myeloid-associated differentiation marker	0.00	-8.3	1.2	9.7	---
8_8	gi_156120776	NM_001102065	NID2	Nidogen 2 (osteonidogen)	0.00	-8.7	1.0	9.0	---
8_9	gi_119908995	XM_602397	SLC7A11	Solute carrier family 7, member 11	0.00	-9.3	-1.1	8.8	---
8_10	gi_119888246	XR_027752	PLCL1	Phospholipase c-like 1	0.00	-12.5	-1.4	8.8	---
8_11	gi_119922570	XM_001254638	ARMCX6	Armadillo repeat containing, x-linked 6	0.00	-12.3	-1.5	8.5	---
8_12	gi_119893809	XM_613340	RBM47	Rna binding motif protein 47	0.00	-4.5	1.8	8.1	---
8_13	gi_148230339	NM_174557	IGFBP4	Insulin-like growth factor binding protein 4	0.00	-4.4	1.8	7.9	---
8_14	gi_31340894	NM_174766	TNS1	Tensin 1	0.00	-10.8	-1.6	6.6	---
8_15	gi_119908122	XM_001252887	RGS1	Regulator of g-protein signaling 1	0.00	-5.6	1.2	6.6	---
8_16	gi_31341563	NM_174601	SLC1A5	Solute carrier family 1 (neutral amino acid transporter), member 5	0.00	-3.6	1.8	6.5	---
8_17	gi_78369387	NM_001035353	PLVAP	Plasmalemma vesicle associated protein	0.00	-4.3	1.5	6.5	---
8_18	gi_150247066	NM_001099366	SPRY1	Sprouty homolog 1, antagonist of FGF signaling (drosophila)	0.00	-5.0	1.3	6.5	---
8_19	gi_154152126	NM_001100354	OLFML2B	Olfactomedin-like 2b	0.00	-3.9	1.6	6.4	---
8_20	gi_156120814	NM_001102084	NELL2	Nel-like 2 (chicken)	0.00	-4.9	1.3	6.4	---
8_21	gi_115497431	NM_001075560	NUDT16	Nudix (nucleoside diphosphate linked moiety x)-type motif 16	0.00	-6.2	1.0	6.3	---
8_22	gi_119879733	XM_614853	ETV5	ETS variant 5	0.00	-4.4	1.4	6.2	---
8_23	gi_156718123	NM_001103097	F2R	Coagulation factor ii (thrombin) receptor	0.00	-4.8	1.2	6.0	---
8_24	gi_115494955	NM_001075853	PDK2	Pyruvate dehydrogenase kinase, isozyme 2	0.00	-4.4	1.3	5.8	---
8_25	gi_157954058	NM_001109806	FCGR2B	Fc fragment of igg, low affinity iib, receptor (CD32)	0.00	-5.1	1.1	5.4	---
8_26	gi_134085719	NM_001083488	FAM107A	Family with sequence similarity 107, member a	0.00	-2.9	1.8	5.2	---
8_27	gi_157074119	NM_001103302	LOC618633	Similar to myeloid-associated differentiation marker	0.00	-2.8	1.8	5.1	---
8_28	gi_116004140	NM_001076960	ARMCX2	Armadillo repeat containing, x-linked 2	0.00	-3.5	1.5	5.1	---
8_29	gi_77736420	NM_001034738	LTB4R	Leukotriene b4 receptor	0.00	-3.2	1.6	5.0	---
8_30	gi_31343314	NM_173899	CD5	CD5 molecule	0.00	-4.9	1.0	4.8	---
8_31	gi_148237174	NM_001098148	KIAA1383	KIAA1383	0.00	-2.6	1.8	4.8	---
8_32	gi_119930243	XM_585571	PCSK4	Proprotein convertase subtilisin/kexin type 4	0.00	-2.9	1.6	4.7	---
8_33	gi_76657936	XM_597134	LGALS12	Lectin, galactoside-binding, soluble, 12	0.00	-5.5	-1.2	4.6	---
8_34	gi_119906547	XR_027912	LOC535166	Similar to sulfatase 1	0.00	-2.6	1.7	4.6	---
8_35	gi_119903494	XM_595079	EML6	Echinoderm microtubule associated protein like 6	0.00	-6.3	-1.4	4.5	---
8_36	gi_150247133	NM_001099371	HPDL	4-hydroxyphenylpyruvate dioxygenase-like	0.00	-3.2	1.4	4.5	---
8_37	gi_119887452	XM_874476	GPR155	G protein-coupled receptor 155	0.00	-4.0	1.1	4.5	---
8_38	gi_119904602	XM_582091	STARD13	Star-related lipid transfer (start) domain containing 13	0.00	-4.5	-1.0	4.4	---
8_39	gi_114052243	NM_001046358	CMTM8	Cklf-like marvel transmembrane domain containing 8	0.01	-2.1	2.0	4.2	↓
8_40	gi_31343172	NM_173962	SV2A	Synaptic vesicle glycoprotein 2a	0.00	-5.5	-1.3	4.2	---
8_41	gi_126158902	NM_001081577	SLC1A4	Solute carrier family 1 (glutamate/neutral amino acid transporter), member 4	0.00	-3.2	1.3	4.2	---
8_42	gi_119912114	XM_614523	NXPH3	Neurexophilin 3	0.00	-3.3	1.2	4.1	---
8_43	gi_148238184	NM_001098020	TLE1	Transducin-like enhancer of split 1 (e(sp1) homolog, drosophila)	0.00	-2.2	1.9	4.1	---
8_44	gi_119901706	XM_596626	SYNJ2	Synaptojanin 2	0.01	-2.1	1.9	4.0	---
8_45	gi_156121146	NM_001102251	Jakmip1	Janus kinase and microtubule interacting protein 1	0.00	-3.2	1.2	3.8	---
8_46	gi_119891341	XM_868371	PODXL	Podocalyxin-like	0.00	-4.7	-1.3	3.8	---
8_47	gi_149642966	NM_001099031	POC1B	Poc1 centriolar protein homolog b (chlamydomonas)	0.00	-5.2	-1.4	3.8	---
8_48	gi_156120560	NM_001101956	MGC152278	Similar to myeloid-associated differentiation marker	0.00	-3.3	1.1	3.7	---
8_49	gi_119895490	XM_601210	HBEGF	Heparin-binding egf-like growth factor	0.00	-2.6	1.4	3.6	---
8_50	gi_119892809	XM_615197	CARD10	Caspase recruitment domain family, member 10	0.00	-4.2	-1.2	3.6	---
8_51	gi_114051791	NM_001045971	SPINT2	Serine peptidase inhibitor, kunitz type, 2	0.00	-2.5	1.4	3.6	---
8_52	gi_114051388	NM_001046175	MAP1LC3A	Microtubule-associated protein 1 light chain 3 alpha	0.01	-2.1	1.7	3.5	---
8_53	gi_119887600	XM_615555	GALNT3	Udp-n-acetyl-alpha-d-galactosamine:polypeptide n-acetylgalactosaminyltransferase 3	0.01	-2.2	1.6	3.5	---
8_54	gi_119927446	XM_868985	LOC616864	Similar to proton/amino acid transporter 4	0.01	-2.1	1.7	3.5	---

8_55	gi_119920662	XM_586585	FRMPD4	Ferm and pdz domain containing 4	0.00	-2.6	1.3	3.5	---
8_56	gi_114050922	NM_001046448	TSPAN14	Tetraspanin 14	0.00	-2.9	1.2	3.5	---
8_57	gi_31342305	NM_174331	GRK5	G protein-coupled receptor kinase 5	0.00	-3.4	1.0	3.5	---
8_58	gi_62460585	NM_001014947	MAPK13	Mitogen-activated protein kinase 13	0.00	-4.5	-1.3	3.4	---
8_59	gi_76665844	XM_609088	AADACL3	Arylacetamide deacetylase-like 3	0.00	-5.7	-1.7	3.4	---
8_60	gi_115497717	NM_001075593	PLEKHA1	Pleckstrin homology domain containing, family a (phosphoinositide binding specific) member 1	0.01	-2.1	1.6	3.4	---
8_61	gi_116004188	NM_001076984	GLIPR1	Gli pathogenesis-related 1	0.00	-4.3	-1.3	3.4	---
8_62	gi_115495864	NM_001075533	ST6GALNAC2	St6 (alpha-n-acetyl-neuraminyl-2,3-beta-galactosyl-1,3)-n-acetylgalactosaminide alpha-2,6-sialyltransferase 2	0.00	-3.3	1.0	3.4	---
8_63	gi_115495270	NM_001075169	SORBS1	Sorbin and sh3 domain containing 1	0.00	-2.8	1.2	3.4	---
8_64	gi_126723520	NM_001082439	IGSF8	Immunoglobulin superfamily, member 8	0.02	-2.0	1.6	3.3	---
8_65	gi_31342040	NM_174422	PDE6H	Phosphodiesterase 6h, cgmp-specific, cone, gamma	0.01	-2.2	1.5	3.2	---
8_66	gi_119929752	XM_872864	ARRB2	Arrestin, beta 2	0.00	-3.4	-1.1	3.2	---
8_67	gi_116686101	NM_001077402	FCGR3A	Fc fragment of igg, low affinity iiia, receptor (CD16A)	0.00	-2.7	1.2	3.2	---
8_68	gi_119930626	XM_870478	TLE2	Transducin-like enhancer of split 2 (e(sp1) homolog, drosophila)	0.00	-3.1	1.0	3.2	---
8_69	gi_31342110	NM_174398	NCALD	Neurocalcin delta	0.00	-3.1	1.0	3.2	---
8_70	gi_157785606	NM_001105637	MAF	V-maf musculoaponeurotic fibrosarcoma oncogene homolog (avian)	0.00	-2.4	1.3	3.1	---
8_71	gi_76611568	XM_869196	E2F2	E2f transcription factor 2	0.01	-2.1	1.5	3.1	---
8_72	gi_115495180	NM_001076537	TRIM9	Tripartite motif containing 9	0.00	-3.2	1.0	3.1	---
8_73	gi_78365245	NM_001035418	ARHGEF9	Cdc42 guanine nucleotide exchange factor (Gef) 9	0.00	-3.0	1.0	3.1	---
8_74	gi_156121042	NM_001102198	Slc19a3	Solute carrier family 19 , member 3	0.01	-2.1	1.5	3.1	---
8_75	gi_119909968	XM_870732	GPT2	Glutamic pyruvate transaminase 2	0.00	-2.8	1.1	3.0	---
8_76	gi_119909938	XR_028750	SPIRE2	Spire homolog 2 (drosophila)	0.00	-2.4	1.2	3.0	---
8_77	gi_115497811	NM_001076453	SLC19A1	Solute carrier family 19 (folate transporter), member 1	0.00	-2.4	1.2	3.0	---
8_78	gi_154152082	NM_001100341	C1orf74	Chromosome 1 open reading frame 74	0.00	-4.4	-1.5	3.0	---
8_79	gi_155371832	NM_001101051	MFNG	Mfng o-fucosylpeptide 3-beta-n-acetylglucosaminyltransferase	0.00	-3.1	-1.1	2.9	---
8_80	gi_115496721	NM_001075152	TMC4	Transmembrane channel-like 4	0.01	-2.2	1.3	2.9	---
8_81	gi_123858767	NM_001080366	PJA1	Praja ring finger 1	0.00	-2.7	1.1	2.9	---
8_82	gi_119911133	XM_001256455	C19orf51	Chromosome 19 open reading frame 51	0.00	-2.6	1.1	2.9	---
8_83	gi_156523107	NM_001102498	NKAPL	NFkB activating protein-like	0.00	-2.5	1.1	2.9	---
8_84	gi_41386781	NM_174231	ADRB2	Adrenergic, beta-2-, receptor, surface	0.00	-2.5	1.1	2.8	---
8_85	gi_119915572	XM_588389	ZKSCAN4	Similar to zinc finger protein 307, transcript variant 1	0.00	-3.2	-1.1	2.8	---
8_86	gi_119912967	XM_596546	SH3PXD2B	Sh3 and px domains 2b	0.00	-2.8	1.0	2.8	---
8_87	gi_164420752	NM_001113245	TRAF3IP3	TRAF3 interacting protein 3	0.00	-4.6	-1.7	2.8	---
8_88	gi_94966796	NM_001040496	ART3	Adp-ribosyltransferase 3	0.01	-2.1	1.3	2.8	---
8_89	gi_119896289	XM_867351	KIAA1797	Kiaa1797	0.00	-2.4	1.1	2.7	---
8_90	gi_119914579	XM_001250879	ARHGEF3	Rho guanine nucleotide exchange factor (GEF) 3	0.00	-4.7	-1.8	2.6	---
8_91	gi_119912784	XM_001253301	MXRA7	Matrix-remodelling associated 7	0.00	-2.2	1.2	2.6	---
8_92	gi_115496251	NM_001075741	GPRC5B	G protein-coupled receptor, family c, group 5, member b	0.00	-2.4	1.0	2.5	---
8_93	gi_56710322	NM_001008664	TLR3	Toll-like receptor 3	0.00	-4.8	-2.0	2.4	---
8_94	gi_118150831	NM_001077858	CTPS	Ctp synthase	0.00	-2.2	1.1	2.4	---
8_95	gi_119887519	XM_607085	PDK1	Pyruvate dehydrogenase kinase, isozyme 1	0.01	-2.1	1.1	2.4	---
8_96	gi_78042497	NM_001035017	PHGDH	Phosphoglycerate dehydrogenase	0.00	-2.3	1.0	2.3	---
8_97	gi_119918586	XM_586457	FUT11	Fucosyltransferase 11 (alpha (1,3) fucosyltransferase)	0.01	-2.0	1.1	2.2	---
8_98	gi_157954050	NM_001109796	CARD11	Caspase recruitment domain family, member 11	0.01	-2.0	1.0	2.1	---

Supplementary data table 9: Details of primers used for semi-quantitative RT-PCR and real-time quantitative RT-PCR

Gene symbol	GenBank accession number	F / R	Primer sequences (5' → 3')	Len. (bp)	Amp. length (bp)	Tm (˚C)	Gene name
ACTB	NM_173979	F	CTCTCTTCCAGCCTTCCTTCCTGG	24	240	58.21	ACTB actin, beta
		R	ACACGGAGTACTTGCGCTCAGG	22		58.92	
ANGPT4	NM_001076483	F	ACAATGACAACTGCCTCTGCAAGTGC	26	214	60.39	Angiopoietin 4
		R	GCTGCCCCTTAGATGCCCGAG	21		59.31	
AQP3	NM_001079794	F	TACCCCTCTGGACACTTGGACATGG	25	242	59.42	Aquaporin 3 (Gill blood groUp)
		R	CCGGCGATGGCGGTGAAAAGG	21		60.96	
BCL9	XR_028680	F	TACCCCTAAAGCACTCCCTGGCC	23	214	59.67	B-cell CLL/lymphoma 9
		R	CGAGTGTGGTGTGTGCTGGGAATC	24		60	
BCOR	NM_001191544	F	AGGTGTGCAAATTCAGCCCCACTG	24	244	59.88	Similar to BCL-6 corepressor
		R	ATGTCTGGGGTGACCTGGTCGG	22		59.92	
BIRC3	NM_001035293	F	GGATGATGCTATGTCAGAACACCTGAG	27	255	57.24	Baculoviral IAP repeat-containing 3
		R	TCCCAACACCTCAGTCCACCATC	23		58	
BOLA-DYA	NM_001012678	F	CTTGGGTGCTGCCTCCAGACGT	22		61.01	BOLA-DYA major histocompatibility complex, class II, DY alpha
		R	TGGAAGGAGCTCTGTGATGAAGTGG	25	248	58.23	
CALD1	NM_174258	F	CCTACCAGCGGAATGACGATGACG	24	230	59.49	Caldesmon 1
		R	CCGAGCCAGTCGCTCCAGG	19		59.17	
CCL4	NM_001075147	F	CCTTCTGTTCTCCAGCGCTCTCAG	24	192	59.08	Chemokine (C-C motif) ligand 4
		R	GCTCACTGGGGTTGGCGCAG	20		60.59	
CCL3	NM_174511	F	AGCAAGCACCGAGTCCCACCTC	22	244	60.75	chemokine (C-C motif) ligand 3
		R	CTTGGAGCACTGGCTGCTGGTC	22		59.99	
CD36	NM_174010	F	TGTGCAGAATCCAGATGAAGTGACAG	26	225	57.18	CD36 molecule (thrombospondin receptor)
		R	GCTGCTACAGCCAGGTTGAGAATG	24		58.08	
CD69	NM_174014	F	ACTGTAGGTTGGCTCCACGTGG	22	288	58.51	CD69 molecule
		R	TGGTGGCGCTGATGATGCATATTG	24		58.19	
CFLAR	NM_001012281	F	CTGTCTTCTGTGAGCTTGGCTGAGC	25	237	59.78	CASP8 and FADD-like apoptosis regulator
		R	ATCCTTGGCTATCTTGCTTCGACCCG	26		60.61	
CLU	NM_173902	F	CTCCAGTCCTACCAGCAGAAGATGC	25	279	58.65	Clusterin
		R	CAGTGCTTTCTCTGCCACATTCTCC	25		58.1	
CTTN	NM_001075287	F	TTGTGAGCGTCTCGCATCTGCACG	24	224	62.04	CTTN cortactin
		R	AACACATTCTCCCGCAGCTTGTGG	24		59.94	
CXCL10	NM_001046551	F	TGCAAGTCAATCCTGCCCACGTG	23	203	59.93	CXCL10 chemokine (C-X-C motif) ligand 10
		R	GAGGTAGCTTCTCTCTGGTCCATCC	25		57.67	
CX3CR1	NM_001102558	F	AGGAAGACTGACGGGCACATCG	22	243	58.66	CX3CR1 chemokine (C-X3-C motif) receptor 1
		R	TTGATGAGGGCAAACACCACCAGC	24		59.88	
CYP4F3	NM_001046391	F	AGCACTCGTCTGGACATGTTTGAAC	25	202	57.77	Cytochrome P450, family 4, subfamily F, polypeptide 3
		R	CATCGCGGGTGAGGTAGTACAGG	23		58.61	
DTX4	XM_606934	F	GCAGTGTCCGACCTGTAAGACC	22	216	57.03	Deltex homolog 4
		R	AGGTAACAATGTCGAGGGAAGCCAC	25		58.76	
ICAM1	NM_174348	F	TGGCCCTGCTCGGGACTCTG	20	191	60.25	ICAM1 intercellular adhesion molecule 1
		R	AACCTTCCAGTTGTCCCCACGG	22		58.69	
IFIT3	NM_001075414	F	GGCAGGCAAACAGCCATCATGAG	23	239	58.78	Interferon-induced

		R	CGCAAGCATTCCAGGGCTGC	20		59.22	protein with tetratricopeptide repeats 3
IGJ	NM_175773	F	TCCTAGTCAAGACATTGTGGAGAG	24	227	53.57	Iimmunoglobulin J chain
		R	TGTCATAGGTGTAACAGGTCTCAG	24		53.58	
IL2RB	XM_587301	F	ACTCACTCCAGGTCGTCCGCATAG	24	217	59.82	IL2RB interleukin 2 receptor, beta
		R	ACCTGAAGCTCATATTCCGTGCCTG	25		58.87	
IL15	NM_174090	F	GAGAAGTACTTCCATCCAGTGCTAC	25	255	54.76	Interleukin 15
		R	AGGAGAAAGCACTGCATCGCTG	22		57.59	
IL18	NM_174091	F	GGAATCAGATCACTTTGGCAAACTTGAACC	30	202	58.99	Interleukin 18
		R	GATGGTTACGGCCAGACCTCTAGTG	25		58.43	
ISG15	NM_174366	F	TGCAGAGAGCCTGGCACCAGAAC	23	268	60.97	ISG15 ubiquitin-like modifier
		R	GATGCAGTTCTGCACCACCAGC	22		58.67	
LMO2	NM_001076352	F	CTACTACAAGCTGGGCCGGAAGC	23	217	59.33	LIM domain only 2 (rhombotin-like 1)
		R	CACGATGTCGGAGTTGATGAGGAGG	25		58.95	
MFAP2	NM_174388	F	CCGCGTCTACGTCGTCAATAAGG	23	169	57.71	Microfibrillar-associated protein 2
		R	GCAGCCCCCACAGCTCCTG	19		59.67	
MEIS3	NM_001193152	F	CGCTGCTAGCCCTGGTCTTTGAG	23	232	59.57	MEIS3 similar to Meis1, myeloid ecotropic viral integration site 1 homolog 3
		R	AGGTGGAAGCGGAGCACTTGAATG	24		59.47	
MMP13	NM_174389	F	TGGTGACTTCTACCCATTTGATGG	24	275	54.56	MMP13 matrix metallopeptidase 13 (collagenase 3)
		R	ACTGAATCCCTTGGACATCATCATC	25		54.66	
MPEG1	NM_001046464	F	AAGGATCAAGCTGTAACTACCCAGG	25	243	56.03	Macrophage expressed gene 1
		R	CCTGATGTGGTCCTCCTGAATGAG	24		56.5	
MX2	NM_173941	F	CAAGAGGCACACTCAGACTTCCACT	25	256	58.42	MX2 myxovirus (influenza virus) resistance 2 (mouse)
		R	CCGCACCTTCTCCTCATACTTTCTG	25		57.2	
MYL4	NM_001075149	F	ATGCCGAGGTGCTGCGTGTCC	21	208	62.01	Myosin, light chain 4, alkali;
		R	GCAAGGACGTGGCGAAGCTCAG	22		60.61	
NDRG1	NM_001035009	F	AGCAGGACATTGAGACTTTGCATGGC	26	222	60.01	N-myc downstream regulated 1
		R	TGGGGAAGGACGCAGCGCC	19		61.85	
NID2	NM_001102065	F	CACTCCCACCCACGCCTTTCTG	22	261	59.67	Nidogen 2 (osteonidogen)
		R	CTATTCAGGTCATCAACCTCTCCTCGG	27		58.12	
PAK1	NM_001076898	F	CGACAGAAACCGCAGGCAGAGATG	24	249	60.12	p21 protein (Cdc42/Rac)-activated kinase 1
		R	CAGGTAAGATGGCTCGGTAAAATCGG	26		57.64	
PGM5	NM_001102335	F	TGCCCAACTTCATCCAGAGCGTGC	24	295	61.75	Phosphoglucomutase 5
		R	GCAACATTAAACTTCACTCCAAACTCTCC	29		57.07	
PIK3R6	NM_001102028	F	ACTCTAATGTACGTGCTCACTAAGGC	26	205	56.6	phosphoinositide-3-kinase, regulatory subunit 6
		R	CACTCGTCAGGTTCTGCTCGG	21		57.25	
RGS1	NM_001199063	F	GTGAGGAGAATATTGAGTTCTGGCTGG	27	321	57.18	Similar to regulator of G-protein signalling 1,
		R	AGCCAGGAGCCAGTCACTTTAGAC	24		58.16	
RNASEL	NM_001098165	F	GACTGAGGACGAGGCATCTACG	22	243	56.42	Ribonuclease L
		R	GGGTTGTTATGGCTCTCAGTCTCC	24		56.75	
Runx-2	XM_002684501	F	GACAGAAGCTTGATGACTCTAAACCTAG	28	224	55.33	Runt-related transcription factor 2
		R	CGAGGGATAGGACTGGTCATAGG	23		55.17	
RYK	XM_001249766	F	CCAGACACCCTACGTGGACATCG	23	238	58.79	Similar to receptor-like tyrosine kinase
		R	TCCTGACGCTGTGAGACTGTGAC	23		58.36	
SELL	NM_174182	F	ATGCAGCGGCCATGGACAATGTG	23	211	60.6	SELL selectin L
		R	AATCATGTCTGTTCCCTTGGAGCAG	25		57.03	
SLC6A6	NM_174610	F	CGTACCCCTGACCTACAACAAAGTCTAC	28	266	58.51	Solute carrier family

		R	CAATGATGTGCGTGGGTTTCCTGAG	25		58.62	6 (neurotransmitter transporter, taurine), member 6
**STAP1**	NM_001077868	F	ACTGCTCTACCCTTGTACTTTGAAG	25	177	54.53	Signal transducing adaptor family member 1
		R	CTGATCAGTAAGGCATGTAAGGTC	24		53.07	
**TLR4**	NM_174198	F	TGGACCTGAGCTTTAACTACCTG	23	159	59.39	TLR4 toll-like receptor 4
		R	TTCCCGTCAGTATCAAGGTG	20		58.8	
**VCAM1**	NM_174484	F	CAGGCACCTTCAGGAGGGACAG	22	226	58.57	Vascular cell adhesion molecule 1
		R	CAGCCTGTTGTGCTGCAAGTCAGTG	25		60.89	
**XCL1**	NM_175716	F	TCAGCCTCTTACTGCACAGCTCAG	24	264	58.48	XCL1 chemokine (C motif) ligand 1
		R	CAGGCAGCTTGAGGATCAGCACAG	24		59.83	
